# Supplementary material for: Epigenetic Mechanisms Are Involved in the Oncogenic Properties of ZNF518B in Colorectal Cancer
Source: Cancers (Basel). 2021 Mar 21;13(6):1433. doi: 10.3390/cancers13061433 (PMC8004037; doi:10.3390/cancers13061433)
Supplement: Supplementary file 1 [file cancers-13-01433-s001.pdf]

Epigenetic mechanisms are involved in the oncogenic properties of *ZNF518B* in colorectal cancer

Francisco Gimeno-Valiente, Ángela L. Riffo-Campos, Luis Torres, Noelia Tarazona, Valentina Gambardella, Andrés Cervantes, Gerardo López-Rodas, Luis Franco and Josefa Castillo

SUPPLEMENTARY METHODS

1. Selection of genomic sequences for ChIP analysis

To select the sequences for ChIP analysis in the five putative target genes, namely, *PADI3*, *ZDHHC2*, *RGS4*, *EFNA5* and *KAT2B*, the genomic region corresponding to the gene was downloaded from Ensembl. Then, zoom was applied to see in detail the promoter, enhancers and regulatory sequences. The details for HCT116 cells were then recovered and the target sequences for factor binding examined. Obviously, there are not data for ZNF518B, but special attention was paid to the target sequences of other zinc-finger containing factors. Finally, the regions that may putatively bind ZNF518B were selected and primers defining amplicons spanning such sequences were searched out. Supplementary Figure S3 gives the location of the amplicons used in each gene.

2. Obtaining the raw data and generating the BAM files for *in silico* analysis of the effects of *EHMT2* and *EZH2* silencing

The data of siEZH2 (SRR6384524), siG9a (SRR6384526) and siNon-target (SRR6384521) in HCT116 cell line, were downloaded from SRA (Bioproject PRJNA422822, https://www.ncbi.nlm.nih.gov/bioproject/), using SRA-tolkit (https://ncbi.github.io/sra-tools/). All data correspond to RNAseq single end.

```
  doBasics = TRUE
  doAll  = FALSE
$ fastq-dump -I --split-files SRR6384524
```

Data quality was checked using the software fastqc (https://www.bioinformatics.babraham.ac.uk /projects/fastqc/). The first low quality removing nucleotides were removed using FASTX-Toolkit (http://hannonlab.cshl.edu/fastxtoolkit/).

```
$ fastqc HCT116_PBS_siEZH2.fastq
$ fastx_trimmer -i HCT116_PBS_siEZH2.fastq
  -o HCT116_PBS_siEZH2_1.fastq -f 15 -Q 33 –v
```

The fastq were mapped using STAR, indexing the human genome version GRCH38.p12.

Then, the resulting SAM files were compressed to BAM format and sorted, using SAMtools (http://samtools.sourceforge. net/):

```
$  ./STAR –runMode genomeGenerate --runThreadN 10 --genomeDir
      ./ --genomeFastaFiles GRCh38.p12.genome.fa
$  ./STAR --genomeDir ./ --runThreadN 10 starIndex --readFilesIn
      /home/HCT116_PBS_siEZH2_1.fastq --outFileNamePrefix
/home/HCT116_siEZH2

$ samtools view -S -b HCT116_siEZH2Aligned.out.sam > HCT116_siEZH2.bam
$ samtools sort HCT116_siEZH2.bam -o HCT116_siEZH2_sort.bam
```

The fastq files also were mapped using Bowtie2 (httphttp://bowtie-bio.sourceforge.net/bowtie2/index.shtml).

```
$ ./bowtie2-build -f /mnt/sdb1/Bowtie2/GRCh38.p12.genome.fa human
$ ./bowtie2 -x human -U /mnt/sdb1/Fastq/HCT116_PBS_siEZH2_1.fastq -S
siEZH2.sam
$ samtools view -S -b siEZH2.sam > siEZH2.bam
$ samtools sort siEZH2.bam -o siEZH2_sort.bam
```

2. Count and matrix generation

The libraries used for the analysis were:

```
library(Rsamtools)
library(GenomicFeatures)
library(GenomicAlignments)
library(org.Hs.eg.db)
library(edgeR)
library(VennDiagram)
```

2.1. Generation of the STAR expression matrix

For siG9A in HCT116 cell lines:

```
gtfFile = "gencode.v29.chr_patch_hapl_scaff.annotation.gtf"
txdb = makeTxDbFromGFF(gtfFile, format="gtf")
genes = exonsBy(txdb, by="gene")
indir = getwd()
files = list.files(indir, pattern = '*.bam')
#[1] "HCT116_siG9A_sort.bam""HCT116_siNontarget_sort.bam"
bamLst = BamFileList(files, index=character(),
            obeyQname=TRUE)
# Counting reads
siG9A = summarizeOverlaps(features = genes,
                           reads = bamLst,
                        mode=”Union”,
                        singleEnd=TRUE,
                        ignore.strand=TRUE
                        fragments=FALSE)


colnames(siG9A)
#[1] "HCT116_siG9a_sort.bam""HCT116_siNontarget_sort.bam"
SampleName = c("siG9a","siNon")
Stage =c("siG9a","siNon")
```

```
colData(siG9a) = DataFrame(SampleName, Stage)
dim(siG9a)
# [1] 64837      2
save(siG9a,file="siG9a.rda")
```

For siEZH2 in HCT116 cell lines:

```
indir = getwd()
files = list.files(indir, pattern = '*.bam')
# [1] "HCT116_siEZH2_sort.bam""HCT116_siNontarget_sort.bam"
bamLst = BamFileList(files, index=character(),
                        obeyQname=TRUE)
siEZH2 = summarizeOverlaps(features = genes,read=bamLst,
                              mode="Union",
                              singleEnd=TRUE,
                              ignore.strand=TRUE,
                              fragments=FALSE)
colnames(siEZH2)
# [1] "HCT116_siEZH2_sort.bam""HCT116_siNontarget_sort.bam"
SampleName = c("siEZH2","siNon")
Stage =c("siEZH2","siNon")
colData(siEZH2) = DataFrame(SampleName,  Stage)
dim(siEZH2)
# [1] 64837        2
save(siEZH2,file="siEZH2.rda")
```

2.1. Generation of the Bowtie2 expression matrix

For siEZH2 in HCT116 cell line:

```
gtfFile = "gencode.v29.chr_patch_hapl_scaff.annotation.gtf"
txdb = makeTxDbFromGFF(gtfFile, format="gtf")
genes = exonsBy(txdb, by="gene")
indir = getwd()
files = list.files(indir, pattern = '*.bam')
# [1] "siEZH2_sort.bam""siNonTarget_sort.bam"
bamLst = BamFileList(files, index=character(),
                            obeyQname=TRUE)
siEZH2_Bowtie = summarizeOverlaps(features = genes,
                                    read=bamLst,
                                    mode="Union",
                                    singleEnd=TRUE,
                                    ignore.strand=TRUE,
                                    fragments=FALSE)
colnames(siEZH2_Bowtie)
# [1] "siEZH2_sort.bam""siNonTarget_sort.bam"
SampleName = c("siEZH2","siNon")
Stage =c("siEZH2","siNon")
colData(siEZH2_Bowtie) = DataFrame(SampleName, Stage)
dim(siEZH2_Bowtie)
# [1] 64837    2
save(siEZH2_Bowtie,file="siEZH2_Bowtie.rda")
```

For siG9a in HCT116 cell line:

```
indir = getwd()
files = list.files(indir, pattern = '*.bam')
# [1] "siG9a_sort.bam""siNonTarget_sort.bam"
bamLst = BamFileList(files, index=character(),
                          obeyQname=TRUE)
 siG9a_Bowtie = summarizeOverlaps(features = genes,
                                    read=bamLst,
                                    mode="Union",
                                    singleEnd=TRUE,
                                    ignore.strand=TRUE,
                                    fragments=FALSE)
 colnames(siG9a_Bowtie)
 #[1]"siG9a_sort.bam"     "siNonTarget_sort.bam"
 SampleName = c("siG9a","siNon")
 Stage =c("siG9a","siNon")
 colData(siG9a_Bowtie)=DataFrame(SampleName,Stage)
 dim(siG9a_Bowtie)
 # [1] 64837    2
 save(siG9a_Bowtie,file="siG9a_Bowtie.rda")
```

3. Normalisation and statistical analysis

3.1 STAR-edgeR

The statistical analisis was make acording to the user guide of edgeR package (https://www.bioconductor.org/packages/release/bioc/vignettes/edgeR/inst/doc/edgeRUsersGuide). In this case, we have one library for each treatment condition. Thus, the biological variability of 0.01 was assigned, as recommended in case of technical replicates.

For siEZH2 in HCT116 cell line, the procedure was:

```
load(file="siEZH2.rda")
#  edgeR user guide: 2.11       What to do if you have no replicates
x = assay(siEZH2) group = iEZH2$Stage
y = DGEList(counts=x,group=group)
y = calcNormFactors(y)
y$samples
# group lib.size norm.factors
# Sample1              siEZH2 41941545      0.9808675
# Sample2              siNon 39355314       1.0195057

bcv = 0.01 # recomended by edgeR manual
et = exactTest(y, dispersion=bcv)
write.csv(topTags(et, n=5000, adjust.method="BH",
      sort.by="PValue",p.value=1),
      file = "siEZH2.csv")
```

For siG9a in HCT116 cell line, the procedure was:

```
load(file="siG9a.rda")
x = assay(siG9a) group = iEG9a$Stage
y = GEList(counts=x,group=group)
```

```
y = calcNormFactors(y)
y$samples
# group lib.size norm.factors
#Sample1 siG9a 33123918 1.0065409
#Sample2 siNon 39355314 0.9935016
bcv = 0.01
et = exactTest(y,dispersion=bcv)
write.csv(topTags(et, n=5000, adjust.method="BH",
                  sort.by="PValue", p.value=1),
                  file = "siG9a.csv")
```

3.2. Bowtie2-edgeR

For siEZH2 in HCT116 cell lines, the procedure was:

```
load(file="siEZH2_Bowtie.rda")
x = assay(siEZH2_Bowtie) group = siEZH2_Bowtie$Stage
y = DGEList(counts=x,group=group)
y = calcNormFactors(y)
y$samples
#     group lib.size norm.factors
# Sample1 siEZH2 275877230.9850405
# Sample2siNon 260485281.0151867
bcv = 0.01
et = exactTest(y, dispersion=bcv)
write.csv(topTags(et, n=5000, adjust.method="BH",
                        sort.by="PValue", p.value=1),
                        file = "siEZH2_Bowtie.csv")
```

For siG9a in HCT116 cell line, the procedure was:

```
load(file="siG9a_Bowtie.rda")
x = assay(siG9a_Bowtie) group = siG9a_Bowtie$Stage
y = DGEList(counts=x,group=group)
y = calcNormFactors(y)
y$samples
#     group lib.size norm.factors
# Sample1 siG9a 21498497       1.0149663
# Sample2  siNon 26048528      0.9852544
bcv = 0.01
et = exactTest(y, dispersion=bcv)
write.csv(topTags(et, n=5000, adjust.method="BH",
                        sort.by="PValue", p.value=1),
                  file = "siG9a_Bowtie.csv")
```

4. Contrasting the *in silico* results with those experimentally obtained in HCT116 cells after knocking-down *ZNF518B*

```
Lista siGenes = read.csv(file= "lista_genes.csv",
                        header = TRUE,sep=",")
```

```r
area1 = intersect(Lista_siGenes$siEZH2,Lista_siGenes$siEZH2) length(area1)
# 433

area2 = intersect(Lista_siGenes$siG9A,Lista_siGenes$siG9A) length(area2)
# 190

area3 = intersect(Lista_siGenes$siZNF518B,Lista_siGenes$siZNF518B) length(area3)
# 580

n12 = intersect(area1, area2)
length(n12)
# 90
n23 = intersect(area2, area3)
length(n23)
# 17
n13 = intersect(area1, area3)
length(n13)
# 41
n123 = intersect(intersect(area1, area2), area3)
length(n123)
# 9

library(VennDiagram)

## Loading required package: grid

## Loading required package: futile.logger

draw.triple.venn(area1= 433, area2= 190, area3= 580,
                 n12= 90, n23= 17, n13= 41, n123= 9,
                 category = c("HCT116 siEZH2","HCT116 siEHMT2",
                              "HCT116 siZNF518B"),
                 lty = "blank", sep.dist =1,
                 fill = c("red", "green", "Blue"), euler.d = TRUE
                 scaled = TRUE, rotation.degree = 10, cex = 1, cat.cex = 1,
                 cat.dist = 0.1)
```

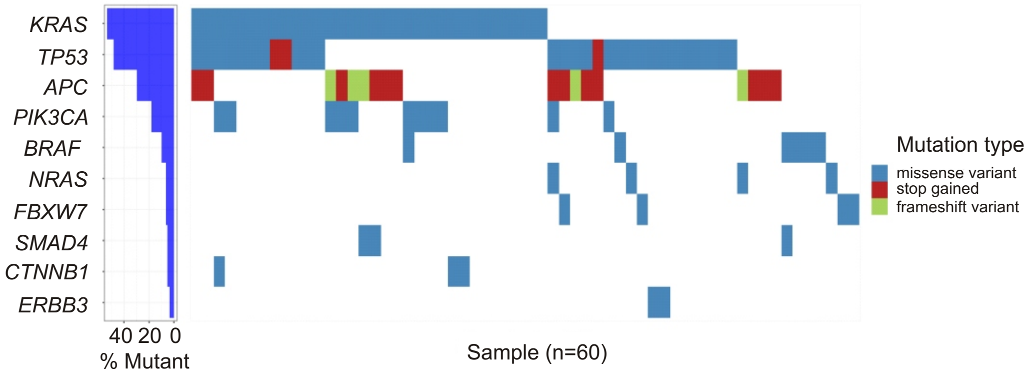

Figure S1. Next-Generation Sequencing for somatic mutations in 60 out of the 66 patients cohort. The percent occurrence and the nature of the different mutations are indicated.

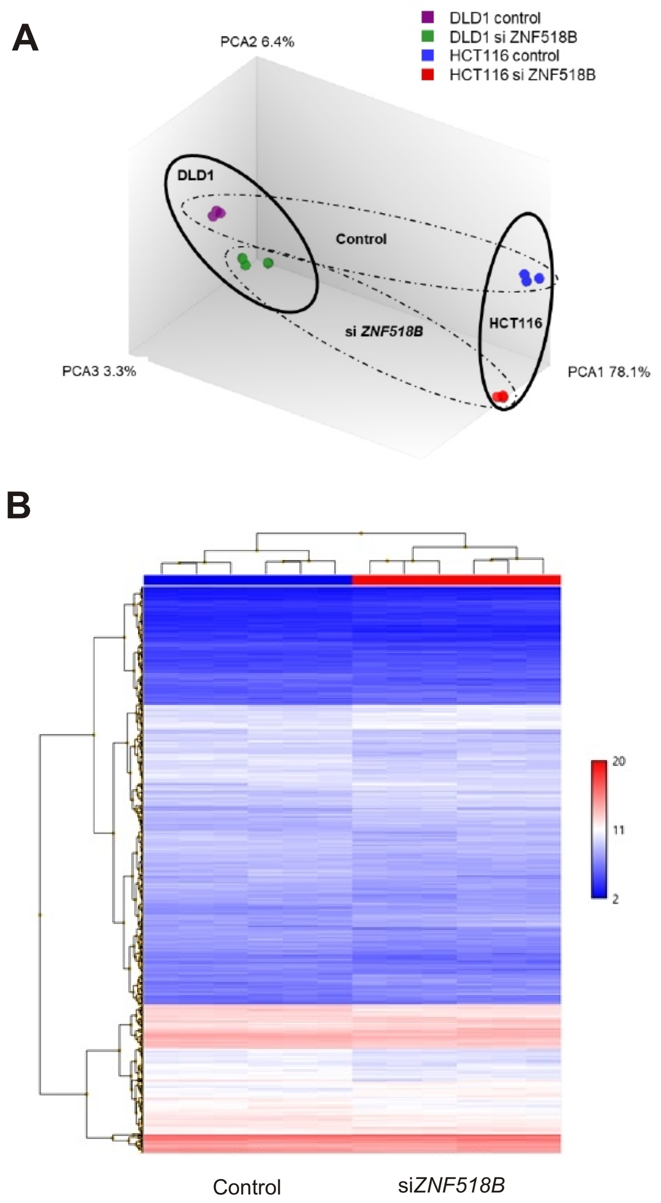

Figure S2. Global analysis of the transcriptomic profile of normal DLD1 and HCT116 cell lines and of cells with silenced *ZNF518B*. The principal component analysis (A) and the heat map (B) are shown.

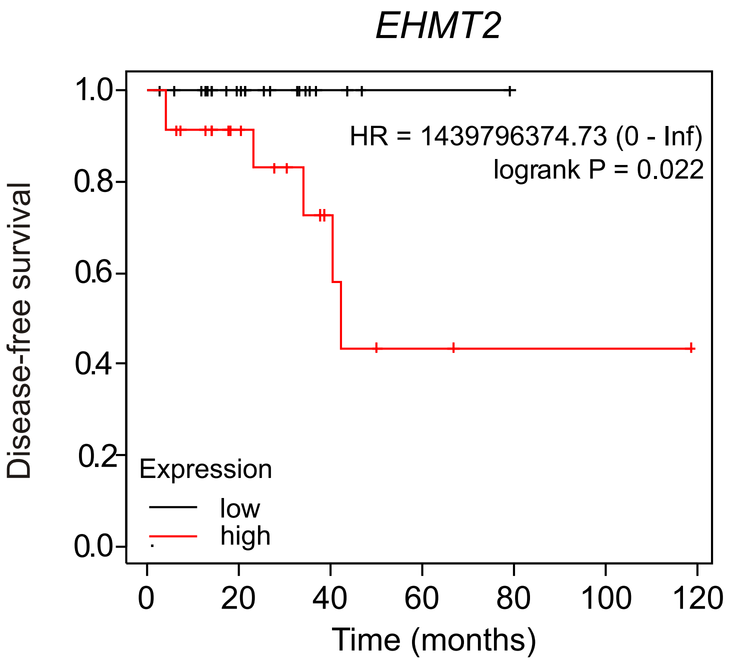

Figure S3. Influence of the level of *EHMT2* transcription on the disease-free survival of CRC patients. The Kaplan-Meier curve was obtained as described in Materials and Methods, with the data retrieved from TCGA database

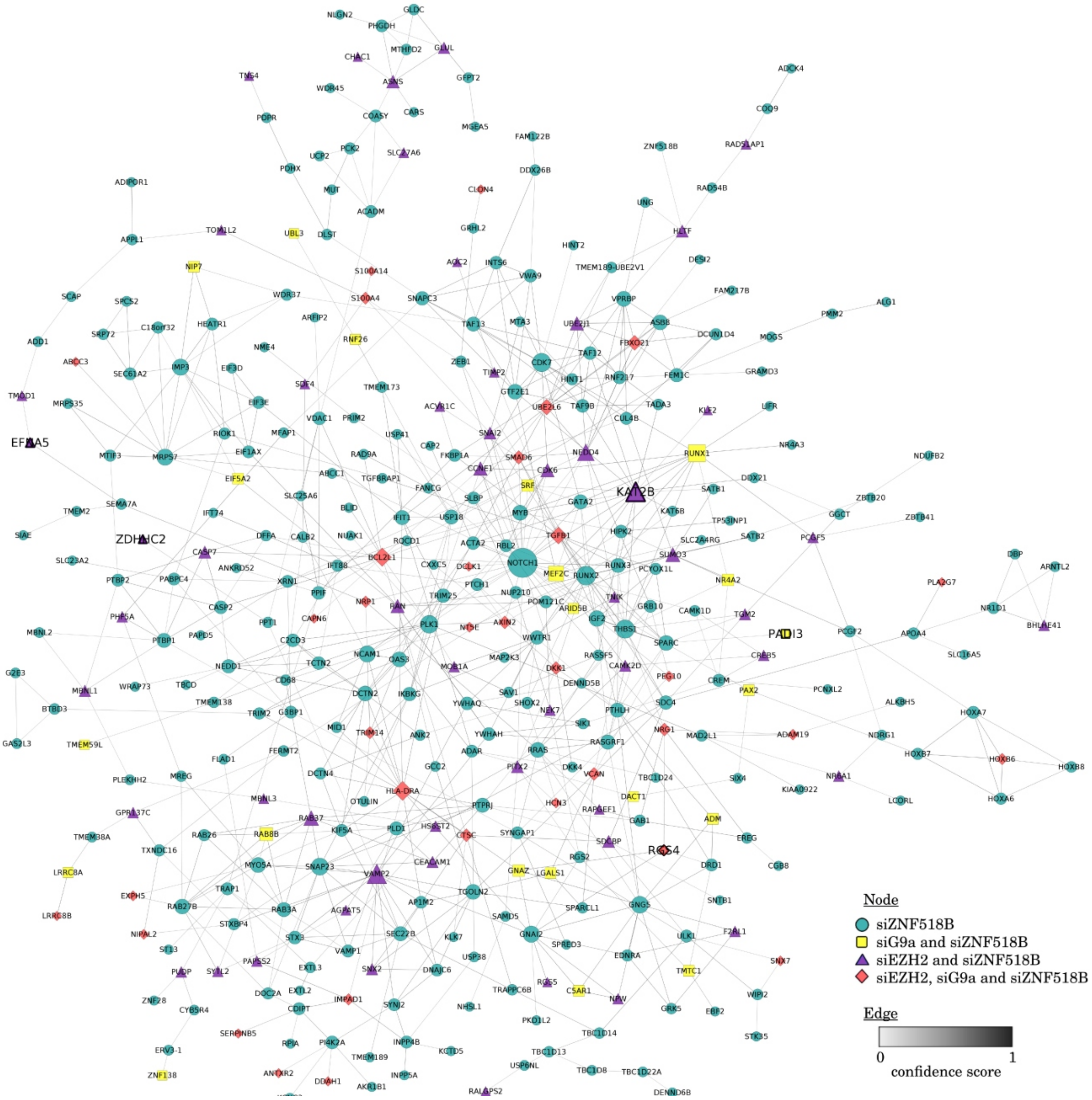

Figure S4. Protein-protein interaction network for genes affected by *ZNF518B*, *EZH2* and *EHMT2*. The network includes 370 nodes and 739 interactions. The size of the nodes is proportional to the number of connections. Nodes labelled with large lettering indicate the five genes analysed by ChIP. The shading intensity of the edges indicates confidence score interaction from 0 to 1.

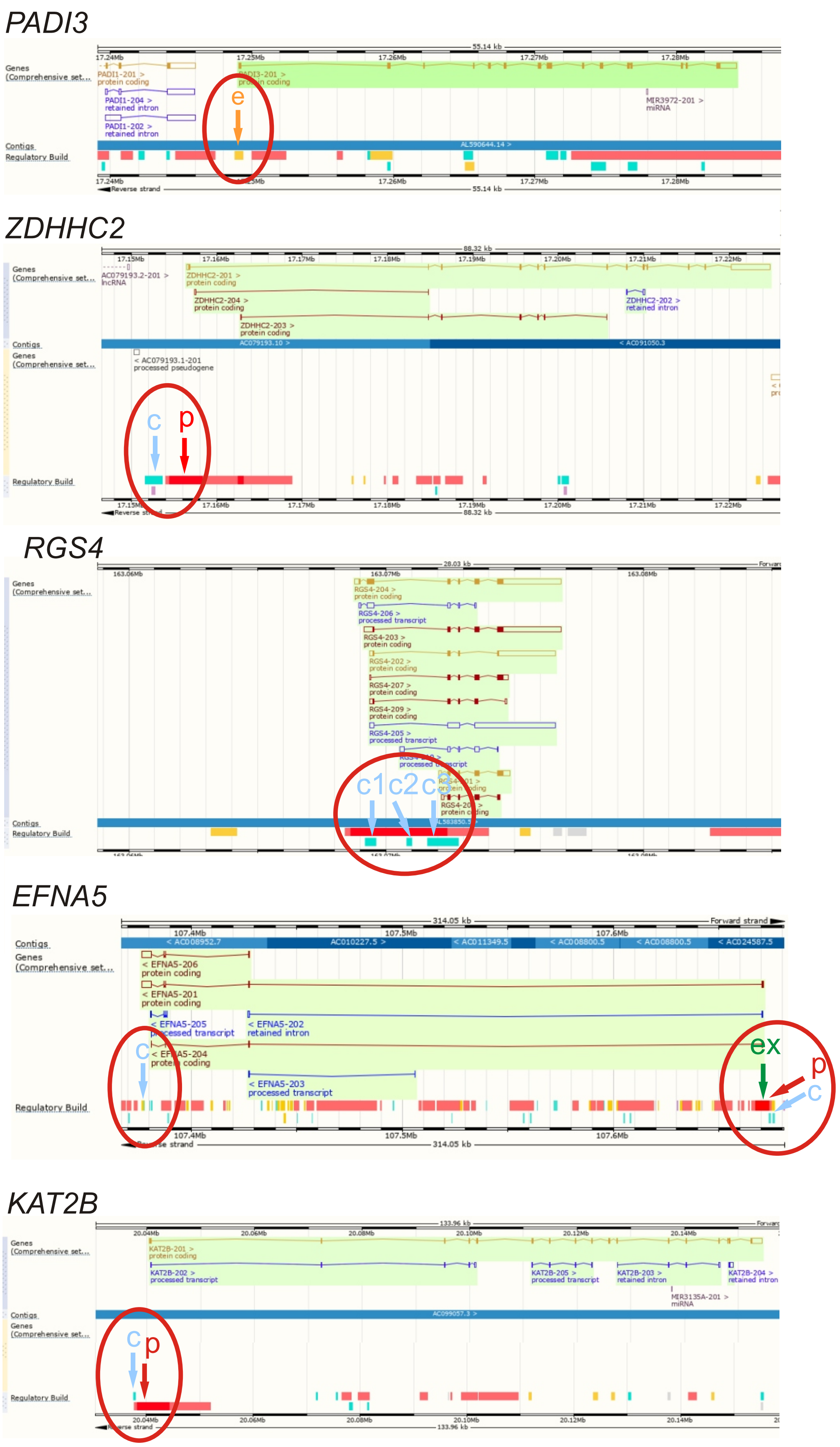

Figure S5. Genomic regions selected for ChIP analysis. Gene map displays were downloaded from Ensembl database (www.ensembl.org) and the amplicons used for qPCR analysis of ChIP data were selected as indicated in Supplementary Methods. The location of amplicons is given by coloured arrows, encircled by red ovals and labelled as p (promoters), e (enhancers), c (CTCF control sequences), ex (exons). Note that *EFNA5* is transcribed from the reverse strand.

Supplementary Table S1. Clinicopathological characteristics of the patients' cohort (n=66)

	**Patients' characteristics**	
		N (%)
T stage	1	1 (1.5)
	2	5 (7.6)
	3	49 (74.2)
	4	11 (16.7)
N stage	Yes	27 (40.9)
	No	39 (59.1)
AJCC Stage	I	4 (6.1)
	II	35 (53.0)
	III	27 (40.9)
Vascular invasion	Yes	21 (31.8)
	No	45 (68.2)
Perineural invasion	Yes	12 (18.2)
	No	54 (81.8)
Relapse	Yes	10 (15.2)
	No	56 (84.8)
CMS stage	1	15 (22.7)
	2	13 (19.7)
	3	8 (12.1)
	4	25 (37.9)
	ND	5 (7.6)

ND: non-determined.

Supplementary Table S2. Genes affected in DLD1 cells by knocking-down the *ZNF518B* gene, as recovered from the Clariom-S assay

ID	Gene Symbol	LogFC	P-value	FDR
TC0X00009007.hg.1	*PUDP*	2.22	3.46E-07	0.0003
TC2000009204.hg.1	*TOMM34*	2.05	6.55E-09	4.68E-05
TC0100007747.hg.1	*GJB3*	1.9	1.47E-06	0.0005
TC0600008760.hg.1	*PM20D2*	1.85	5.85E-07	0.0003
TC0900012024.hg.1	*CLIC3*	1.83	1.22E-07	0.0002
TC0900007576.hg.1	*ANXA1*	1.75	4.22E-09	4.68E-05
TC1200009800.hg.1	*SLC2A3*	1.64	2.37E-07	0.0003
TC0400010048.hg.1	*ZNF518B*	1.62	3.52E-07	0.0003
TC1200010788.hg.1	*SPRYD3*	1.6	4.97E-07	0.0003
TC0600011448.hg.1	*LY6G6C*	1.6	3.44E-06	0.0008
TC0200006687.hg.1	*HPCAL1*	1.59	8.40E-05	0.0049
TC0100012278.hg.1	*SCCPDH*	1.59	1.51E-06	0.0005
TC2000009218.hg.1	*SDC4*	1.57	1.93E-08	9.19E-05
TC1100008037.hg.1	*OVOL1*	1.56	2.60E-06	0.0007
TC1700007102.hg.1	*ALKBH5*	1.49	6.89E-08	0.0001
TC0200011093.hg.1	*ALPP*	1.48	1.04E-06	0.0004
TC1200009796.hg.1	*SLC2A14*	1.46	1.61E-06	0.0005
TC0600011134.hg.1	*HIST1H4D*	1.45	1.27E-05	0.0017
TC2200009257.hg.1	*TCN2*	1.45	3.01E-05	0.0029
TC1700006464.hg.1	*FAM57A*	1.44	1.43E-06	0.0005
TC1000008891.hg.1	*DUSP5*	1.43	2.97E-07	0.0003
TC1200008591.hg.1	*ACTR6*	1.43	7.64E-06	0.0012
TC0500012388.hg.1	*DPYSL3*	1.41	0.0043	0.0548
TC1600007448.hg.1	*CORO1A*	1.4	0.0001	0.0060
TC1700011808.hg.1	*ST6GALNAC2*	1.4	0.0007	0.0175
TC0600011234.hg.1	*HIST1H4L*	1.39	3.27E-06	0.0008
TC1600010409.hg.1	*BBS2*	1.39	4.87E-05	0.0036
TC0700008430.hg.1	*SDHAF3*	1.38	0.0003	0.0095
TC1900011382.hg.1	*MYADM*	1.37	4.35E-06	0.0009
TC0100011533.hg.1	*ATF3*	1.36	5.98E-07	0.0003
TC1600011378.hg.1	*MVP; PAGR1*	1.36	9.45E-07	0.0004
TC1400009248.hg.1	*DLGAP5*	1.36	3.28E-06	0.0008
TC0600007613.hg.1	*HSPA1A; HSPA1B*	1.34	2.52E-07	0.0003
TC0100015336.hg.1	*BCL2L15*	1.34	4.32E-06	0.0009
TC0X00007744.hg.1	*SH3BGRL*	1.34	6.18E-07	0.0003
TC1700008897.hg.1	*MRPS7*	1.31	0.0006	0.0161
TC0200011107.hg.1	*EFHD1*	1.31	0.0011	0.0238
TC0600007311.hg.1	*HMGN4*	1.3	5.13E-07	0.0003
TC2000009392.hg.1	*B4GALT5*	1.29	4.12E-06	0.0009
TC1800009222.hg.1	*RNF125*	1.29	6.54E-05	0.0043
TC0500012166.hg.1	*CDC25C*	1.29	8.61E-06	0.0013
TC0900009930.hg.1	*FANCG*	1.29	8.38E-05	0.0049

TC1100009248.hg.1	*RNF26*	1.28	1.05E-06	0.0004
TC0600011130.hg.1	*HIST1H4C*	1.28	0.0017	0.0310
TC1500007062.hg.1	*CKMT1B; CKMT1A*	1.27	5.34E-07	0.0003
TC0X00008833.hg.1	*FAM50A*	1.27	5.28E-05	0.0038
TC1500007034.hg.1	*SNAP23*	1.26	4.01E-06	0.0009
TC1100006796.hg.1	*AKIP1*	1.26	2.03E-05	0.0023
TC1700008882.hg.1	*KCTD2*	1.25	2.14E-06	0.0006
TC0200015214.hg.1	*OSGEPL1*	1.25	0.0003	0.0108
TC1000008798.hg.1	*SFR1*	1.24	1.60E-05	0.0019
TC0100013483.hg.1	*TAF12*	1.23	8.41E-06	0.0013
TC1600011115.hg.1	*EMC8*	1.22	0.0003	0.0095
TC0100014776.hg.1	*DDAH1*	1.22	4.71E-05	0.0036
TC0100006982.hg.1	*EFHD2*	1.22	3.38E-05	0.0030
TC0500012486.hg.1	*DCTN4*	1.21	4.25E-06	0.0009
TC0400012251.hg.1	*C4orf46*	1.2	2.47E-06	0.0007
TC0500012842.hg.1	*DUSP1*	1.2	0.0002	0.0071
TC1600010732.hg.1	*NQO1*	1.19	1.11E-06	0.0004
TC1900011470.hg.1	*UBE2S*	1.19	1.90E-05	0.0022
TC0100008101.hg.1	*KIF2C*	1.19	0.0001	0.0067
TC0200015127.hg.1	*PDE1A*	1.19	0.0002	0.0073
TC2100008297.hg.1	*SIK1*	1.19	6.13E-06	0.0011
TSUnmapped00000400.hg.1	*SERTAD4*	1.18	0.0001	0.0057
TC1700011892.hg.1	*TK1*	1.18	1.15E-06	0.0004
TC0500007966.hg.1	*XRCC4*	1.18	2.95E-05	0.0028
TC0100013293.hg.1	*ID3*	1.18	0.0001	0.0060
TC1200008675.hg.1	*TXNRD1*	1.18	4.38E-06	0.0009
TC1200007051.hg.1	*PYROXD1*	1.18	2.37E-06	0.0006
TC0100009723.hg.1	*FAM72D*	1.18	0.0010	0.0225
TC1400007706.hg.1	*FOS*	1.17	2.35E-05	0.0026
TC1000007990.hg.1	*DDIT4*	1.17	0.0002	0.0085
TC0200016366.hg.1	*THAP4*	1.17	0.0002	0.0092
TC0700006735.hg.1	*SCIN*	1.17	0.0006	0.0161
TC0X00009386.hg.1	*SRPX*	1.16	5.90E-05	0.0041
TC2100008252.hg.1	*TMPRSS3*	1.16	0.0007	0.0175
TC1500009130.hg.1	*RHOV*	1.16	0.0128	0.1059
TC1100009450.hg.1	*DCPS*	1.15	0.0006	0.0159
TC1600007806.hg.1	*HEATR3*	1.15	0.0001	0.0057
TC0100018186.hg.1	*NBL1*	1.14	0.0001	0.0063
TC0500006730.hg.1	*CCT5*	1.14	2.12E-06	0.0006
TC0200009687.hg.1	*ARL6IP6*	1.13	0.0002	0.0077
TC1600006976.hg.1	*ERCC4*	1.13	4.88E-05	0.0036
TC0900011432.hg.1	*RBM18*	1.13	7.52E-05	0.0047
TC1700009731.hg.1	*GAS7*	1.13	0.0001	0.0053
TC0600007263.hg.1	*HIST1H4A*	1.13	0.0013	0.0274
TC1100009817.hg.1	*PHLDA2*	1.12	8.22E-07	0.0004
TC0700011710.hg.1	*SLC25A40*	1.12	5.27E-06	0.0010

TC0600007616.hg.1	*HSPA1B; HSPA1A*	1.12	1.02E-05	0.0014
TC0X00009124.hg.1	*PIGA*	1.12	1.58E-05	0.0019
TC0200009065.hg.1	*DBI*	1.12	2.75E-06	0.0007
TC0X00009486.hg.1	*MAOB*	1.12	5.55E-05	0.0039
TC1200011567.hg.1	*TMCC3; MIR7844*	1.11	0.0013	0.0264
TC0100017216.hg.1	*NEK2*	1.11	1.74E-05	0.0020
TC0800009856.hg.1	*EGR3*	1.11	0.0008	0.0191
TC0300012145.hg.1	*FSTL1; MIR198*	1.11	0.0033	0.0465
TC1400010730.hg.1	*EGLN3*	1.11	0.0001	0.0068
TC1100008652.hg.1	*TMEM126A*	1.11	0.0026	0.0402
TC1000007272.hg.1	*CREM*	1.1	0.0241	0.1540
TC0600013433.hg.1	*SF3B5*	1.09	0.0004	0.0116
TC1100012667.hg.1	*OR8B3*	1.09	8.50E-05	0.0049
TC0100015332.hg.1	*RSBN1*	1.08	3.07E-05	0.0029
TC2200006540.hg.1	*USP18*	1.08	4.70E-05	0.0036
TC2000009884.hg.1	*AP5S1*	1.08	0.0064	0.0693
TC1600007887.hg.1	*RBL2*	1.07	8.86E-05	0.0050
TC0600006524.hg.1	*FOXQ1*	1.07	2.54E-05	0.0026
TC1200010264.hg.1	*IPO8*	1.07	3.15E-05	0.0029
TC0500008777.hg.1	*KIF20A*	1.07	3.26E-05	0.0030
TC1900009204.hg.1	*MOB3A*	1.07	3.79E-05	0.0031
TC1700010647.hg.1	*KRTAP3-1*	1.07	4.52E-05	0.0035
TC1600009063.hg.1	*MRPS34*	1.07	5.07E-05	0.0037
TC0500009490.hg.1	*BNIP1*	1.07	0.0002	0.0090
TC1200007653.hg.1	*NR4A1*	1.07	0.0002	0.0092
TC0500011984.hg.1	*HINT1*	1.06	3.30E-05	0.0030
TC0200008262.hg.1	*RNF181*	1.06	6.13E-05	0.0042
TC1000008482.hg.1	*CEP55*	1.06	0.0010	0.0232
TC0100010152.hg.1	*LMNA*	1.06	1.29E-05	0.0017
TC1300007592.hg.1	*NDFIP2*	1.06	3.11E-05	0.0029
TC0700006618.hg.1	*WIPI2*	1.06	4.09E-05	0.0033
TC0X00009018.hg.1	*PNPLA4*	1.06	8.14E-05	0.0049
TC0800011132.hg.1	*GEM*	1.06	8.50E-05	0.0049
TC2000007830.hg.1	*TFAP2C*	1.06	0.0001	0.0065
TC0900012189.hg.1	*TMEM141*	1.06	0.0005	0.0148
TC0100017073.hg.1	*SLC45A3*	1.06	0.0031	0.0444
TC0600011138.hg.1	*HIST1H1D*	1.06	0.0041	0.0530
TC1100011338.hg.1	*CDK2AP2*	1.05	2.58E-05	0.0026
TC0100018233.hg.1	*FPGT*	1.05	3.01E-05	0.0029
TC1200007147.hg.1	*ARNTL2*	1.05	3.88E-05	0.0032
TC1900008689.hg.1	*PPP2R1A*	1.05	0.0002	0.0079
TC0900011655.hg.1	*ZER1*	1.05	6.75E-05	0.0044
TC0500012039.hg.1	*ZCCHC10*	1.05	0.0008	0.0196
TC0X00006799.hg.1	*SAT1*	1.05	4.75E-06	0.0010
TC0100017172.hg.1	*IRF6*	1.05	1.00E-05	0.0014
TC2000007083.hg.1	*ID1*	1.05	4.46E-05	0.0035

TC0600011349.hg.1	*RNF39*	1.04	0.0093	0.0853
TC0600007377.hg.1	*HIST1H2BM*	1.04	2.38E-05	0.0026
TC1200011543.hg.1	*PGAM1*	1.04	9.83E-05	0.0053
TC0800007362.hg.1	*PLEKHA2*	1.04	0.0004	0.0129
TC0100015872.hg.1	*S100A14*	1.04	0.0005	0.0148
TC1200006874.hg.1	*BORCS5*	1.04	0.0014	0.0279
TC1500007174.hg.1	*EID1*	1.04	1.75E-05	0.0020
TC0200013257.hg.1	*TGOLN2*	1.04	0.0002	0.0084
TC2200008425.hg.1	*LIF*	1.04	0.0483	0.2293
TC1500007067.hg.1	*CKMT1A*	1.04	0.0005	0.0136
TC0X00009650.hg.1	*WDR45; PRAF2*	1.03	0.0013	0.0264
TC0300014064.hg.1	*PFN2*	1.03	3.64E-05	0.0030
TC0400009525.hg.1	*ANKRD37*	1.03	0.0006	0.0169
TC0700012812.hg.1	*MKRN1*	1.03	8.10E-06	0.0013
TC0700013362.hg.1	*MRPS17*	1.02	0.0078	0.0773
TC1100007003.hg.1	*LDHA*	1.02	5.64E-06	0.0011
TC0200010801.hg.1	*RQCD1*	1.02	1.64E-05	0.0019
TC1700012189.hg.1	*SENP3*	1.02	8.78E-05	0.0050
TC0100015943.hg.1	*DPM3*	1.02	0.0008	0.0207
TC1700009219.hg.1	*RAC3*	1.02	0.0035	0.0480
TC0300013835.hg.1	*TEX264*	1.01	0.0028	0.0426
TC0800007766.hg.1	*RAB2A*	1.01	9.38E-06	0.0014
TC1700009394.hg.1	*PRPF8*	1.01	3.56E-05	0.0030
TC0500007911.hg.1	*SPZ1*	1.01	0.0036	0.0488
TC0600011135.hg.1	*IIST1H3D; HIST1H2AI*	1.01	0.0002	0.0082
TC0100013674.hg.1	*DLGAP3*	1.01	0.0015	0.0289
TC1200011251.hg.1	*TSPAN8*	1	1.00E-05	0.0014
TC0100011397.hg.1	*C4BPB*	1	0.0003	0.0109
TC1200006649.hg.1	*TPI1*	0.99	9.49E-05	0.0052
TC1700012361.hg.1	*CDRT1*	-1	0.0003	0.0107
TC0600007241.hg.1	*LRRC16A*	-1	0.0114	0.0984
TC1100011184.hg.1	*SF1*	-1	0.0004	0.0120
TC0100015947.hg.1	*THBS3*	-1.01	2.95E-05	0.0028
TC1000006796.hg.1	*SEC61A2*	-1.01	3.41E-05	0.0030
TSUnmapped00000401.hg.1	*INPP5D*	-1.01	0.0005	0.0147
TC1700010700.hg.1	*DNAJC7*	-1.01	0.0013	0.0269
TC0800006975.hg.1	*BMP1*	-1.01	0.0003	0.0110
TSUnmapped00000106.hg.1	*LRP6*	-1.01	0.0010	0.0231
TC1200007809.hg.1	*GDF11*	-1.01	0.0017	0.0315
TC0200010253.hg.1	*ANKAR*	-1.01	5.13E-05	0.0037
TC1100011742.hg.1	*KCTD21*	-1.02	4.37E-05	0.0034
TC0600007158.hg.1	*SOX4*	-1.02	2.30E-05	0.0025
TC0100009646.hg.1	*NBPF26*	-1.02	0.0003	0.0110
TC1200006454.hg.1	*WNK1*	-1.02	0.0005	0.0138
TC1100008330.hg.1	*IL18BP*	-1.02	4.00E-06	0.0009
TC2000006736.hg.1	*SPTLC3*	-1.02	3.73E-05	0.0031

TC2200007493.hg.1	*MEI1*	-1.02	6.08E-05	0.0041
TC1800006897.hg.1	*LAMA3*	-1.03	3.31E-05	0.0030
TC0100009101.hg.1	*ABCD3*	-1.03	0.0159	0.1209
TC0900006583.hg.1	*UHRF2*	-1.03	0.0002	0.0074
TC0300011364.hg.1	*CADPS*	-1.03	0.0006	0.0161
TC0200009967.hg.1	*DLX1*	-1.03	0.0270	0.1636
TC0X00007573.hg.1	*OGT*	-1.03	1.29E-05	0.0017
TC0600011173.hg.1	*GUSBP2*	-1.03	1.81E-05	0.0021
TC2000008516.hg.1	*SNX5*	-1.03	0.0109	0.0956
TC0300007117.hg.1	*ENTPD3*	-1.04	0.0004	0.0136
TC1900007543.hg.1	*ZNF257*	-1.04	0.0015	0.0287
TC1100009667.hg.1	*ANO9*	-1.04	9.09E-05	0.0051
TC0200008803.hg.1	*GCC2*	-1.04	0.0007	0.0171
TC1700011919.hg.1	*CEP295NL; TIMP2*	-1.04	2.68E-06	0.0007
TC1700007319.hg.1	*WSB1*	-1.04	1.28E-05	0.0017
TC0500009061.hg.1	*PCYOX1L*	-1.04	2.76E-05	0.0027
TC0500009423.hg.1	*NPM1*	-1.04	8.46E-05	0.0049
TC0200015893.hg.1	*DOCK10*	-1.04	0.0003	0.0108
TSUnmapped00000725.hg.1	*CCDC84*	-1.04	0.0004	0.0123
TC0800011566.hg.1	*RAD21*	-1.04	0.0061	0.0680
TC0500013247.hg.1	*PCDHB14*	-1.04	0.0072	0.0742
TC1700010314.hg.1	*EVI2A; EVI2B*	-1.04	0.0323	0.1817
TC0700011554.hg.1	*PMS2P3*	-1.05	1.05E-06	0.0004
TC0400012854.hg.1	*TMEM144*	-1.05	0.0004	0.0122
TC0600013126.hg.1	*PTPRK*	-1.05	6.05E-06	0.0011
TC0200016319.hg.1	*ANKMY1*	-1.05	1.43E-05	0.0018
TC0600014256.hg.1	*GABBR1*	-1.05	3.32E-05	0.0030
TC1200010839.hg.1	*ITGA5*	-1.05	1.05E-05	0.0014
TC0700009065.hg.1	*CCDC136*	-1.05	0.0001	0.0059
TC0200008462.hg.1	*PROM2*	-1.05	0.0010	0.0234
TC0200007200.hg.1	*LTBP1*	-1.06	2.96E-05	0.0028
TC0600011823.hg.1	*TRERF1*	-1.06	3.42E-05	0.0030
TC1100007675.hg.1	*RTN4RL2*	-1.06	5.50E-05	0.0039
TC0200007908.hg.1	*ANTXR1*	-1.06	0.0008	0.0190
TC0400008183.hg.1	*BMPR1B*	-1.06	0.0021	0.0358
TC1800008750.hg.1	*TCF4*	-1.06	0.0086	0.0822
TC1200012758.hg.1	*TAS2R14*	-1.06	0.0186	0.1345
TC1200007535.hg.1	*CACNB3*	-1.06	2.68E-05	0.0027
TC0600009256.hg.1	*DCBLD1*	-1.06	0.0006	0.0156
TC1400009579.hg.1	*ADAM20*	-1.06	0.0018	0.0325
TC1600011355.hg.1	*NPIPA1*	-1.07	9.52E-06	0.0014
TC1400007478.hg.1	*GPHN*	-1.07	2.24E-05	0.0025
TC0200009470.hg.1	*HNMT*	-1.07	0.0004	0.0117
TC1900009808.hg.1	*ADGRL1*	-1.07	6.40E-05	0.0042
TC1600011354.hg.1	*NPIPA2*	-1.07	2.76E-05	0.0027
TC1100009994.hg.1	*OR2D2*	-1.08	8.45E-05	0.0049

TC2200009161.hg.1	*LMF2*	-1.08	0.0007	0.0174
TC1100012722.hg.1	*CDON*	-1.08	0.0143	0.1134
TC1100007430.hg.1	*GYLTL1B*	-1.08	2.56E-06	0.0007
TC1600011353.hg.1	*NPIPA3*	-1.08	3.31E-05	0.0030
TC1300008668.hg.1	*LHFP*	-1.08	3.64E-05	0.0030
TC0300012048.hg.1	*ZBTB20; MIR568*	-1.08	4.42E-05	0.0035
TC0400012933.hg.1	*NAAA*	-1.09	1.16E-06	0.0004
TC0100012695.hg.1	*DNAJC11*	-1.09	0.0266	0.1628
TC1600011487.hg.1	*NPIPA8*	-1.09	9.67E-06	0.0014
TC1900010037.hg.1	*SUGP2*	-1.09	2.45E-05	0.0026
TC0700010989.hg.1	*TNS3*	-1.09	3.46E-05	0.0030
TC0200007421.hg.1	*CAMKMT*	-1.09	9.65E-05	0.0052
TC0700011571.hg.1	*YWHAG*	-1.09	0.0006	0.0168
TC0300013601.hg.1	*ATP13A3*	-1.09	0.0009	0.0218
TC0300007474.hg.1	*DNAH1*	-1.09	0.0010	0.0225
TC0100014459.hg.1	*LINC01359*	-1.09	0.0006	0.0170
TC0300007051.hg.1	*ITGA9*	-1.1	2.31E-06	0.0006
TSUnmapped00000267.hg.1	*LRP6*	-1.1	5.68E-06	0.0011
TC1700008095.hg.1	*LRRC37A*	-1.1	1.98E-05	0.0022
TC0300007647.hg.1	*PTPRG*	-1.1	0.0005	0.0152
TC0100011091.hg.1	*C1orf53*	-1.1	0.0009	0.0208
TC0200016648.hg.1	*CDC42EP3*	-1.1	2.68E-05	0.0027
TC1300007197.hg.1	*DLEU1*	-1.1	0.0002	0.0072
TC0200010511.hg.1	*NBEAL1*	-1.1	0.0010	0.0223
TC0200008627.hg.1	*NPAS2*	-1.11	4.77E-06	0.0010
TC0700012755.hg.1	*KIAA1549*	-1.11	6.62E-06	0.0011
TC1200006786.hg.1	*TMEM52B*	-1.11	0.0001	0.0063
TC1900011190.hg.1	*IZUMO2*	-1.11	0.0032	0.0458
TC0500013231.hg.1	*CATSPER3*	-1.11	0.0078	0.0772
TC0100016135.hg.1	*SLAMF6*	-1.12	0.0002	0.0090
TC0600009697.hg.1	*PHACTR2*	-1.12	0.0022	0.0368
TC0100013630.hg.1	*YARS*	-1.12	0.0029	0.0429
TC1600009524.hg.1	*NPIPA5*	-1.12	5.98E-06	0.0011
TC0200011195.hg.1	*GAP1; noygloy; torkeyl*	-1.12	0.0002	0.0078
TC1900011796.hg.1	*NDUFA3*	-1.12	0.0002	0.0081
TC0700013401.hg.1	*AC007566.10; GATAD*	-1.12	0.0004	0.0123
TC1000007954.hg.1	*SLC29A3*	-1.12	0.0006	0.0157
TC1200007804.hg.1	*METTL7B*	-1.12	0.0012	0.0253
TC0800010945.hg.1	*SLC10A5*	-1.12	0.0021	0.0357
TC2200009267.hg.1	*GTPBP1*	-1.12	0.0176	0.1291
TC0500012210.hg.1	*TMEM173*	-1.13	1.75E-06	0.0005
TC1100013057.hg.1	*TCIRG1*	-1.13	4.15E-05	0.0033
TC0300013913.hg.1	*TPRG1*	-1.13	0.0124	0.1040
TC0200010729.hg.1	*XRCC5*	-1.13	3.79E-05	0.0031
TC0700007034.hg.1	*CREB5*	-1.13	0.0087	0.0829
TC1500006925.hg.1	*THBS1*	-1.14	0.0003	0.0099

TC1900008188.hg.1	*MEGF8*	-1.14	0.0009	0.0213
TC1100010643.hg.1	*TP53I11*	-1.14	0.0074	0.0754
TC1900011919.hg.1	*ZNF708*	-1.14	5.83E-05	0.0041
TC0900008250.hg.1	*ZNF189*	-1.14	0.0001	0.0068
TC1000012130.hg.1	*FAM53B*	-1.14	0.0146	0.1144
TC0200007194.hg.1	*BIRC6*	-1.15	0.0001	0.0066
TC1600007037.hg.1	*NPIPA7*	-1.15	8.83E-07	0.0004
TC2000009317.hg.1	*SULF2*	-1.15	3.54E-05	0.0030
TC0900012173.hg.1	*GARNL3*	-1.15	5.23E-06	0.0010
TC0100014660.hg.1	*FUBP1*	-1.15	0.0001	0.0066
TC0700013578.hg.1	*SEMA3A*	-1.16	9.69E-05	0.0052
TSUnmapped00000073.hg.1	*NDUFA10*	-1.16	0.0292	0.1715
TC1300008705.hg.1	*ELF1*	-1.17	2.46E-05	0.0026
TC0100008100.hg.1	*C1orf228*	-1.18	0.0006	0.0169
TC0300008316.hg.1	*PVRL3*	-1.18	7.32E-06	0.0012
TC1600011365.hg.1	*NPIPB9*	-1.19	4.81E-06	0.0010
TC1300009522.hg.1	*UGGT2*	-1.19	8.36E-05	0.0049
TC1300008927.hg.1	*KPNA3*	-1.19	0.0006	0.0156
TC0100013897.hg.1	*P3H1*	-1.19	5.64E-07	0.0003
TC1600008646.hg.1	*ATP2C2*	-1.19	8.85E-05	0.0050
TC1600006826.hg.1	*ABAT*	-1.19	0.0061	0.0677
TC1100009233.hg.1	*CCDC84*	-1.19	5.76E-06	0.0011
TC0200007999.hg.1	*ZNF638*	-1.19	2.94E-05	0.0028
TC1600007368.hg.1	*ATP2A1*	-1.2	0.0002	0.0078
TC1100010207.hg.1	*SOX6; MIR6073*	-1.2	0.0004	0.0131
TC0600014074.hg.1	*HIVEP1*	-1.2	8.02E-07	0.0004
TC1800008235.hg.1	*ABHD3*	-1.2	0.0010	0.0233
TC0X00008514.hg.1	*DDX26B*	-1.2	0.0011	0.0236
TC0600014268.hg.1	*PRRT1*	-1.2	8.86E-06	0.0013
TC0X00011338.hg.1	*ARSD*	-1.21	5.00E-06	0.0010
TC1600010740.hg.1	*PDXDC2P*	-1.21	6.73E-06	0.0012
TC1500007238.hg.1	*TMOD2*	-1.21	0.0004	0.0135
TSUnmapped00000154.hg.1	*LRP6*	-1.21	1.82E-06	0.0005
TC1100006485.hg.1	*TMEM80*	-1.21	2.79E-06	0.0007
TC2000008381.hg.1	*JAG1*	-1.21	7.30E-06	0.0012
TC0100013205.hg.1	*ECE1*	-1.21	9.92E-06	0.0014
TC0500013282.hg.1	*ZDHHC11*	-1.21	1.14E-05	0.0015
TC0200009955.hg.1	*CYBRD1*	-1.21	4.51E-05	0.0035
TC1500008616.hg.1	*HERC2P3*	-1.21	0.0001	0.0057
TC0900011501.hg.1	*NR6A1*	-1.21	0.0008	0.0206
TC0700009696.hg.1	*CHPF2; MIR671*	-1.22	0.0014	0.0281
_GL000194v1_random0000643:	*MAFIP; TEKT4P2*	-1.22	0.0063	0.0691
TC0100010121.hg.1	*HCN3*	-1.22	7.38E-06	0.0012
TC1400010390.hg.1	*AHNAK2*	-1.22	3.10E-05	0.0029
TC0800007460.hg.1	*HGSNAT*	-1.23	5.13E-06	0.0010
TC0600010936.hg.1	*ATXN1*	-1.23	4.64E-05	0.0036

TC1600008977.hg.1	*CCDC78*	-1.23	0.0002	0.0070
TC0700009472.hg.1	*EPHB6*	-1.23	5.89E-05	0.0041
TC2100007274.hg.1	*NDUFV3*	-1.23	0.0018	0.0325
TC1700012423.hg.1	*LRRC37A4P*	-1.23	4.73E-05	0.0036
TC0100018280.hg.1	*AM231D; LINC0086*	-1.24	3.60E-06	0.0008
TC1100006495.hg.1	*TSPAN4*	-1.24	3.65E-05	0.0030
TC1200007594.hg.1	*ASIC1*	-1.24	0.0008	0.0194
TC2000009360.hg.1	*PREX1*	-1.24	0.0481	0.2289
TC0400008093.hg.1	*PKD2*	-1.25	1.58E-06	0.0005
TC0200012980.hg.1	*SPRV1; PCBP1-AS1*	-1.25	1.46E-05	0.0018
TC1100006444.hg.1	*ATHL1*	-1.25	8.54E-05	0.0049
TC0700009215.hg.1	*EXOC4*	-1.25	0.0018	0.0327
TC0100016322.hg.1	*GPR161*	-1.26	4.53E-06	0.0009
TC1200009734.hg.1	*VAMP1*	-1.26	2.02E-06	0.0006
TC1700012296.hg.1	*PRKAR1A; ARSG*	-1.27	7.41E-06	0.0012
TC0100007789.hg.1	*ago-03*	-1.27	3.33E-05	0.0030
TC1400010756.hg.1	*GPR135*	-1.27	0.0004	0.0118
TC1600011368.hg.1	*LAT*	-1.27	4.26E-06	0.0009
TC1400010774.hg.1	*FOXN3*	-1.27	6.46E-05	0.0043
TC1900010531.hg.1	*ZNF461*	-1.27	6.77E-05	0.0044
TSUnmapped00000313.hg.1	*CCDC84*	-1.27	9.84E-05	0.0053
TC0100010674.hg.1	*GPR52*	-1.27	0.0360	0.1933
TC0800010285.hg.1	*SLC20A2*	-1.28	8.22E-06	0.0013
TC1600009855.hg.1	*NPIPB6*	-1.28	1.21E-05	0.0016
TC1100009200.hg.1	*CD3E*	-1.28	3.75E-05	0.0031
TC1200009997.hg.1	*GRIN2B*	-1.28	2.19E-05	0.0024
TC0200016544.hg.1	*RIF1*	-1.29	7.28E-05	0.0046
TC0800008146.hg.1	*WWP1*	-1.29	0.0007	0.0183
TC0M00006434.hg.1	*ND2*	-1.3	3.02E-07	0.0003
TC2200007505.hg.1	*sep-03*	-1.3	4.84E-07	0.0003
TC0400008483.hg.1	*UGT8*	-1.3	2.66E-07	0.0003
TC0400012947.hg.1	*GPRIN3*	-1.3	0.0001	0.0057
TC0200012031.hg.1	*HADHA*	-1.31	0.0051	0.0609
TC1000012586.hg.1	*SEC31B*	-1.31	1.62E-06	0.0005
TC0800006873.hg.1	*PDGFRL*	-1.31	0.0004	0.0128
TC0400007542.hg.1	*KIT*	-1.31	0.0025	0.0398
TC0300006465.hg.1	*LRRN1*	-1.31	7.35E-06	0.0012
TC0600014192.hg.1	*P11-307P5.1; SAMD*	-1.31	1.75E-05	0.0020
TC0900010886.hg.1	*PTCH1*	-1.33	9.68E-07	0.0004
TC0900009679.hg.1	*MLLT3*	-1.33	0.0360	0.1935
TC1100008985.hg.1	*ATM*	-1.35	5.22E-06	0.0010
TC0200007261.hg.1	*QPCT*	-1.35	0.0005	0.0154
TC0100011382.hg.1	*DYRK3*	-1.35	0.0254	0.1586
TC0800008943.hg.1	*PHF20L1*	-1.35	6.57E-05	0.0043
TC1700008261.hg.1	*EPN3*	-1.35	0.0117	0.1005
TC1600011465.hg.1	*ERVK13-1*	-1.36	2.99E-06	0.0007

TC0400012903.hg.1	*PROM1*	-1.36	1.49E-05	0.0018
TC1100010688.hg.1	*PHF21A*	-1.36	4.94E-06	0.0010
TC1600011235.hg.1	*GALNS*	-1.36	4.74E-05	0.0036
TC1900011328.hg.1	*ZNF160*	-1.37	0.0005	0.0150
TC0200015894.hg.1	*DOCK10*	-1.37	0.0061	0.0677
TC1200010601.hg.1	*FKBP11; ARF3*	-1.37	1.74E-06	0.0005
TC1900011869.hg.1	*RGL3*	-1.38	2.55E-07	0.0003
TC0200008534.hg.1	*ANKRD36*	-1.38	9.00E-07	0.0004
TC0100015990.hg.1	*PAQR6*	-1.38	1.16E-06	0.0004
TC1900011194.hg.1	*KCNC3*	-1.38	1.24E-06	0.0004
TC0100006486.hg.1	*AGRN*	-1.38	1.11E-05	0.0015
TC0500013322.hg.1	*NAIP*	-1.38	2.68E-05	0.0027
TC1800008287.hg.1	*ANKRD29*	-1.39	0.0006	0.0169
TC1300008114.hg.1	*CUL4A*	-1.4	0.0002	0.0081
TC1700008085.hg.1	*STH*	-1.4	0.0009	0.0213
TC0200013567.hg.1	*ANKRD36B*	-1.41	1.58E-07	0.0002
TSUnmapped00000246.hg.1	*CCDC84*	-1.41	9.57E-07	0.0004
TC0100017713.hg.1	*TTC13*	-1.41	4.79E-08	0.0001
TC0300013513.hg.1	*P3H2*	-1.42	9.24E-05	0.0051
TC1600009916.hg.1	*NPIPB11*	-1.42	7.77E-07	0.0004
TC1600011364.hg.1	*NPIPB5*	-1.42	6.33E-06	0.0011
TC1100009859.hg.1	*NUP98*	-1.42	2.00E-05	0.0023
TC1600007353.hg.1	*NPIPB8*	-1.43	1.03E-05	0.0014
TC2200009170.hg.1	*ARSA*	-1.43	0.0006	0.0158
TC1900007888.hg.1	*APLP1*	-1.43	0.0013	0.0267
TC1900011868.hg.1	*EPOR*	-1.44	1.30E-06	0.0005
TC0100014769.hg.1	*MCOLN3*	-1.44	7.23E-06	0.0012
TC1600007030.hg.1	*PIPA7; NPIPA8; PKD1*	-1.45	1.20E-06	0.0004
TC1600008407.hg.1	*NPIPB15*	-1.45	3.79E-06	0.0009
TC0100015797.hg.1	*POGZ*	-1.45	0.0052	0.0612
TC1000012144.hg.1	*CTBP2*	-1.46	2.96E-06	0.0007
TC1900011398.hg.1	*TMC4*	-1.46	1.42E-05	0.0018
TC1200006670.hg.1	*CLSTN3*	-1.46	5.17E-06	0.0010
TC0100009149.hg.1	*PTBP2*	-1.46	7.50E-05	0.0047
TC1400007774.hg.1	*NRXN3*	-1.47	6.83E-07	0.0004
TC0200016354.hg.1	*HDLBP*	-1.47	0.0024	0.0381
TC0600010066.hg.1	*IGF2R*	-1.49	9.71E-08	0.0002
TC1200010185.hg.1	*ITPR2*	-1.49	5.46E-06	0.0010
TC0200010448.hg.1	*STRADB*	-1.49	0.0004	0.0128
TSUnmapped00000178.hg.1	*SLC16A1*	-1.5	2.67E-05	0.0027
TC0400008972.hg.1	*DCLK2*	-1.51	2.04E-06	0.0006
TC0100010926.hg.1	*OCLM*	-1.51	0.0016	0.0301
TC0300013520.hg.1	*CLDN1*	-1.52	5.18E-05	0.0038
TC0300006696.hg.1	*NR2C2*	-1.52	0.0003	0.0103
TC0600011943.hg.1	*ENPP5*	-1.53	8.02E-08	0.0001
TC0900010056.hg.1	*ANKRD18A; FAM95C*	-1.54	8.04E-06	0.0013

TC0100018526.hg.1	*ILDR2*	-1.55	1.31E-05	0.0017
TC1900008286.hg.1	*BCAM*	-1.55	6.28E-07	0.0003
TC1700008263.hg.1	*ABCC3*	-1.56	7.19E-07	0.0004
TC0100012460.hg.1	*UBE2J2*	-1.56	9.25E-05	0.0051
TC0400010529.hg.1	*APBB2*	-1.56	2.29E-05	0.0025
TC1600009396.hg.1	*TNP2*	-1.56	0.0004	0.0127
TC0200007188.hg.1	*BIRC6*	-1.58	0.0010	0.0223
TC0600014111.hg.1	*SYNGAP1; MIR5004*	-1.59	5.46E-07	0.0003
TC0600007387.hg.1	*OR2B6*	-1.59	8.78E-06	0.0013
TC1600011505.hg.1	*NPIPB4*	-1.6	4.39E-06	0.0009
TC0600009240.hg.1	*KPNA5*	-1.61	0.0001	0.0060
TC1900008012.hg.1	*CATSPERG*	-1.61	8.09E-05	0.0049
TC1700010363.hg.1	*P11-466A19.5; MYO1*	-1.63	3.20E-07	0.0003
TC1100007366.hg.1	*TTC17*	-1.64	1.91E-06	0.0006
TC1700008382.hg.1	*MSI2*	-1.68	0.0002	0.0070
TC1600009958.hg.1	*NPIPB4*	-1.68	1.70E-07	0.0002
TC1200010006.hg.1	*PLBD1*	-1.68	2.14E-06	0.0006
TC0100011621.hg.1	*TGFB2; TGFB2-OT1*	-1.69	4.58E-08	0.0001
TC0100016983.hg.1	*CHI3L1*	-1.69	6.01E-05	0.0041
TC0200008516.hg.1	*CNNM3*	-1.7	0.0002	0.0077
TC0500011334.hg.1	*TMEM167A*	-1.72	0.0248	0.1566
TC1900010782.hg.1	*ATP1A3*	-1.75	2.57E-08	9.19E-05
TC0800007016.hg.1	*CHMP7*	-1.79	0.0001	0.0068
TC1600011501.hg.1	*NPIPB3*	-1.79	1.31E-06	0.0005
TC0400006491.hg.1	*IDUA*	-1.8	3.67E-07	0.0003
TC0600011615.hg.1	*DEF6*	-1.87	3.20E-05	0.0029
TC1000010273.hg.1	*NRP1*	-1.89	9.24E-07	0.0004
TC0200007273.hg.1	*RMDN2*	-1.92	2.36E-07	0.0003
TC1100007453.hg.1	*MDK*	-1.92	5.70E-09	4.68E-05
TC0800011595.hg.1	*EXT1*	-1.95	5.32E-07	0.0003
TC0100009142.hg.1	*PTBP2*	-1.96	2.26E-08	9.19E-05
TC0700011318.hg.1	*ERV3-1; ZNF117*	-1.96	6.92E-07	0.0004
TC0700013443.hg.1	*CFTR*	-1.97	5.66E-08	0.0001
TC0200009443.hg.1	*R3HDM1*	-2.07	1.89E-07	0.0002
TC1500009709.hg.1	*CA12*	-2.43	3.70E-08	0.0001

The fold change was calculated as (expression in control cells)/(expression in cells silenced for *ZNF518B*) and expressed as logarithm in base 2

Supplementary Table S3. Genes affected in HCT116 cells by knocking-down the *ZNF518B* gene, as recovered from the Clariom-S assay

ID	Gene Symbol	LogFC	P-value	FDR
TC2000009204.hg.1	*TOMM34*	3.39	1.38E-12	1.48E-08
TC0X00009386.hg.1	*SRPX*	3.34	1.42E-10	5.02E-07
TC1500010723.hg.1	*CHAC1*	3.32	2.56E-07	5.71E-05
TC0100009621.hg.1	*PHGDH*	2.99	8.47E-09	6.73E-06
TC0X00007744.hg.1	*SH3BGRL*	2.54	1.02E-09	1.56E-06
TC0500012486.hg.1	*DCTN4*	2.45	1.48E-10	5.02E-07
TC0400010048.hg.1	*ZNF518B*	2.44	4.47E-09	4.57E-06
TC0100014776.hg.1	*DDAH1*	2.39	5.74E-08	2.16E-05
TC1200007147.hg.1	*ARNTL2*	2.27	3.36E-08	1.47E-05
TC1100007785.hg.1	*TMEM109*	2.22	6.14E-11	4.39E-07
TC0800011150.hg.1	*TP53INP1*	2.15	6.68E-08	2.39E-05
TC2000007456.hg.1	*TTPAL*	2.13	2.01E-07	4.84E-05
TC0200008071.hg.1	*MTHFD2*	2.09	6.22E-09	5.56E-06
TC0900006708.hg.1	*SNAPC3*	1.98	7.97E-09	6.58E-06
TC1000011370.hg.1	*ACTA2*	1.92	2.65E-09	3.32E-06
TC0X00009650.hg.1	*WDR45; PRAF2*	1.9	9.63E-08	2.99E-05
TC0200009687.hg.1	*ARL6IP6*	1.87	5.39E-07	9.32E-05
TC0900011655.hg.1	*ZER1*	1.86	7.32E-08	2.45E-05
TC0700012684.hg.1	*AKR1B1*	1.85	6.22E-08	2.26E-05
TC0900008028.hg.1	*MFSD14B*	1.83	5.72E-09	5.45E-06
TC1700008342.hg.1	*PCTP*	1.81	1.66E-07	4.22E-05
TC0800007351.hg.1	*TACC1*	1.8	2.93E-08	1.42E-05
TC1700008897.hg.1	*MRPS7*	1.8	2.38E-06	0.0003
TC1700012260.hg.1	*COASY*	1.8	5.83E-10	1.14E-06
TC2000009392.hg.1	*B4GALT5*	1.8	6.56E-09	5.63E-06
TC0100016162.hg.1	*B4GALT3*	1.79	1.36E-07	3.74E-05
TC1700008882.hg.1	*KCTD2*	1.78	2.77E-09	3.32E-06
TC0500012935.hg.1	*KIAA1191*	1.78	1.38E-08	8.55E-06
TC2000009218.hg.1	*SDC4*	1.77	1.01E-09	1.56E-06
TC1500007034.hg.1	*SNAP23*	1.77	5.32E-08	2.07E-05
TC0200007399.hg.1	*PLEKHH2*	1.76	0.0006	0.0123
TC1000008733.hg.1	*MFSD13A*	1.74	2.99E-08	1.42E-05
TC0800007459.hg.1	*POMK*	1.74	5.85E-09	5.45E-06
TC1500008341.hg.1	*MAN2A2*	1.72	4.52E-07	8.22E-05
TC2000010026.hg.1	*TMEM189*	1.71	3.97E-09	4.28E-06
TC0800006980.hg.1	*SLC39A14*	1.71	2.67E-08	1.33E-05
TC0200009065.hg.1	*DBI*	1.7	1.03E-08	7.86E-06
TC0800008416.hg.1	*GRHL2*	1.69	1.17E-06	0.0002
TC0X00010966.hg.1	*ATP11C*	1.69	2.47E-07	5.58E-05
TC0100015866.hg.1	*S100A4*	1.68	3.23E-05	0.0017
TC1200009252.hg.1	*TCTN2*	1.68	5.36E-07	9.32E-05

TC1900006622.hg.1	*NCLN*	1.67	9.91E-07	0.0001
TC0700011876.hg.1	*ASNS*	1.67	0.0003	0.0085
TC0700007993.hg.1	*CLDN4*	1.66	3.99E-09	4.28E-06
TC1100007819.hg.1	*MYRF*	1.64	1.03E-05	0.0007
TC1600010711.hg.1	*CHTF8*	1.63	1.70E-08	9.59E-06
TC0600011697.hg.1	*CCDC167*	1.58	6.17E-07	0.0001
TC0200016419.hg.1	*GPN1*	1.57	4.48E-08	1.89E-05
TC0100013818.hg.1	*PPT1*	1.56	5.41E-08	2.07E-05
TC0900010968.hg.1	*TRIM14*	1.56	1.16E-06	0.0002
TC2200008832.hg.1	*DESI1*	1.55	5.97E-07	9.93E-05
TC1200010902.hg.1	*ANKRD52*	1.55	7.80E-07	0.0001
TC0300010209.hg.1	*TADA3*	1.54	8.01E-07	0.0001
TC1200008792.hg.1	*UNG*	1.54	1.27E-06	0.0002
TC1400010169.hg.1	*SETD3*	1.52	8.42E-08	2.78E-05
TC1500010080.hg.1	*IMP3*	1.52	8.88E-05	0.0034
TC1100011032.hg.1	*CYB561A3*	1.52	3.38E-07	6.77E-05
TC0200016686.hg.1	*TEX261*	1.51	2.33E-06	0.0003
TC0800007738.hg.1	*SDCBP*	1.51	1.18E-08	8.16E-06
TC1500008245.hg.1	*ABHD2*	1.51	1.35E-06	0.0002
TC0900008887.hg.1	*TBC1D13*	1.51	5.88E-07	9.86E-05
TC0100015872.hg.1	*S100A14*	1.5	4.06E-06	0.0004
TC0200008351.hg.1	*RPIA*	1.5	1.48E-07	3.95E-05
TC0100015182.hg.1	*TAF13*	1.5	1.10E-07	3.32E-05
TC0800011211.hg.1	*NIPAL2*	1.48	0.0009	0.0173
TC1200012723.hg.1	*ZNF664*	1.47	3.54E-07	6.84E-05
TC0900009930.hg.1	*FANCG*	1.47	1.59E-05	0.0010
TC2000010027.hg.1	*TMEM189-UBE2V1*	1.47	1.40E-08	8.55E-06
TC0100010525.hg.1	*SFT2D2*	1.47	4.90E-07	8.68E-05
TC0700009488.hg.1	*CASP2*	1.47	1.53E-08	9.09E-06
TC0200010801.hg.1	*RQCD1*	1.46	8.66E-08	2.82E-05
TC0600011808.hg.1	*MED20*	1.46	9.38E-05	0.0035
TC0200011690.hg.1	*YWHAQ*	1.45	3.49E-07	6.84E-05
TC2200009028.hg.1	*CERK*	1.45	3.70E-07	7.02E-05
TC0600011996.hg.1	*MUT*	1.45	0.0007	0.0147
TC0100008620.hg.1	*DNAJC6*	1.45	0.0032	0.0406
TC0200008501.hg.1	*ITPRIPL1*	1.44	2.00E-05	0.0012
TC1100007801.hg.1	*TMEM138*	1.43	5.85E-07	9.86E-05
TC0900011432.hg.1	*RBM18*	1.43	4.63E-06	0.0004
TC0500009481.hg.1	*ATP6V0E1*	1.41	6.31E-05	0.0027
TC1000012491.hg.1	*ENTPD7*	1.41	2.97E-07	6.36E-05
TC0100006619.hg.1	*TPRG1L*	1.41	1.69E-05	0.0010
TC0700011054.hg.1	*GRB10*	1.4	8.52E-07	0.0001
TC0100015079.hg.1	*EXTL2*	1.4	7.24E-05	0.0030
TC0600009534.hg.1	*MYB*	1.4	3.79E-05	0.0019
TC1400007695.hg.1	*EIF2B2*	1.4	1.58E-06	0.0002
TC0600008066.hg.1	*KLHDC3*	1.39	7.29E-07	0.0001

TC2200006540.hg.1	*USP18*	1.39	1.66E-07	4.22E-05
TC0100013483.hg.1	*TAF12*	1.39	1.67E-07	4.22E-05
TC0800012284.hg.1	*EXTL3*	1.38	2.00E-06	0.0002
TC1100009248.hg.1	*RNF26*	1.38	1.49E-07	3.95E-05
TC0100012089.hg.1	*GPR137B*	1.38	5.18E-07	9.10E-05
TC1400006715.hg.1	*PCK2*	1.38	0.0033	0.0413
TC1800009284.hg.1	*C18orf32*	1.37	2.07E-07	4.93E-05
TC1800006635.hg.1	*TWSG1*	1.36	2.54E-08	1.29E-05
TC1000012577.hg.1	*LIPA*	1.36	1.43E-07	3.88E-05
TC1000008400.hg.1	*IFIT1*	1.36	1.26E-06	0.0002
TC2000007457.hg.1	*PKIG*	1.36	0.0002	0.0066
TC1100010051.hg.1	*TMEM9B*	1.36	3.58E-07	6.86E-05
TC1400007691.hg.1	*DLST*	1.35	2.96E-06	0.0003
TC1200006905.hg.1	*FAM234B*	1.35	1.01E-05	0.0007
TC1700008328.hg.1	*STXBP4*	1.35	6.09E-05	0.0027
TC1800007543.hg.1	*SERPINB5*	1.35	0.0001	0.0037
TC0400012251.hg.1	*C4orf46*	1.34	2.77E-07	6.05E-05
TC0100018472.hg.1	*SEC22B*	1.34	7.15E-06	0.0006
TC1700011208.hg.1	*TRIM25; MIR3614*	1.33	8.98E-08	2.87E-05
TC1700007102.hg.1	*ALKBH5*	1.33	7.37E-07	0.0001
TC1200012083.hg.1	*FBXO21*	1.33	1.97E-06	0.0002
TC0700007811.hg.1	*INTS4P2*	1.33	0.0002	0.0065
TC0700013534.hg.1	*GGCT*	1.32	8.85E-07	0.0001
TC0300013838.hg.1	*ABHD14A*	1.31	8.30E-06	0.0006
TC0500009131.hg.1	*G3BP1*	1.31	3.20E-07	6.63E-05
TC0900009957.hg.1	*HINT2*	1.3	1.33E-05	0.0009
TC1500008231.hg.1	*AEN*	1.3	1.88E-05	0.0011
TC1600008259.hg.1	*PDPR*	1.3	7.96E-05	0.0032
TC1200007161.hg.1	*MRPS35*	1.3	2.00E-06	0.0002
TC1600011347.hg.1	*ALG1; NAGPA-AS1*	1.3	9.02E-06	0.0007
TC1900007688.hg.1	*CCNE1*	1.29	1.00E-06	0.0001
TC0400006579.hg.1	*ADD1*	1.29	1.13E-06	0.0002
TC1000007272.hg.1	*CREM*	1.29	0.0013	0.0219
TC1900009639.hg.1	*AP1M2*	1.29	3.01E-05	0.0016
TC0700011785.hg.1	*CDK6*	1.28	1.89E-07	4.66E-05
TC0700012780.hg.1	*HIPK2*	1.28	3.29E-07	6.66E-05
TC2200008036.hg.1	*USP41*	1.28	8.13E-05	0.0032
TC0300007410.hg.1	*GNAI2*	1.28	1.93E-07	4.70E-05
TC0200013096.hg.1	*MOB1A*	1.27	4.20E-07	7.77E-05
TC0800006566.hg.1	*AGPAT5*	1.27	3.60E-06	0.0004
TC1700006464.hg.1	*FAM57A*	1.27	4.75E-05	0.0022
TC1600009200.hg.1	*TRAP1*	1.27	2.99E-06	0.0003
TC0500013224.hg.1	*SLC27A6*	1.27	0.0014	0.0226
TC0900008927.hg.1	*NTMT1*	1.27	0.0029	0.0382
TC2000007942.hg.1	*FAM217B*	1.26	0.0018	0.0269
TC0900008219.hg.1	*NR4A3*	1.26	0.0145	0.1166

TC2000006504.hg.1	*STK35*	1.25	4.27E-06	0.0004
TC0800011137.hg.1	*RAD54B; FSBP*	1.25	2.51E-06	0.0003
TC0600006864.hg.1	*RIOK1*	1.25	0.0002	0.0066
TC1700007262.hg.1	*MAP2K3*	1.24	3.22E-06	0.0003
TC0600008073.hg.1	*SRF*	1.24	7.60E-06	0.0006
TC0100012593.hg.1	*WRAP73*	1.24	0.0026	0.0354
TC1200008942.hg.1	*PLBD2*	1.23	8.57E-07	0.0001
TC1100008447.hg.1	*SPCS2*	1.23	2.12E-05	0.0012
TC1600007235.hg.1	*PLK1*	1.23	3.11E-07	6.61E-05
TC1000008193.hg.1	*PPIF*	1.23	5.48E-07	9.33E-05
TC0400006706.hg.1	*TBC1D14*	1.23	1.39E-06	0.0002
TC1900006924.hg.1	*ZNF317*	1.23	1.45E-05	0.0010
TC0700011710.hg.1	*SLC25A40*	1.23	7.85E-07	0.0001
TC1500009109.hg.1	*C15orf57; MRPL42P5*	1.22	2.27E-06	0.0003
TC0100018473.hg.1	*SEC22B*	1.22	2.82E-06	0.0003
TC1600009942.hg.1	*CDIPT*	1.22	6.44E-06	0.0005
TC1000007876.hg.1	*DDX21*	1.22	2.13E-05	0.0012
TC2200008828.hg.1	*PHF5A*	1.21	3.59E-06	0.0004
TC0100006865.hg.1	*AGTRAP*	1.21	1.35E-05	0.0009
TC0900011778.hg.1	*RAPGEF1*	1.21	0.0002	0.0068
TC0200014672.hg.1	*NR4A2*	1.21	5.04E-05	0.0023
TC0300010329.hg.1	*NUP210*	1.2	7.42E-07	0.0001
TC0600012542.hg.1	*UBE2J1*	1.2	2.94E-06	0.0003
TC1700007254.hg.1	*DHRS7B*	1.2	1.83E-05	0.0011
TC0Y00006882.hg.1	*SLC25A6*	1.2	3.02E-05	0.0016
TC0500012599.hg.1	*ADAM19*	1.2	0.0007	0.0140
TC1100012422.hg.1	*APOA4*	1.2	0.0010	0.0180
TC0300013417.hg.1	*LIPH*	1.19	7.65E-06	0.0006
TC0X00010675.hg.1	*CUL4B*	1.19	5.41E-06	0.0005
TC0300007290.hg.1	*KLHL18*	1.19	2.95E-05	0.0016
TC0100016971.hg.1	*ADIPOR1*	1.19	8.27E-07	0.0001
TC0800012323.hg.1	*CA13*	1.18	1.62E-05	0.0010
TC2000008815.hg.1	*BCL2L1*	1.18	5.53E-06	0.0005
TC0600007311.hg.1	*HMGN4*	1.17	1.58E-06	0.0002
TC1700009277.hg.1	*TBCD*	1.17	8.32E-06	0.0006
TC0100010102.hg.1	*FLAD1*	1.17	1.04E-05	0.0007
TC0500006822.hg.1	*OTULIN*	1.17	0.0002	0.0058
TC2000008268.hg.1	*SLC23A2*	1.17	2.50E-05	0.0014
TC1000008431.hg.1	*PCGF5*	1.16	1.76E-06	0.0002
TC1600008226.hg.1	*NIP7*	1.16	2.91E-06	0.0003
TC0300013866.hg.1	*ALG1L2*	1.16	0.0002	0.0064
TC0300008331.hg.1	*ABHD10*	1.16	4.52E-06	0.0004
TC0100012846.hg.1	*DFFA*	1.16	2.91E-05	0.0016
TC0300009202.hg.1	*MBNL1*	1.15	1.48E-06	0.0002
TC1700009954.hg.1	*TOM1L2*	1.15	8.71E-05	0.0033
TC0600014140.hg.1	*PRIM2*	1.15	0.0068	0.0702

TC1700008886.hg.1	*SLC16A5*	1.14	2.99E-05	0.0016
TC1200009416.hg.1	*RAN*	1.14	3.01E-06	0.0003
TC1300008487.hg.1	*SLC7A1*	1.14	2.32E-05	0.0013
TC0900006881.hg.1	*IFT74*	1.14	0.0028	0.0374
TC0600011870.hg.1	*DNPH1*	1.14	1.32E-05	0.0009
TC1900011684.hg.1	*BABAM1*	1.14	0.0002	0.0063
TC1600007887.hg.1	*RBL2*	1.13	9.83E-06	0.0007
TC0200013114.hg.1	*M1AP*	1.13	1.41E-05	0.0009
TC0900009489.hg.1	*GLDC*	1.13	0.0042	0.0492
TC0100013226.hg.1	*USP48*	1.13	7.00E-06	0.0006
TC0700006618.hg.1	*WIPI2*	1.13	6.58E-05	0.0028
TC0600014348.hg.1	*NHSL1*	1.13	0.0001	0.0046
TC1100011128.hg.1	*ATL3*	1.13	3.49E-05	0.0018
TC1100009973.hg.1	*ARFIP2*	1.13	0.0003	0.0071
TC0100015921.hg.1	*ADAR*	1.12	6.42E-06	0.0005
TC1900009204.hg.1	*MOB3A*	1.12	1.25E-06	0.0002
TC0200014899.hg.1	*METTL8*	1.12	3.24E-05	0.0017
TC1500007518.hg.1	*RAB8B*	1.11	0.0002	0.0054
TC0300007566.hg.1	*SPATA12*	1.11	0.0053	0.0589
TC1700007918.hg.1	*AOC2*	1.11	0.0070	0.0711
TC1600010181.hg.1	*NETO2*	1.11	1.88E-06	0.0002
TC0700012798.hg.1	*KDM7A*	1.11	7.79E-06	0.0006
TC1100012681.hg.1	*SIAE*	1.11	8.10E-06	0.0006
TC0700011550.hg.1	*POM121C*	1.1	1.22E-05	0.0009
TC0600014148.hg.1	*CYB5R4*	1.1	3.41E-05	0.0017
TC1100009824.hg.1	*CARS*	1.1	0.0100	0.0907
TC1900006488.hg.1	*PTBP1; MIR4745*	1.1	1.93E-06	0.0002
TC1600011401.hg.1	*COQ9*	1.1	3.22E-05	0.0017
TC1600007022.hg.1	*ABCC1*	1.1	5.86E-05	0.0026
TC0400011414.hg.1	*TRMT10A*	1.1	0.0016	0.0243
TC0400007593.hg.1	*SRP72*	1.09	1.30E-05	0.0009
TC0700009337.hg.1	*C7orf55-LUC7L2; LUC7L2; C7orf55*	1.09	1.55E-05	0.0010
TC0900008891.hg.1	*LRRC8A*	1.09	6.89E-06	0.0006
TC0200013746.hg.1	*TGFBRAP1*	1.09	2.04E-05	0.0012
TC0200016767.hg.1	*MREG*	1.09	3.16E-05	0.0017
TC0500011984.hg.1	*HINT1*	1.09	0.0002	0.0061
TC0400009746.hg.1	*SLBP*	1.09	0.0005	0.0109
TC0200013257.hg.1	*TGOLN2*	1.08	0.0001	0.0040
TC0400011721.hg.1	*MAD2L1*	1.08	0.0005	0.0109
TC2200007904.hg.1	*ATP6V1E1*	1.08	0.0014	0.0229
TC0200007590.hg.1	*CHAC2*	1.08	0.0030	0.0388
TC1600011350.hg.1	*PMM2*	1.08	1.51E-05	0.0010
TC0100012454.hg.1	*SDF4*	1.08	1.67E-05	0.0010
TC1500010878.hg.1	*VWA9*	1.08	2.35E-06	0.0003
TC1500010660.hg.1	*PCSK6*	1.08	0.0004	0.0089
TC0500012059.hg.1	*VDAC1*	1.07	8.54E-07	0.0001

TC2100008297.hg.1	*SIK1*	1.07	9.50E-05	0.0036
TC0100009176.hg.1	*SNX7*	1.07	9.60E-05	0.0036
TC1200006561.hg.1	*RAD51AP1*	1.07	0.0001	0.0042
TC1200008544.hg.1	*NEDD1*	1.07	0.0008	0.0153
TC0200007363.hg.1	*MTA3*	1.07	4.25E-05	0.0021
TC0400008331.hg.1	*NPNT*	1.07	0.0304	0.1845
TC1600006628.hg.1	*KCTD5*	1.06	3.74E-06	0.0004
TC0700011575.hg.1	*POMZP3*	1.06	1.60E-05	0.0010
TC2000007016.hg.1	*GINS1*	1.06	9.94E-06	0.0007
TC0300008518.hg.1	*GTF2E1*	1.06	1.31E-05	0.0009
TC1200010971.hg.1	*DCTN2*	1.06	1.61E-05	0.0010
TC0200013104.hg.1	*MOGS*	1.06	2.42E-05	0.0014
TC1200009457.hg.1	*ULK1*	1.06	3.49E-05	0.0018
TC2000009823.hg.1	*STMN3*	1.06	0.0006	0.0133
TC1000012490.hg.1	*PI4K2A*	1.05	2.25E-05	0.0013
TC1200008920.hg.1	*OAS3*	1.05	2.58E-05	0.0014
TC0400008876.hg.1	*USP38*	1.05	0.0003	0.0071
TC1200007706.hg.1	*MFSD5*	1.05	0.0009	0.0163
TC1200012161.hg.1	*TRIAP1*	1.05	0.0017	0.0259
TC0100008978.hg.1	*LRRC8B*	1.05	0.0037	0.0450
TC2000009965.hg.1	*FKBP1A; MIR6869*	1.05	6.25E-06	0.0005
TC0X00008908.hg.1	*SLC25A6*	1.04	4.14E-05	0.0020
TC0900011035.hg.1	*TMEM246*	1.04	6.69E-05	0.0028
TC1500009236.hg.1	*MFAP1*	1.04	8.52E-06	0.0007
TC2100008098.hg.1	*DSCR3*	1.04	9.58E-06	0.0007
TC0100016625.hg.1	*GLUL*	1.04	0.0001	0.0040
TC1100011606.hg.1	*C2CD3*	1.04	0.0003	0.0071
TC0800012451.hg.1	*EIF3E*	1.03	3.98E-06	0.0004
TC1100012019.hg.1	*SESN3*	1.03	0.0011	0.0190
TC1100013012.hg.1	*STX3*	1.03	1.87E-06	0.0002
TC0500007668.hg.1	*CDK7*	1.03	1.60E-05	0.0010
TC2200007114.hg.1	*YWHAH*	1.03	2.28E-05	0.0013
TC2200007677.hg.1	*TBC1D22A*	1.03	0.0005	0.0111
TC1300006503.hg.1	*IFT88*	1.03	0.0006	0.0133
TC1600008301.hg.1	*CALB2*	1.03	0.0059	0.0633
TC1600011545.hg.1	*PLLP*	1.03	0.0137	0.1129
TC0500009837.hg.1	*SLC12A7*	1.02	6.18E-05	0.0027
TC0X00008108.hg.1	*TMEM164*	1.02	0.0084	0.0802
TC0300010357.hg.1	*CHCHD4*	1.02	4.94E-05	0.0023
TC0X00009007.hg.1	*PUDP*	1.01	0.0004	0.0100
TC1900007270.hg.1	*KLF2*	1.01	0.0004	0.0101
TC0X00008844.hg.1	*IKBKG*	1.01	0.0030	0.0387
TC1100010893.hg.1	*SLC43A1*	1.01	0.0072	0.0723
TC0100014918.hg.1	*SETSIP*	1.01	0.0073	0.0732
TC2200008611.hg.1	*EIF3D*	1.01	9.82E-06	0.0007
TC1600011329.hg.1	*TBC1D24*	1.01	1.52E-05	0.0010

TC1400006821.hg.1	*G2E3*	1.01	0.0107	0.0955
TC1400009184.hg.1	*TXNDC16*	1.01	0.0115	0.1001
TC1100013054.hg.1	*RAD9A*	1	0.0002	0.0061
TC0900012176.hg.1	*URM1*	1	4.10E-05	0.0020
TC0100015049.hg.1	*FRRS1*	1	0.0004	0.0104
TC0X00010837.hg.1	*MBNL3*	-0.99	5.73E-05	0.0026
TC1500010018.hg.1	*SEMA7A*	-0.99	8.15E-05	0.0032
TC0100006681.hg.1	*RNF207*	-1	0.0126	0.1064
TC1200008843.hg.1	*FAM216A*	-1.01	0.0002	0.0061
TC0600007821.hg.1	*LHFPL5*	-1.01	0.0014	0.0224
TC0600011252.hg.1	*ZSCAN31*	-1.01	0.0328	0.1934
TC1700011313.hg.1	*USP32*	-1.01	1.19E-05	0.0008
TC0200015002.hg.1	*CHN1*	-1.01	5.02E-05	0.0023
TC1200007887.hg.1	*MBD6*	-1.01	7.63E-05	0.0031
TC0300013104.hg.1	*EIF5A2*	-1.01	0.0001	0.0037
TC0800007335.hg.1	*LETM2*	-1.01	0.0001	0.0037
TC0400007495.hg.1	*DCUN1D4*	-1.01	0.0002	0.0053
TC1100006831.hg.1	*ADM*	-1.01	0.0004	0.0090
TC1200010229.hg.1	*PTHLH*	-1.01	0.0049	0.0558
TC0500013089.hg.1	*GFPT2*	-1.01	0.0357	0.2041
TC0700011518.hg.1	*GTF2IRD2; GTF2IRD2B*	-1.02	3.92E-06	0.0004
TC0800009927.hg.1	*EBF2*	-1.02	0.0001	0.0037
TC1700011919.hg.1	*CEP295NL; TIMP2*	-1.02	3.62E-06	0.0004
TC0800010918.hg.1	*ZNF704*	-1.02	4.64E-05	0.0022
TC0500008835.hg.1	*CXXC5*	-1.02	0.0001	0.0048
TC0400012952.hg.1	*BDH2*	-1.02	0.0002	0.0069
TC1000006466.hg.1	*WDR37*	-1.02	0.0006	0.0133
TC1900006685.hg.1	*FSD1*	-1.02	0.0045	0.0519
TC1600006537.hg.1	*BAIAP3*	-1.02	0.0062	0.0656
TC0200010416.hg.1	*SPATS2L*	-1.02	0.0286	0.1783
TC1300007774.hg.1	*MBNL2*	-1.02	5.06E-06	0.0005
TC0200016717.hg.1	*TBC1D8*	-1.02	0.0001	0.0043
TC2200007312.hg.1	*LGALS1*	-1.02	0.0004	0.0100
TC1900012025.hg.1	*ZNF28*	-1.02	0.0163	0.1257
TC0500012909.hg.1	*DRD1*	-1.03	0.0002	0.0068
TC1200010284.hg.1	*DENND5B*	-1.03	0.0006	0.0129
TC0400011144.hg.1	*ANTXR2*	-1.03	0.0011	0.0191
TC1000011938.hg.1	*SHTN1*	-1.03	3.37E-05	0.0017
TC2200008799.hg.1	*ST13*	-1.04	2.49E-06	0.0003
SUnmapped00000818.hg	*F10*	-1.04	3.02E-05	0.0016
TC1600011517.hg.1	*DOC2A*	-1.04	6.92E-05	0.0029
TC0300012665.hg.1	*XRN1*	-1.04	0.0035	0.0436
TC1900010716.hg.1	*ADCK4*	-1.04	0.0104	0.0931
TC1900007399.hg.1	*TMEM59L*	-1.04	1.29E-05	0.0009
TC1800008399.hg.1	*B4GALT6*	-1.04	6.59E-05	0.0028
TC0600010002.hg.1	*SYNJ2*	-1.04	0.0008	0.0151

TC1600007811.hg.1	*PAPD5*	-1.04	0.0011	0.0190
TC0300007296.hg.1	*SCAP*	-1.04	0.0226	0.1538
TC0700012813.hg.1	*DENND2A*	-1.04	0.0331	0.1944
TC0300011135.hg.1	*VPRBP*	-1.05	0.0001	0.0043
TC0X00010136.hg.1	*TAF9B*	-1.05	0.0003	0.0078
TC1600006593.hg.1	*RAB26*	-1.05	1.65E-06	0.0002
TC0300012029.hg.1	*NAA50*	-1.05	1.71E-06	0.0002
TC0500011751.hg.1	*FEM1C*	-1.05	6.50E-06	0.0005
TC0100013339.hg.1	*RUNX3*	-1.05	1.33E-05	0.0009
TC1000008954.hg.1	*CASP7*	-1.05	4.49E-05	0.0022
TC0200011171.hg.1	*SH3BP4*	-1.05	0.0003	0.0072
TC0400007868.hg.1	*PARM1*	-1.05	0.0028	0.0372
TC0100012222.hg.1	*DESI2*	-1.05	3.93E-06	0.0004
TC0800010285.hg.1	*SLC20A2*	-1.06	0.0001	0.0043
TC1400008382.hg.1	*TNFAIP2*	-1.06	0.0005	0.0107
TC0900007518.hg.1	*MAMDC2*	-1.06	0.0005	0.0110
TC0700008367.hg.1	*PEG10*	-1.06	0.0212	0.1488
TC0500007337.hg.1	*PARP8*	-1.06	4.20E-06	0.0004
TC1700012457.hg.1	*AXIN2*	-1.06	9.18E-06	0.0007
TC1100009330.hg.1	*UBASH3B*	-1.06	6.12E-05	0.0027
TC1100012583.hg.1	*BLID; MIR100HG*	-1.06	8.31E-05	0.0032
TC0900010386.hg.1	*TMEM2*	-1.06	9.33E-05	0.0035
TC1100009026.hg.1	*SIK2*	-1.06	0.0002	0.0066
TC1000008658.hg.1	*PAX2*	-1.06	0.0006	0.0133
TC1900011770.hg.1	*C5AR1*	-1.06	0.0010	0.0178
TC0300012339.hg.1	*GATA2*	-1.06	0.0024	0.0334
TC1500010162.hg.1	*RASGRF1*	-1.06	0.0038	0.0459
TC1700006744.hg.1	*NLGN2*	-1.06	0.0184	0.1367
TC0100017761.hg.1	*PCNXL2*	-1.06	0.0199	0.1432
TC1000008354.hg.1	*PAPSS2*	-1.07	0.0007	0.0143
TC0100018236.hg.1	*ACADM*	-1.07	1.12E-05	0.0008
TC1900007290.hg.1	*TMEM38A*	-1.07	9.37E-05	0.0035
TC0500013140.hg.1	*AHRR; PDCD6*	-1.07	0.0015	0.0237
TC1700010996.hg.1	*HOXB8*	-1.07	2.69E-05	0.0015
TC0800011881.hg.1	*NDRG1*	-1.07	0.0010	0.0182
TC0100010393.hg.1	*RGS4*	-1.08	0.0002	0.0056
TC0900011501.hg.1	*NR6A1*	-1.08	0.0002	0.0069
TC0900011981.hg.1	*NOTCH1*	-1.08	0.0111	0.0977
TC0X00010877.hg.1	*FAM122B*	-1.08	5.91E-06	0.0005
TC1100013059.hg.1	*MYEOV*	-1.08	1.35E-05	0.0009
TC1900008015.hg.1	*SPRED3*	-1.08	0.0008	0.0157
TC0800011634.hg.1	*SNTB1*	-1.09	1.02E-06	0.0001
TC0300014021.hg.1	*CPOX*	-1.09	1.13E-05	0.0008
TC1500009457.hg.1	*MYO5A*	-1.09	0.0013	0.0211
TC0300012935.hg.1	*SHOX2*	-1.09	9.25E-06	0.0007
SUnmapped00000398.hg	*KAT6B*	-1.09	1.61E-05	0.0010

TC2000006721.hg.1	*BTBD3*	-1.09	7.07E-05	0.0030
TC0900010920.hg.1	*AAED1*	-1.09	9.29E-05	0.0035
TC0800007221.hg.1	*NRG1*	-1.09	0.0002	0.0049
TC1900011233.hg.1	*KLK10*	-1.09	0.0004	0.0101
TC1100010897.hg.1	*UBE2L6*	-1.09	0.0011	0.0194
TC1100013223.hg.1	*CARD17*	-1.09	0.0016	0.0244
TC0400007853.hg.1	*EREG*	-1.1	1.51E-06	0.0002
TC2100008385.hg.1	*SUMO3*	-1.1	6.11E-05	0.0027
TC0300006791.hg.1	*KAT2B*	-1.1	8.45E-05	0.0033
TC1600008034.hg.1	*NDRG4*	-1.1	0.0003	0.0087
TC1500009508.hg.1	*NEDD4*	-1.1	0.0008	0.0151
TC0800009735.hg.1	*MTUS1*	-1.1	2.49E-05	0.0014
TC0700013468.hg.1	*NDUFB2*	-1.1	4.19E-05	0.0020
TC0200012713.hg.1	*BCL11A*	-1.1	0.0004	0.0095
TC0500008539.hg.1	*GRAMD3*	-1.1	0.0307	0.1858
TC0700011298.hg.1	*ZNF680*	-1.11	1.04E-05	0.0007
TC1700010540.hg.1	*PCGF2*	-1.11	7.15E-06	0.0006
TC0500010615.hg.1	*SEPP1*	-1.11	0.0011	0.0190
TC1100012230.hg.1	*EXPH5*	-1.11	3.74E-06	0.0004
TC0100011096.hg.1	*NEK7*	-1.11	1.03E-05	0.0007
TC0200009443.hg.1	*R3HDM1*	-1.11	5.46E-05	0.0025
TC0100010112.hg.1	*SLC50A1*	-1.12	4.66E-06	0.0004
TC0100007789.hg.1	*ago-03*	-1.12	5.45E-05	0.0025
TC0500008479.hg.1	*SNX2*	-1.12	0.0002	0.0061
TC1700008862.hg.1	*RAB37*	-1.12	3.10E-05	0.0016
TC0500009061.hg.1	*PCYOX1L*	-1.12	0.0001	0.0047
TC1000010482.hg.1	*C10orf10*	-1.12	4.89E-05	0.0023
TC0900008793.hg.1	*ZBTB34*	-1.12	0.0015	0.0237
TC0100018454.hg.1	*ARHGAP29*	-1.13	3.21E-06	0.0003
TC1700012185.hg.1	*TNFSF12*	-1.13	0.0007	0.0135
TC1300008439.hg.1	*MTIF3*	-1.13	0.0080	0.0777
TC0500009488.hg.1	*CREBRF*	-1.14	3.54E-06	0.0004
TC1900010433.hg.1	*SCGB2B2*	-1.14	0.0310	0.1868
TC1600011317.hg.1	*NME4*	-1.14	9.46E-06	0.0007
TC0800006496.hg.1	*MYOM2*	-1.14	3.36E-05	0.0017
TC1900009431.hg.1	*SLC25A23*	-1.14	5.24E-05	0.0024
TC1900011029.hg.1	*MEIS3*	-1.14	6.04E-05	0.0027
TC0400011180.hg.1	*SCD5*	-1.14	0.0002	0.0055
TC1200008535.hg.1	*ELK3*	-1.14	0.0002	0.0061
TC1700012191.hg.1	*CD68*	-1.14	0.0003	0.0072
TC2100008562.hg.1	*RUNX1*	-1.14	5.11E-06	0.0005
TC1900010807.hg.1	*CEACAM1*	-1.15	1.86E-05	0.0011
TC0100015572.hg.1	*SRGAP2B*	-1.15	1.91E-05	0.0011
TC0500012210.hg.1	*TMEM173*	-1.15	7.22E-07	0.0001
TC1400008705.hg.1	*SLC7A8*	-1.15	2.61E-05	0.0014
TC0400008879.hg.1	*GAB1*	-1.15	8.25E-05	0.0032

TC0100016018.hg.1	*CRABP2*	-1.16	2.11E-06	0.0002
TC1000011659.hg.1	*MGEA5*	-1.16	7.47E-07	0.0001
TC0100010121.hg.1	*HCN3*	-1.16	1.65E-05	0.0010
TC1300008497.hg.1	*UBL3*	-1.16	0.0005	0.0111
TC0X00010207.hg.1	*POF1B*	-1.16	0.0083	0.0798
TC0400009033.hg.1	*TRIM2*	-1.16	3.05E-06	0.0003
TC0400007938.hg.1	*BMP2K*	-1.17	3.46E-06	0.0004
TC1100013144.hg.1	*ZBED5*	-1.17	1.67E-05	0.0010
TC1000011081.hg.1	*ZNF503*	-1.17	6.77E-05	0.0029
TC0900008150.hg.1	*TMOD1*	-1.17	0.0002	0.0056
TC0100012921.hg.1	*DHRS3; MIR6730*	-1.17	0.0040	0.0479
TC0100010755.hg.1	*RALGPS2*	-1.17	3.16E-05	0.0017
TC1200008597.hg.1	*GAS2L3*	-1.17	6.55E-05	0.0028
TC1200011786.hg.1	*NUAK1*	-1.17	0.0007	0.0142
TC0300007572.hg.1	*APPL1*	-1.18	0.0003	0.0078
TC1700012279.hg.1	*HOXB-AS3*	-1.18	0.0042	0.0499
TC0100016678.hg.1	*FAM129A*	-1.18	0.0051	0.0574
TC0100017860.hg.1	*HEATR1*	-1.18	3.78E-06	0.0004
TC1100011602.hg.1	*UCP2*	-1.18	0.0003	0.0085
TC1200010252.hg.1	*TMTC1*	-1.19	5.91E-05	0.0026
TC1200010569.hg.1	*ASB8*	-1.19	0.0237	0.1585
TC1000008585.hg.1	*MARVELD1*	-1.2	1.64E-05	0.0010
TC1000007641.hg.1	*DKK1*	-1.2	2.61E-05	0.0014
TC0700013531.hg.1	*HOXA6*	-1.2	9.77E-05	0.0036
TC1900010743.hg.1	*TGFB1*	-1.2	0.0006	0.0121
TC0200008803.hg.1	*GCC2*	-1.2	0.0008	0.0149
TC1400009194.hg.1	*FERMT2*	-1.2	0.0008	0.0158
TC1900011231.hg.1	*KLK7*	-1.2	1.83E-06	0.0002
TC1000006802.hg.1	*CAMK1D*	-1.2	8.34E-06	0.0006
TC0400011643.hg.1	*CAMK2D*	-1.2	9.43E-06	0.0007
TC2200009281.hg.1	*PNPLA3*	-1.2	0.0088	0.0833
TC1100009431.hg.1	*PATE4*	-1.21	0.0053	0.0585
TC1900011153.hg.1	*RRAS*	-1.22	0.0004	0.0100
TC0400008450.hg.1	*ANK2*	-1.23	3.15E-05	0.0017
TC1700012276.hg.1	*PNPO*	-1.23	0.0002	0.0069
TC2000009058.hg.1	*TGM2*	-1.23	8.99E-05	0.0034
TC1700010604.hg.1	*NR1D1*	-1.24	6.07E-06	0.0005
TC0100008594.hg.1	*ROR1*	-1.24	7.52E-05	0.0031
TC0200007237.hg.1	*CRIM1*	-1.24	5.25E-06	0.0005
TC1900011194.hg.1	*KCNC3*	-1.24	0.0001	0.0038
TC0X00011279.hg.1	*TSPAN7*	-1.24	0.0019	0.0282
TC1000009792.hg.1	*USP6NL*	-1.24	3.22E-07	6.63E-05
TC0700013371.hg.1	*ZNF138*	-1.25	8.18E-05	0.0032
TC0800010524.hg.1	*IMPAD1*	-1.25	3.18E-07	6.63E-05
TC0100014752.hg.1	*GNG5*	-1.25	7.23E-06	0.0006
TC0800010282.hg.1	*DKK4*	-1.25	0.0001	0.0041

TC1200006738.hg.1	*KLRG1*	-1.26	0.0007	0.0135
TC0300010632.hg.1	*OSBPL10*	-1.26	2.16E-07	5.05E-05
TC1300008983.hg.1	*INTS6*	-1.26	2.93E-07	6.35E-05
TC1000009067.hg.1	*GRK5*	-1.27	9.15E-07	0.0001
TC0100011364.hg.1	*SRGAP2*	-1.27	4.82E-06	0.0004
TC2200006827.hg.1	*GNAZ*	-1.27	5.05E-05	0.0023
TC1500007619.hg.1	*SMAD6*	-1.27	1.81E-05	0.0011
TC1700010653.hg.1	*KRTAP2-3*	-1.27	8.52E-05	0.0033
TC1900011996.hg.1	*CGB8*	-1.28	3.63E-06	0.0004
TC0700010563.hg.1	*HOXA7*	-1.28	8.70E-06	0.0007
TC1100007266.hg.1	*PDHX*	-1.28	3.40E-05	0.0017
TC1700010657.hg.1	*KRTAP4-12*	-1.29	2.92E-05	0.0016
TC1400007168.hg.1	*GPR137C*	-1.29	0.0004	0.0090
TC2200009153.hg.1	*DENND6B*	-1.29	0.0005	0.0119
TC1100013131.hg.1	*IGF2*	-1.29	0.0006	0.0133
TC1700007944.hg.1	*TMEM106A*	-1.3	7.64E-07	0.0001
TC1700012434.hg.1	*HOXB7*	-1.3	6.02E-06	0.0005
TC1000007199.hg.1	*ZEB1*	-1.31	5.48E-05	0.0025
TC1100007507.hg.1	*PTPRJ*	-1.31	4.04E-07	7.60E-05
TC0400011263.hg.1	*SPARCL1*	-1.31	1.97E-05	0.0012
TC0X00008514.hg.1	*DDX26B*	-1.31	0.0002	0.0061
TC1200008269.hg.1	*NAV3*	-1.32	6.42E-05	0.0028
TC1000011661.hg.1	*KCNIP2*	-1.32	4.15E-07	7.74E-05
TC0900007552.hg.1	*GDA*	-1.33	1.17E-07	3.38E-05
TC1900006865.hg.1	*TGFBR3L*	-1.33	7.67E-06	0.0006
TC1900011084.hg.1	*DBP*	-1.33	9.47E-07	0.0001
TC0600011945.hg.1	*RCAN2*	-1.34	9.04E-07	0.0001
TC1100008969.hg.1	*ELMOD1*	-1.34	5.72E-05	0.0026
TC0600008697.hg.1	*NT5E*	-1.35	4.26E-05	0.0021
TC0600009350.hg.1	*RNF217*	-1.35	7.46E-05	0.0031
TC2200007489.hg.1	*C22orf46*	-1.35	1.99E-06	0.0002
TC2000008242.hg.1	*RNF24*	-1.35	2.13E-06	0.0002
TC0600014191.hg.1	*SAMD5*	-1.36	5.44E-07	9.33E-05
TC1100011141.hg.1	*RCOR2*	-1.36	4.83E-05	0.0023
TC0X00009215.hg.1	*EIF1AX*	-1.37	2.45E-06	0.0003
TC1200010806.hg.1	*MAP3K12*	-1.37	9.77E-06	0.0007
TC0100016833.hg.1	*ZBTB41*	-1.38	1.53E-06	0.0002
TC0X00009057.hg.1	*MID1*	-1.38	2.84E-06	0.0003
TC1000007954.hg.1	*SLC29A3*	-1.38	1.19E-05	0.0008
TC1100011888.hg.1	*CTSC*	-1.39	3.18E-05	0.0017
TC1000007761.hg.1	*ARID5B*	-1.4	1.77E-06	0.0002
TC0300012980.hg.1	*SPTSSB*	-1.4	4.42E-06	0.0004
TC1000010273.hg.1	*NRP1*	-1.4	9.82E-06	0.0007
TC1400009353.hg.1	*SIX4*	-1.4	6.53E-05	0.0028
TC0300012764.hg.1	*HLTF*	-1.4	3.80E-06	0.0004
TC1900009603.hg.1	*OLFM2*	-1.4	0.0004	0.0089

TC1400007320.hg.1	*DACT1*	-1.41	7.16E-07	0.0001
TC1100011833.hg.1	*SYTL2*	-1.41	2.90E-06	0.0003
TC0100013796.hg.1	*PABPC4*	-1.41	1.50E-06	0.0002
TC2000009955.hg.1	*SLC2A4RG*	-1.41	0.0004	0.0090
TC0400009038.hg.1	*KIAA0922*	-1.41	2.23E-06	0.0003
TC1200009734.hg.1	*VAMP1*	-1.42	1.14E-06	0.0002
TC1400009006.hg.1	*TRAPPC6B*	-1.43	3.50E-07	6.84E-05
TC1400009134.hg.1	*SAV1*	-1.43	2.57E-06	0.0003
SUnmapped00000049.hg	*PADI3*	-1.43	6.44E-05	0.0028
TC1200012711.hg.1	*LINC00173*	-1.44	0.0001	0.0047
TC1900011067.hg.1	*TMEM143*	-1.44	3.52E-07	6.84E-05
TC1100013130.hg.1	*IGF2; INS-IGF2*	-1.44	0.0003	0.0075
TC0400010168.hg.1	*LCORL*	-1.45	3.35E-08	1.47E-05
TC0300011259.hg.1	*ARHGEF3*	-1.46	3.68E-06	0.0004
TC0X00010840.hg.1	*HS6ST2*	-1.46	6.38E-06	0.0005
TC1700011558.hg.1	*SLC16A6*	-1.46	0.0001	0.0038
TC1900010005.hg.1	*RAB3A*	-1.46	2.01E-05	0.0012
TC1200007931.hg.1	*SLC16A7*	-1.46	8.15E-07	0.0001
TC1400009481.hg.1	*PLEK2*	-1.47	5.41E-08	2.07E-05
TC1700006524.hg.1	*SERPINF1*	-1.47	2.24E-07	5.17E-05
TC0300013949.hg.1	*SATB1*	-1.47	2.10E-07	4.95E-05
TC0200014694.hg.1	*ACVR1C*	-1.47	2.15E-05	0.0013
TC1700010651.hg.1	*KRTAP2-1*	-1.47	0.0003	0.0072
TC0300013108.hg.1	*TNIK*	-1.48	4.98E-08	2.05E-05
TC0600008146.hg.1	*RUNX2*	-1.48	2.38E-07	5.43E-05
TC1800007298.hg.1	*LIPG*	-1.48	2.09E-06	0.0002
TC0600014192.hg.1	*RP11-307P5.1; SAMD5*	-1.49	0.0003	0.0078
TC0600011943.hg.1	*ENPP5*	-1.5	5.26E-08	2.07E-05
TC0500010876.hg.1	*SMIM15*	-1.51	1.32E-07	3.69E-05
TC1500006925.hg.1	*THBS1*	-1.51	2.36E-05	0.0013
TC0200009955.hg.1	*CYBRD1*	-1.52	6.75E-07	0.0001
TC1900008286.hg.1	*BCAM*	-1.52	7.55E-07	0.0001
TC2200006883.hg.1	*SUSD2*	-1.52	0.0001	0.0038
TC1300008609.hg.1	*DCLK1*	-1.52	1.77E-06	0.0002
TC1200008028.hg.1	*MSRB3*	-1.52	1.62E-05	0.0010
TC0500010540.hg.1	*LIFR*	-1.54	3.57E-06	0.0004
TC0500012519.hg.1	*SPARC*	-1.54	5.00E-06	0.0004
TC0700011318.hg.1	*ERV3-1; ZNF117*	-1.54	7.36E-06	0.0006
TC1700012282.hg.1	*SPATA20*	-1.55	1.21E-07	3.46E-05
TC1700010652.hg.1	*KRTAP2-2*	-1.57	8.72E-05	0.0033
TC0600009019.hg.1	*SOBP*	-1.57	5.11E-06	0.0005
TC0100011022.hg.1	*RGS2*	-1.59	4.26E-07	7.81E-05
TC0300012781.hg.1	*WWTR1*	-1.59	5.81E-06	0.0005
TC0X00007176.hg.1	*SSX1*	-1.59	0.0011	0.0187
TC1600010978.hg.1	*PKD1L2*	-1.6	1.71E-05	0.0011
TC1600011328.hg.1	*NPW*	-1.6	6.37E-06	0.0005

TC0600014111.hg.1	*SYNGAP1; MIR5004*	-1.62	3.29E-07	6.66E-05
TC1700012110.hg.1	*SECTM1*	-1.64	2.59E-07	5.73E-05
TC0500013149.hg.1	*SRD5A1*	-1.64	0.0006	0.0133
TC0600007650.hg.1	*HLA-DRA*	-1.65	1.12E-07	3.33E-05
TC0100015596.hg.1	*LIX1L*	-1.65	0.0001	0.0037
TC0800009891.hg.1	*STC1*	-1.66	1.22E-07	3.46E-05
TC0500011648.hg.1	*EFNA5*	-1.66	2.00E-08	1.10E-05
TC0400011600.hg.1	*PITX2*	-1.68	0.0020	0.0288
TC1000006796.hg.1	*SEC61A2*	-1.69	7.04E-08	2.41E-05
TC1600009162.hg.1	*HCFC1R1*	-1.7	1.86E-07	4.65E-05
TC1200012651.hg.1	*KIF5A*	-1.71	4.79E-07	8.57E-05
TC0X00010515.hg.1	*CAPN6*	-1.71	1.52E-05	0.0010
TC0200016648.hg.1	*CDC42EP3*	-1.71	1.31E-05	0.0009
TC0100011148.hg.1	*CAMSAP2*	-1.72	9.11E-08	2.87E-05
TC0400011994.hg.1	*INPP4B*	-1.72	2.23E-06	0.0003
TC0300013123.hg.1	*PLD1*	-1.74	9.81E-08	3.00E-05
TC0600007087.hg.1	*CAP2*	-1.74	1.13E-07	3.33E-05
TC1700012354.hg.1	*VAMP2*	-1.75	2.13E-06	0.0002
TC1700010625.hg.1	*TNS4*	-1.75	1.30E-08	8.46E-06
TC1900009186.hg.1	*ATP8B3*	-1.76	7.08E-08	2.41E-05
TC0700007034.hg.1	*CREB5*	-1.76	7.06E-05	0.0030
TC1000009414.hg.1	*INPP5A*	-1.77	2.50E-08	1.29E-05
TC1100009068.hg.1	*NCAM1*	-1.77	6.94E-08	2.41E-05
TC0600011957.hg.1	*ADGRF1*	-1.78	7.56E-06	0.0006
TC1700008263.hg.1	*ABCC3*	-1.79	2.47E-08	1.29E-05
TC0600009327.hg.1	*PKIB*	-1.79	3.07E-08	1.43E-05
TC1700010997.hg.1	*HOXB9*	-1.81	3.26E-08	1.47E-05
TC1400008714.hg.1	*SLC22A17*	-1.81	3.57E-05	0.0018
TC0600011953.hg.1	*PLA2G7*	-1.83	6.16E-05	0.0027
TC0300012048.hg.1	*ZBTB20; MIR568*	-1.83	4.71E-07	8.49E-05
TC1000009927.hg.1	*ST8SIA6*	-1.84	9.44E-07	0.0001
TC0200015364.hg.1	*SATB2*	-1.89	1.58E-08	9.15E-06
TC0100011378.hg.1	*RASSF5*	-1.9	6.41E-06	0.0005
TC0800010382.hg.1	*SNAI2*	-1.9	3.52E-05	0.0018
TC1800009268.hg.1	*DSC2*	-1.91	2.79E-09	3.32E-06
TC1800007360.hg.1	*RAB27B*	-1.91	1.53E-07	3.99E-05
TC0600009439.hg.1	*TMEM200A*	-1.91	1.24E-08	8.32E-06
TC0100009373.hg.1	*SLC6A17*	-1.92	2.79E-05	0.0015
TC0300009855.hg.1	*IL1RAP*	-1.93	5.86E-08	2.17E-05
TC0400008943.hg.1	*EDNRA*	-1.93	2.53E-05	0.0014
TC0500011418.hg.1	*MEF2C*	-1.97	4.25E-08	1.82E-05
TC0800006864.hg.1	*ZDHHC2*	-1.98	9.06E-10	1.56E-06
TC0500007834.hg.1	*F2RL1*	-1.99	3.51E-10	8.36E-07
TC0900010886.hg.1	*PTCH1*	-2.07	4.59E-10	9.84E-07
TC1700012433.hg.1	*HOXB6*	-2.08	1.64E-10	5.02E-07
TC1200010182.hg.1	*BHLHE41*	-2.1	1.06E-08	7.86E-06

TC0200008058.hg.1	*C2orf78*	-2.23	1.18E-08	8.16E-06
TC0500007972.hg.1	*VCAN*	-2.26	1.31E-09	1.88E-06
TC0100016476.hg.1	*KIAA0040*	-2.71	9.04E-11	4.85E-07
TC0100016218.hg.1	*RGS5*	-2.77	2.61E-10	7.00E-07
TC0100009142.hg.1	*PTBP2*	-3.69	2.21E-13	4.73E-09

The fold change was calculated as (expression in control cells)/(expression in cells silenced for *ZNF518B*) and expressed as logarithm in base 2

Supplementary Table S4. Genes significantly altered by *EHMT2* or *EZH2* silencing in HCT116 cells as retrieved from the GEO database

a) si*EZH2*

ENSEMBL ID	Gene Symbol	Bowtie2			STAR		
		logFC	P-Value	FDR	logFC	P-Value	FDR
ENSG00000197915	*HRNR*	3.804	5.27E-30	3.42E-25	4.303	2.52E-49	1.64E-44
ENSG00000099250	*NRP1*	-2.484	3.33E-26	1.08E-21	-2.460	3.13E-26	1.02E-21
ENSG00000143631	*FLG*	5.345	8.53E-18	1.11E-13	5.949	3.93E-25	8.48E-21
ENSG00000171903	*CYP4F11*	2.234	5.29E-21	1.14E-16	2.223	2.50E-21	4.05E-17
ENSG00000110492	*MDK*	-1.947	1.33E-19	2.16E-15	-1.961	6.22E-20	8.07E-16
ENSG00000245532	*NEAT1*	1.795	1.81E-17	1.96E-13	1.790	2.20E-17	2.38E-13
ENSG00000135074	*ADAM19*	-2.305	6.38E-16	5.91E-12	-2.288	1.73E-16	1.60E-12
ENSG00000137491	*SLCO2B1*	3.691	8.46E-16	6.85E-12	3.616	3.34E-16	2.70E-12
ENSG00000186529	*CYP4F3*	2.233	3.93E-15	2.55E-11	2.231	4.98E-16	3.59E-12
ENSG00000143183	*TMCO1*	-1.684	1.01E-14	5.95E-11	-1.740	6.45E-16	4.18E-12
ENSG00000176485	*PLAAT3*	-2.236	3.51E-15	2.53E-11	-2.097	1.41E-14	8.33E-11
ENSG00000198074	*AKR1B10*	2.439	8.24E-13	3.82E-09	2.409	2.56E-14	1.28E-10
ENSG00000105329	*TGFB1*	-1.594	1.67E-12	7.21E-09	-1.628	3.91E-13	1.49E-09
ENSG00000157570	*TSPAN18*	-1.791	3.50E-12	1.41E-08	-1.845	4.63E-13	1.65E-09
ENSG00000035862	*TIMP2*	-1.604	3.46E-13	1.87E-09	-1.592	5.04E-13	1.65E-09
ENSG00000163435	*ELF3*	1.794	3.70E-12	1.41E-08	1.829	5.08E-13	1.65E-09
ENSG00000146648	*EGFR*	-1.555	4.21E-13	2.10E-09	-1.543	5.57E-13	1.72E-09
ENSG00000144063	*MALL*	-1.395	9.19E-11	2.35E-07	-1.493	1.87E-12	5.51E-09
ENSG00000156510	*HKDC1*	1.707	6.94E-12	2.20E-08	1.671	4.37E-12	1.20E-08
ENSG00000167549	*CORO6*	1.598	6.18E-12	2.20E-08	1.599	4.45E-12	1.20E-08
ENSG00000145284	*SCD5*	-1.626	6.97E-12	2.20E-08	-1.612	6.88E-12	1.79E-08
ENSG00000182158	*CREB3L2*	-1.495	2.44E-11	7.18E-08	-1.517	1.07E-11	2.67E-08
ENSG00000006016	*CRLF1*	-1.588	9.24E-11	2.35E-07	-1.612	2.61E-11	6.04E-08
ENSG00000078018	*MAP2*	1.516	7.11E-12	2.20E-08	1.458	3.19E-11	7.14E-08
ENSG00000118596	*SLC16A7*	-1.410	1.50E-10	3.60E-07	-1.443	5.30E-11	1.14E-07
ENSG00000166147	*FBN1*	2.139	2.52E-10	5.28E-07	2.110	5.71E-11	1.19E-07
ENSG00000082146	*STRADB*	-1.419	3.79E-08	3.62E-05	-1.607	6.43E-11	1.30E-07
ENSG00000186480	*INSIG1*	1.374	9.43E-11	2.35E-07	1.360	1.31E-10	2.58E-07
ENSG00000140961	*OSGIN1*	1.452	5.01E-11	1.41E-07	1.405	1.54E-10	2.94E-07
ENSG00000137414	*FAM8A1*	-1.412	1.85E-10	4.13E-07	-1.411	1.66E-10	3.07E-07
ENSG00000142192	*APP*	-1.303	4.68E-10	7.98E-07	-1.330	1.96E-10	3.53E-07
ENSG00000155629	*PIK3AP1*	-1.723	2.02E-10	4.36E-07	-1.646	3.03E-10	5.16E-07
ENSG00000178852	*EFCAB13*	1.478	2.68E-10	5.43E-07	1.468	3.31E-10	5.37E-07
ENSG00000164484	*TMEM200A*	-1.600	3.68E-10	6.44E-07	-1.586	4.08E-10	6.45E-07
ENSG00000076770	*MBNL3*	-1.361	3.60E-10	6.44E-07	-1.354	4.18E-10	6.45E-07
ENSG00000171931	*FBXW10*	1.640	3.77E-05	0.00938	1.976	5.38E-10	8.11E-07
ENSG00000117322	*CR2*	-1.778	3.68E-09	5.43E-06	-1.786	5.53E-10	8.15E-07
ENSG00000174498	*IGDCC3*	-2.703	4.81E-10	8.00E-07	-2.507	9.20E-10	1.33E-06
ENSG00000117148	*ACTL8*	-1.496	3.42E-10	6.34E-07	-1.439	9.78E-10	1.35E-06
ENSG00000169499	*PLEKHA2*	-1.468	6.46E-09	8.21E-06	-1.509	1.50E-09	2.02E-06
ENSG00000272398	*CD24*	-1.644	1.67E-07	0.00013	-1.737	2.46E-09	3.24E-06
ENSG00000164171	*ITGA2*	-1.251	3.30E-09	4.97E-06	-1.257	2.50E-09	3.24E-06
ENSG00000163297	*ANTXR2*	-1.314	8.33E-09	1.02E-05	-1.343	2.65E-09	3.36E-06
ENSG00000180537	*RNF182*	-1.477	1.46E-09	2.31E-06	-1.441	3.09E-09	3.86E-06
ENSG00000238741	*SCARNA7*	-1.271	4.65E-09	6.71E-06	-1.278	3.70E-09	4.53E-06
ENSG00000111145	*ELK3*	-1.266	4.78E-09	6.73E-06	-1.269	4.36E-09	5.16E-06
ENSG00000071967	*CYBRD1*	-1.313	5.37E-09	7.25E-06	-1.313	5.18E-09	5.97E-06
ENSG00000111799	*COL12A1*	-1.271	5.79E-09	7.66E-06	-1.259	5.25E-09	5.97E-06
ENSG00000138777	*PPA2*	1.134	2.66E-07	0.00018	1.263	5.87E-09	6.57E-06

ENSG00000147606	*SLC26A7*	1.736	2.46E-09	3.80E-06	1.619	6.26E-09	6.88E-06
ENSG00000179388	*EGR3*	-1.665	9.11E-09	1.09E-05	-1.673	6.99E-09	7.56E-06
ENSG00000224715	*LOC339685*	1.638	2.76E-08	2.84E-05	1.682	8.87E-09	9.28E-06
ENSG00000196154	*S100A4*	1.367	8.84E-08	7.07E-05	1.444	9.53E-09	9.81E-06
ENSG00000248323	*LUCAT1*	1.263	8.37E-09	1.02E-05	1.253	1.00E-08	1.01E-05
ENSG00000084234	*APLP2*	-1.180	1.57E-08	1.75E-05	-1.194	1.02E-08	1.02E-05
ENSG00000175793	*SFN*	1.194	1.19E-08	1.41E-05	1.194	1.18E-08	1.16E-05
ENSG00000106462	*EZH2*	1.090	2.47E-07	0.00017	1.194	1.45E-08	1.40E-05
ENSG00000145246	*ATP10D*	-1.332	1.31E-08	1.51E-05	-1.314	1.48E-08	1.42E-05
ENSG00000189143	*CLDN4*	1.200	1.66E-08	1.77E-05	1.196	1.84E-08	1.73E-05
ENSG00000171421	*MRPL36*	-1.268	2.12E-08	2.21E-05	-1.255	2.25E-08	2.08E-05
ENSG00000229261	*LOC101928994*	1.551	2.86E-08	2.85E-05	1.535	2.39E-08	2.18E-05
ENSG00000074047	*GLI2*	-1.435	5.91E-09	7.66E-06	-1.350	2.42E-08	2.18E-05
ENSG00000047597	*XK*	-1.190	3.57E-08	3.46E-05	-1.193	3.19E-08	2.79E-05
ENSG00000221890	*NPTXR*	-1.293	3.30E-08	3.24E-05	-1.279	3.74E-08	3.23E-05
ENSG00000159640	*ACE*	-1.690	3.71E-07	0.00023	-1.730	4.60E-08	3.87E-05
ENSG00000254911	*SCARNA9*	-1.404	1.49E-07	0.00011	-1.407	5.67E-08	4.72E-05
ENSG00000115884	*SDC1*	-1.212	4.89E-08	4.48E-05	-1.201	5.80E-08	4.76E-05
ENSG00000184321	*OR51J1*	1.325	7.92E-08	6.60E-05	1.331	6.25E-08	5.07E-05
ENSG00000115540	*MOB4*	-1.239	2.82E-08	2.85E-05	-1.205	6.43E-08	5.15E-05
ENSG00000172986	*GXYLT2*	-1.473	4.23E-07	0.00026	-1.533	6.64E-08	5.25E-05
ENSG00000130522	*JUND*	1.134	7.72E-08	6.59E-05	1.137	7.02E-08	5.48E-05
ENSG00000160199	*PKNOX1*	-1.156	1.02E-07	8.07E-05	-1.161	7.52E-08	5.81E-05
ENSG00000126838	*PZP*	1.869	7.73E-08	6.59E-05	1.846	8.49E-08	6.47E-05
ENSG00000073792	*IGF2BP2*	-1.081	2.64E-07	0.00018	-1.114	1.05E-07	7.88E-05
ENSG00000125827	*TMX4*	-1.139	2.43E-07	0.00017	-1.165	1.08E-07	7.97E-05
ENSG00000053747	*LAMA3*	1.286	6.06E-08	5.39E-05	1.224	1.09E-07	7.97E-05
ENSG00000025039	*RRAGD*	-1.209	7.94E-08	6.60E-05	-1.183	1.19E-07	8.59E-05
ENSG00000101265	*RASSF2*	-1.902	9.16E-07	0.00050	-1.990	1.38E-07	9.52E-05
ENSG00000162909	*CAPN2*	-1.052	4.31E-07	0.00027	-1.094	1.43E-07	9.76E-05
ENSG00000156042	*CFAP70*	1.226	3.88E-08	3.65E-05	1.162	1.46E-07	9.76E-05
ENSG00000156966	*B3GNT7*	-1.939	2.04E-07	0.00015	-1.915	1.46E-07	9.76E-05
ENSG00000165424	*ZCCHC24*	-1.352	3.03E-07	0.00020	-1.380	1.58E-07	0.000105
ENSG00000104154	*SLC30A4*	-1.329	8.17E-08	6.71E-05	-1.291	1.67E-07	0.000110
ENSG00000110917	*MLEC*	-1.099	1.43E-07	0.00011	-1.091	1.71E-07	0.000111
ENSG00000114115	*RBP1*	-1.089	3.28E-07	0.00021	-1.109	1.77E-07	0.000114
ENSG00000173218	*VANGL1*	-1.133	1.14E-07	8.93E-05	-1.110	1.91E-07	0.000121
ENSG00000111319	*SCNN1A*	1.226	7.19E-08	6.30E-05	1.163	2.46E-07	0.000155
ENSG00000177542	*SLC25A22*	-1.089	5.96E-07	0.00036	-1.115	2.74E-07	0.000171
ENSG00000137501	*SYTL2*	-1.117	3.71E-07	0.00023	-1.121	2.87E-07	0.000177
ENSG00000197594	*ENPP1*	-1.067	2.28E-06	0.00104	-1.143	2.94E-07	0.000180
ENSG00000180758	*GPR157*	-1.216	3.24E-07	0.00021	-1.211	3.07E-07	0.000186
ENSG00000179750	*APOBEC3B*	1.318	8.41E-08	6.82E-05	1.180	3.46E-07	0.000206
ENSG00000148248	*SURF4*	-1.082	3.51E-07	0.00023	-1.062	3.83E-07	0.000224
ENSG00000099864	*PALM*	-1.224	1.83E-07	0.00014	-1.172	3.92E-07	0.000227
ENSG00000145244	*CORIN*	1.228	2.00E-07	0.00015	1.169	4.21E-07	0.000241
ENSG00000203760	*CENPW*	-1.123	1.07E-06	0.00056	-1.148	4.24E-07	0.000241
ENSG00000175556	*LONRF3*	-1.137	9.87E-07	0.00053	-1.156	4.47E-07	0.000252
ENSG00000235703	*LINC00894*	1.117	5.82E-06	0.00216	1.198	4.59E-07	0.000256
ENSG00000181458	*TMEM45A*	-1.432	2.20E-07	0.00016	-1.363	5.03E-07	0.000279
ENSG00000109171	*SLAIN2*	-1.038	1.14E-06	0.00059	-1.068	5.24E-07	0.000288
ENSG00000184743	*ATL3*	-1.089	3.46E-07	0.00022	-1.068	5.30E-07	0.000289
ENSG00000120708	*TGFBI*	3.487	8.97E-07	0.00050	3.526	5.49E-07	0.000297
ENSG00000112175	*BMP5*	2.751	3.15E-06	0.00133	2.884	5.63E-07	0.000302
ENSG00000174130	*TLR6*	1.299	3.05E-07	0.00020	1.251	5.94E-07	0.000311
ENSG00000230726	*HLA-DRA*	-3.507	0.00250	0.16464	-2.058	6.04E-07	0.000311

ENSG00000237686	*LOC101929705*	1.324	1.99E-06	0.00094	1.385	6.64E-07	0.000338
ENSG00000163840	*DTX3L*	-1.079	8.46E-07	0.00048	-1.086	6.67E-07	0.000338
ENSG00000285280	*LOC105371664*	1.184	8.03E-07	0.00046	1.179	6.88E-07	0.000346
ENSG00000139318	*DUSP6*	-1.067	9.98E-07	0.00053	-1.081	7.01E-07	0.000350
ENSG00000163820	*FYCO1*	-1.041	1.13E-06	0.00059	-1.052	8.08E-07	0.000398
ENSG00000215529	*EFCAB8*	2.161	2.62E-06	0.00116	2.142	8.87E-07	0.000425
ENSG00000163754	*GYG1*	-1.051	1.20E-06	0.00061	-1.058	8.91E-07	0.000425
ENSG00000152952	*PLOD2*	-1.031	1.80E-06	0.00086	-1.050	8.92E-07	0.000425
ENSG00000229240	*LINC00710*	1.299	8.36E-07	0.00048	1.299	9.51E-07	0.000450
ENSG00000225855	*RUSC1-AS1*	1.164	6.31E-07	0.00037	1.140	1.08E-06	0.000508
ENSG00000079215	*SLC1A3*	-1.083	8.66E-07	0.00048	-1.060	1.25E-06	0.000577
ENSG00000206450	*HLA-B*	-1.044	1.44E-05	0.00443	-1.043	1.26E-06	0.000577
ENSG00000164406	*LEAP2*	1.280	2.65E-06	0.00116	1.311	1.26E-06	0.000577
ENSG00000075420	*FNDC3B*	-1.024	1.40E-06	0.00069	-1.021	1.33E-06	0.000604
ENSG00000001084	*GCLC*	1.011	1.29E-06	0.00065	1.007	1.35E-06	0.000610
ENSG00000147124	*ZNF41*	-1.041	3.02E-06	0.00128	-1.072	1.47E-06	0.000653
ENSG00000166801	*FAM111A*	-1.258	2.65E-06	0.00116	-1.276	1.48E-06	0.000653
ENSG00000101955	*SRPX*	-1.360	1.46E-06	0.00072	-1.273	1.48E-06	0.000653
ENSG00000165704	*HPRT1*	-0.993	5.42E-06	0.00204	-1.033	1.49E-06	0.000653
ENSG00000183091	*NEB*	1.018	4.79E-06	0.00183	1.054	1.58E-06	0.000689
ENSG00000228163	*HLA-DPA1*	-3.507	0.00250	0.16464	-1.774	1.61E-06	0.000691
ENSG00000106789	*CORO2A*	-1.319	5.49E-07	0.00034	-1.232	1.74E-06	0.000731
ENSG00000167972	*ABCA3*	-1.186	5.38E-06	0.00204	-1.208	1.76E-06	0.000736
ENSG00000138756	*BMP2K*	-1.016	6.09E-06	0.00223	-1.053	1.89E-06	0.000780
ENSG00000160131	*VMA21*	-1.008	1.76E-06	0.00085	-1.004	1.91E-06	0.000780
ENSG00000242290	*ZBTB20-AS5*	1.594	2.42E-06	0.00109	1.604	1.93E-06	0.000780
ENSG00000177494	*ZBED2*	-1.036	2.01E-06	0.00094	-1.037	1.94E-06	0.000780
ENSG00000150961	*SEC24D*	-0.962	7.24E-06	0.00254	-1.007	2.22E-06	0.000881
ENSG00000168785	*TSPAN5*	-1.014	1.92E-06	0.00091	-1.004	2.24E-06	0.000881
ENSG00000168077	*SCARA3*	-0.964	6.55E-06	0.00236	-1.008	2.31E-06	0.000901
ENSG00000117394	*SLC2A1*	-0.970	3.31E-06	0.00139	-0.983	2.38E-06	0.000922
ENSG00000137440	*FGFBP1*	1.083	1.38E-06	0.00069	1.057	2.41E-06	0.000922
ENSG00000123989	*CHPF*	-0.973	3.55E-06	0.00146	-0.989	2.44E-06	0.000922
ENSG00000176399	*DMRTA1*	1.467	3.58E-06	0.00146	1.490	2.46E-06	0.000922
ENSG00000133216	*EPHB2*	-1.061	5.92E-06	0.00218	-1.095	2.48E-06	0.000922
ENSG00000023445	*BIRC3*	1.195	1.19E-06	0.00061	1.145	2.57E-06	0.000936
ENSG00000144410	*CPO*	3.687	0.00141	0.11374	4.515	2.75E-06	0.000998
ENSG00000164983	*TMEM65*	-1.061	2.87E-06	0.00123	-1.050	3.23E-06	0.001143
ENSG00000139044	*B4GALNT3*	-1.232	1.74E-06	0.00085	-1.168	3.29E-06	0.001161
ENSG00000137767	*SQOR*	-1.365	3.38E-06	0.00141	-1.303	3.44E-06	0.001206
ENSG00000106785	*TRIM14*	-0.984	3.68E-06	0.00149	-0.984	3.47E-06	0.001209
ENSG00000116983	*HPCAL4*	-1.171	5.74E-06	0.00215	-1.192	3.49E-06	0.001209
ENSG00000166762	*CATSPER2*	1.042	1.21E-05	0.00392	1.071	3.55E-06	0.001225
ENSG00000105976	*MET*	-0.977	2.82E-06	0.00122	-0.965	3.61E-06	0.001236
ENSG00000184602	*SNN*	-1.082	4.09E-06	0.00162	-1.080	3.81E-06	0.001295
ENSG00000167360	*OR51Q1*	1.089	3.76E-06	0.00151	1.085	3.95E-06	0.001335
ENSG00000131969	*ABHD12B*	1.165	5.81E-06	0.00216	1.181	4.28E-06	0.001432
ENSG00000173114	*LRRN3*	1.254	4.37E-06	0.00168	1.251	4.37E-06	0.001452
ENSG00000176438	*SYNE3*	-1.253	6.70E-06	0.00239	-1.258	4.41E-06	0.001459
ENSG00000161921	*CXCL16*	-1.053	4.22E-06	0.00166	-1.035	4.51E-06	0.001483
ENSG00000183762	*KREMEN1*	-0.973	1.85E-05	0.00533	-1.032	4.95E-06	0.001612
ENSG00000135318	*NT5E*	-1.004	4.32E-06	0.00168	-0.990	5.19E-06	0.001673
ENSG00000172901	*LVRN*	1.587	1.09E-05	0.00360	1.573	5.65E-06	0.001813
ENSG00000163644	*PPM1K*	-1.076	1.06E-05	0.00352	-1.098	5.72E-06	0.001826
ENSG00000066629	*EML1*	-1.173	1.77E-06	0.00085	-1.071	5.96E-06	0.001894
ENSG00000106799	*TGFBR1*	-0.964	6.35E-06	0.00230	-0.965	6.02E-06	0.001905

ENSG00000128965	*CHAC1*	1.451	2.26E-05	0.00623	1.493	6.21E-06	0.001955
ENSG00000196754	*S100A2*	1.206	2.60E-06	0.00116	1.136	6.25E-06	0.001956
ENSG00000109472	*CPE*	-0.871	6.60E-05	0.01407	-0.977	6.32E-06	0.001969
ENSG00000159899	*NPR2*	1.072	1.16E-05	0.00378	1.080	6.36E-06	0.001975
ENSG00000099954	*CECR2*	-0.943	4.44E-05	0.01053	-1.032	6.62E-06	0.002033
ENSG00000271303	*SRXN1*	1.168	6.60E-06	0.00236	1.166	6.65E-06	0.002033
ENSG00000157851	*DPYSL5*	-1.049	7.41E-06	0.00258	-1.031	7.44E-06	0.002244
ENSG00000155876	*RRAGA*	-0.969	8.26E-06	0.00282	-0.974	7.44E-06	0.002244
ENSG00000153214	*TMEM87B*	-1.023	7.78E-06	0.00268	-1.013	7.57E-06	0.002272
ENSG00000178297	*TMPRSS9*	1.221	1.29E-05	0.00411	1.248	7.60E-06	0.002272
ENSG00000138821	*SLC39A8*	-0.958	8.25E-06	0.00282	-0.954	7.88E-06	0.002343
ENSG00000138814	*PPP3CA*	-0.985	7.00E-06	0.00248	-0.969	8.19E-06	0.002424
ENSG00000119125	*GDA*	-1.247	3.84E-06	0.00153	-1.166	8.24E-06	0.002427
ENSG00000187003	*ACTL7A*	7.277	8.35E-06	0.00283	7.275	8.35E-06	0.002444
ENSG00000168938	*PPIC*	-0.979	1.92E-05	0.00550	-1.010	8.37E-06	0.002444
ENSG00000171208	*NETO2*	-1.028	6.16E-06	0.00225	-1.001	8.55E-06	0.002487
ENSG00000149131	*SERPING1*	-3.131	1.73E-05	0.00511	-2.741	8.72E-06	0.002525
ENSG00000168243	*GNG4*	-1.048	0.00055	0.06365	-1.145	9.50E-06	0.002737
ENSG00000095209	*TMEM38B*	-0.976	1.25E-05	0.00403	-0.979	9.62E-06	0.002760
ENSG00000237975	*FLG-AS1*	3.208	1.73E-05	0.00511	2.997	9.87E-06	0.002818
ENSG00000272636	*DOC2B*	-1.119	2.65E-05	0.00697	-1.032	1.02E-05	0.002915
ENSG00000168993	*CPLX1*	-1.073	1.83E-05	0.00532	-1.092	1.11E-05	0.003140
ENSG00000196236	*XPNPEP3*	0.908	1.37E-05	0.00428	0.916	1.13E-05	0.003186
ENSG00000101384	*JAG1*	-0.934	2.36E-05	0.00645	-0.959	1.14E-05	0.003186
ENSG00000073670	*ADAM11*	-1.302	6.46E-07	0.00037	-1.095	1.15E-05	0.003196
ENSG00000168994	*PXDC1*	-1.806	1.43E-05	0.00443	-1.765	1.16E-05	0.003196
ENSG00000120306	*CYSTM1*	-0.878	0.00010	0.01970	-0.979	1.16E-05	0.003196
ENSG00000241769	*LINC00893*	2.238	0.00054	0.06297	2.149	1.20E-05	0.003282
ENSG00000277518	*MUC6*	1.892	0.00644	0.28908	1.741	1.24E-05	0.003377
ENSG00000164309	*CMYA5*	1.152	4.72E-06	0.00181	1.091	1.28E-05	0.003461
ENSG00000214944	*ARHGEF28*	-1.014	1.84E-05	0.00532	-1.010	1.37E-05	0.003678
ENSG00000134590	*RTL8C*	-0.960	2.92E-05	0.00755	-0.996	1.42E-05	0.003794
ENSG00000151135	*TMEM263*	-0.924	1.53E-05	0.00460	-0.926	1.42E-05	0.003800
ENSG00000164749	*HNF4G*	-1.118	2.60E-05	0.00689	-1.128	1.46E-05	0.003880
ENSG00000175414	*ARL10*	1.294	1.77E-05	0.00519	1.297	1.48E-05	0.003889
ENSG00000124733	*MEA1*	-0.873	5.75E-05	0.01282	-0.934	1.48E-05	0.003889
ENSG00000138757	*G3BP2*	-0.898	1.51E-05	0.00457	-0.898	1.49E-05	0.003889
ENSG00000113328	*CCNG1*	-0.896	2.54E-05	0.00676	-0.918	1.51E-05	0.003931
ENSG00000066117	*SMARCD1*	-0.889	2.40E-05	0.00655	-0.907	1.53E-05	0.003972
ENSG00000112294	*ALDH5A1*	-0.956	3.02E-05	0.00771	-0.986	1.55E-05	0.003996
ENSG00000149218	*ENDOD1*	-0.920	1.68E-05	0.00498	-0.919	1.67E-05	0.004302
ENSG00000244617	*ASPRV1*	1.878	3.21E-05	0.00809	1.928	1.68E-05	0.004302
ENSG00000136842	*TMOD1*	-1.198	2.05E-05	0.00579	-1.152	1.70E-05	0.004327
ENSG00000184076	*UQCR10*	-0.887	5.19E-05	0.01189	-0.935	1.71E-05	0.004327
ENSG00000138175	*ARL3*	-0.861	0.00015	0.02497	-0.956	1.71E-05	0.004327
ENSG00000184698	*OR51M1*	0.956	1.02E-05	0.00340	0.932	1.73E-05	0.004357
ENSG00000086619	*ERO1B*	-1.026	0.00023	0.03313	-1.156	1.75E-05	0.004360
ENSG00000103855	*CD276*	-0.929	1.28E-05	0.00411	-0.908	1.75E-05	0.004360
ENSG00000140092	*FBLN5*	-1.923	4.05E-05	0.00985	-1.863	1.77E-05	0.004409
ENSG00000206306	*HLA-DRB1*	-1.857	0.00015	0.02518	-1.315	1.79E-05	0.004414
ENSG00000126231	*PROZ*	1.495	1.16E-05	0.00378	1.453	1.80E-05	0.004430
ENSG00000198695	*ND6*	0.894	1.48E-05	0.00450	0.884	1.81E-05	0.004437
ENSG00000100167	*SEPTIN3*	-1.007	0.00012	0.02118	-1.107	1.87E-05	0.004563
ENSG00000156587	*UBE2L6*	-1.170	1.99E-05	0.00567	-1.153	2.02E-05	0.004907
ENSG00000151689	*INPP1*	-0.989	2.04E-05	0.00579	-0.973	2.04E-05	0.004919
ENSG00000085377	*PREP*	-0.898	2.48E-05	0.00665	-0.898	2.16E-05	0.005193

ENSG00000133083	*DCLK1*	-1.198	6.99E-05	0.01481	-1.253	2.17E-05	0.005198
ENSG00000135002	*RFK*	-0.925	2.14E-05	0.00596	-0.922	2.19E-05	0.005216
ENSG00000172794	*RAB37*	-1.585	8.61E-05	0.01746	-1.645	2.20E-05	0.005216
ENSG00000136026	*CKAP4*	-0.861	4.29E-05	0.01034	-0.892	2.21E-05	0.005232
ENSG00000251562	*MALAT1*	0.877	2.05E-05	0.00579	0.872	2.26E-05	0.005336
ENSG00000165730	*STOX1*	-0.942	6.32E-05	0.01380	-0.993	2.27E-05	0.005336
ENSG00000156298	*TSPAN7*	-1.542	2.29E-05	0.00630	-1.498	2.29E-05	0.005368
ENSG00000116704	*SLC35D1*	-0.939	1.79E-05	0.00523	-0.923	2.30E-05	0.005372
ENSG00000158104	*HPD*	1.236	0.00018	0.02799	1.333	2.37E-05	0.005515
ENSG00000130783	*CCDC62*	0.975	3.19E-05	0.00807	0.974	2.45E-05	0.005673
ENSG00000138496	*PARP9*	-0.906	0.00012	0.02110	-0.982	2.47E-05	0.005703
ENSG00000124159	*MATN4*	-2.719	0.00056	0.06408	-2.833	2.53E-05	0.005792
ENSG00000177606	*JUN*	0.891	2.14E-05	0.00596	0.882	2.55E-05	0.005811
ENSG00000108100	*CCNY*	-0.854	5.29E-05	0.01203	-0.885	2.59E-05	0.005869
ENSG00000172031	*EPHX4*	-1.462	5.75E-05	0.01282	-1.407	2.64E-05	0.005961
ENSG00000205363	*INSYN1*	-1.348	9.62E-05	0.01889	-1.427	2.70E-05	0.006083
ENSG00000168685	*IL7R*	-1.395	0.00014	0.02408	-1.482	2.90E-05	0.006493
ENSG00000008441	*NFIX*	-0.907	2.92E-05	0.00755	-0.903	2.90E-05	0.006493
ENSG00000197093	*GAL3ST4*	1.535	3.73E-05	0.00930	1.535	2.92E-05	0.006496
ENSG00000181609	*OR52D1*	0.925	2.44E-05	0.00663	0.915	3.03E-05	0.006720
ENSG00000136490	*LIMD2*	-1.030	0.00011	0.02013	-1.092	3.04E-05	0.006720
ENSG00000187918	*OR51I2*	0.899	2.95E-05	0.00755	0.896	3.08E-05	0.006797
ENSG00000005893	*LAMP2*	-0.908	2.72E-05	0.00714	-0.894	3.13E-05	0.006884
ENSG00000073849	*ST6GAL1*	-0.878	0.00010	0.01970	-0.935	3.26E-05	0.007138
ENSG00000198648	*STK39*	-0.901	2.47E-05	0.00665	-0.877	3.31E-05	0.007234
ENSG00000169783	*LINGO1*	-0.977	2.85E-05	0.00742	-0.969	3.33E-05	0.007234
ENSG00000166401	*SERPINB8*	-1.008	4.35E-05	0.01040	-1.008	3.34E-05	0.007246
ENSG00000213626	*LBH*	-2.877	1.58E-05	0.00472	-2.613	3.36E-05	0.007246
ENSG00000188897	*LOC400499*	0.873	7.07E-05	0.01488	0.904	3.36E-05	0.007246
ENSG00000099953	*MMP11*	-1.058	0.00205	0.14440	-1.126	3.49E-05	0.007444
ENSG00000223757	*VWA7*	2.119	0.00057	0.06545	1.108	3.53E-05	0.007444
ENSG00000183087	*GAS6*	-0.854	0.00013	0.02233	-0.917	3.57E-05	0.007484
ENSG00000178809	*TRIM73*	0.888	0.00059	0.06604	0.955	3.59E-05	0.007512
ENSG00000157613	*CREB3L1*	-1.396	0.00077	0.07986	-1.653	3.72E-05	0.007740
ENSG00000111962	*UST*	-1.060	0.00011	0.02024	-1.106	3.72E-05	0.007740
ENSG00000089327	*FXYD5*	-0.842	0.00031	0.04052	-0.936	3.75E-05	0.007741
ENSG00000089472	*HEPH*	-1.527	1.45E-05	0.00443	-1.355	3.75E-05	0.007741
ENSG00000138641	*HERC3*	-0.997	2.22E-05	0.00615	-0.949	3.77E-05	0.007753
ENSG00000128573	*FOXP2*	-1.390	2.09E-05	0.00586	-1.295	3.94E-05	0.008090
ENSG00000188042	*ARL4C*	1.442	1.44E-05	0.00443	1.355	3.96E-05	0.008090
ENSG00000167359	*OR51I1*	0.959	4.42E-05	0.01053	0.965	4.08E-05	0.008318
ENSG00000144730	*IL17RD*	-1.051	7.62E-06	0.00264	-0.953	4.14E-05	0.008417
ENSG00000128242	*GAL3ST1*	-1.305	5.16E-05	0.01189	-1.314	4.19E-05	0.008495
ENSG00000233101	*HOXB-AS3*	1.134	3.42E-05	0.00860	1.123	4.26E-05	0.008583
ENSG00000152503	*TRIM36*	-0.831	0.00026	0.03641	-0.924	4.33E-05	0.008674
ENSG00000091490	*SEL1L3*	-0.875	4.83E-05	0.01127	-0.870	4.37E-05	0.008727
ENSG00000176239	*OR51B6*	0.999	6.15E-05	0.01356	1.013	4.48E-05	0.008916
ENSG00000116852	*KIF21B*	-0.935	1.00E-04	0.01935	-0.964	4.52E-05	0.008964
ENSG00000203499	*FAM83H-AS1*	0.852	0.00011	0.02100	0.867	4.60E-05	0.009083
ENSG00000227801	*COL11A2*	1.102	0.00267	0.17166	0.945	4.80E-05	0.009366
ENSG00000023171	*GRAMD1B*	0.969	0.00013	0.02209	1.009	4.85E-05	0.009448
ENSG00000116745	*RPE65*	0.903	4.52E-05	0.01069	0.900	4.93E-05	0.009533
ENSG00000080166	*DCT*	-1.607	6.52E-05	0.01403	-1.564	4.93E-05	0.009533
ENSG00000179674	*ARL14*	1.640	4.94E-05	0.01144	1.637	4.94E-05	0.009533
ENSG00000071282	*LMCD1*	-1.041	0.00027	0.03698	-1.123	5.15E-05	0.009889
ENSG00000183696	*UPP1*	0.851	5.18E-05	0.01189	0.847	5.21E-05	0.009962

ENSG00000113300	*CNOT6*	0.837	7.30E-05	0.01526	0.851	5.28E-05	0.010071
ENSG00000112599	*GUCA1B*	1.129	4.09E-05	0.00991	1.091	5.34E-05	0.010146
ENSG00000081803	*CADPS2*	-0.862	0.00016	0.02585	-0.901	5.36E-05	0.010155
ENSG00000073150	*PANX2*	-0.975	5.71E-05	0.01280	-0.974	5.37E-05	0.010155
ENSG00000185650	*ZFP36L1*	-0.834	5.59E-05	0.01264	-0.836	5.44E-05	0.010253
ENSG00000257218	*GATC*	0.853	7.53E-05	0.01566	0.869	5.47E-05	0.010281
ENSG00000273033	*LINC02035*	-1.032	8.44E-05	0.01738	-1.058	5.55E-05	0.010391
ENSG00000154511	*DIPK1A*	-1.024	3.91E-05	0.00958	-0.994	5.76E-05	0.010650
ENSG00000128805	*ARHGAP22*	-0.989	0.00015	0.02513	-1.033	5.77E-05	0.010650
ENSG00000151881	*TMEM267*	-0.898	0.00014	0.02366	-0.940	5.87E-05	0.010768
ENSG00000127364	*TAS2R4*	1.005	0.00010	0.01970	1.035	5.88E-05	0.010768
ENSG00000169251	*NMD3*	-0.800	0.00014	0.02355	-0.839	5.91E-05	0.010768
ENSG00000179532	*DNHD1*	0.883	3.91E-05	0.00958	0.859	5.91E-05	0.010768
ENSG00000167524	*RSKR*	0.888	0.00010	0.01959	0.914	6.20E-05	0.011171
ENSG00000166311	*SMPD1*	-1.042	0.00012	0.02171	-1.060	6.26E-05	0.011250
ENSG00000080561	*MID2*	-1.104	0.00011	0.02041	-1.105	6.33E-05	0.011342
ENSG00000169213	*RAB3B*	-0.857	6.71E-05	0.01427	-0.858	6.41E-05	0.011455
ENSG00000198959	*TGM2*	-1.378	0.00021	0.03041	-1.391	6.48E-05	0.011524
ENSG00000080503	*SMARCA2*	-0.867	0.00022	0.03153	-0.921	6.56E-05	0.011597
ENSG00000198682	*PAPSS2*	-0.926	0.00013	0.02320	-0.957	6.73E-05	0.011825
ENSG00000150938	*CRIM1*	-0.810	0.00013	0.02306	-0.841	6.73E-05	0.011825
ENSG00000139926	*FRMD6*	-0.912	0.00016	0.02584	-0.944	6.80E-05	0.011915
ENSG00000258955	*LINC00519*	1.366	8.65E-05	0.01746	1.379	6.93E-05	0.012110
ENSG00000137198	*GMPR*	-0.897	0.00098	0.09257	-1.009	6.97E-05	0.012153
ENSG00000188015	*S100A3*	1.352	3.89E-05	0.00958	1.260	7.08E-05	0.012313
ENSG00000147592	*LACTB2*	-0.861	0.00011	0.02013	-0.874	7.19E-05	0.012427
ENSG00000138759	*FRAS1*	-0.831	8.64E-05	0.01746	-0.836	7.23E-05	0.012441
ENSG00000186115	*CYP4F2*	2.332	0.00076	0.07937	2.024	7.28E-05	0.012495
ENSG00000206435	*HLA-C*	-0.882	0.00015	0.02480	-0.844	7.37E-05	0.012573
ENSG00000151348	*EXT2*	-0.830	8.47E-05	0.01738	-0.833	7.50E-05	0.012694
ENSG00000166710	*B2M*	-0.861	4.82E-05	0.01127	-0.825	7.67E-05	0.012921
ENSG00000135048	*CEMIP2*	-0.867	0.00013	0.02277	-0.885	7.70E-05	0.012934
ENSG00000005249	*PRKAR2B*	-0.915	0.00011	0.02100	-0.884	7.82E-05	0.013105
ENSG00000104267	*CA2*	-0.826	0.00013	0.02277	-0.844	7.88E-05	0.013163
ENSG00000135108	*FBXO21*	-0.807	0.00014	0.02376	-0.834	7.90E-05	0.013163
ENSG00000155016	*CYP2U1*	-0.946	8.76E-05	0.01759	-0.949	8.11E-05	0.013382
ENSG00000279078	*SND1-IT1*	1.052	8.59E-05	0.01746	1.063	8.19E-05	0.013469
ENSG00000184923	*NUTM2A*	1.039	7.21E-05	0.01513	0.882	8.34E-05	0.013691
ENSG00000165076	*PRSS37*	2.192	0.00012	0.02207	2.228	8.39E-05	0.013737
ENSG00000013619	*MAMLD1*	-0.931	0.00034	0.04411	-1.012	8.51E-05	0.013891
ENSG00000141424	*SLC39A6*	-0.820	7.86E-05	0.01628	-0.815	8.56E-05	0.013891
ENSG00000282164	*PEG13*	1.254	0.00015	0.02525	1.294	8.59E-05	0.013891
ENSG00000140941	*MAP1LC3B*	-0.858	4.94E-05	0.01144	-0.826	8.63E-05	0.013891
ENSG00000124116	*WFDC3*	1.808	0.00011	0.02100	1.723	8.63E-05	0.013891
ENSG00000049249	*TNFRSF9*	1.384	9.09E-05	0.01814	1.363	8.65E-05	0.013891
ENSG00000102531	*FNDC3A*	-0.790	0.00022	0.03153	-0.833	8.66E-05	0.013891
ENSG00000185674	*LYG2*	1.594	0.00015	0.02513	1.639	8.66E-05	0.013891
ENSG00000115353	*TACR1*	1.695	8.68E-05	0.01747	1.692	8.68E-05	0.013891
ENSG00000151364	*KCTD14*	-1.107	0.00012	0.02111	-1.128	8.82E-05	0.014092
ENSG00000158286	*RNF207*	0.857	7.05E-05	0.01488	0.841	9.06E-05	0.014433
ENSG00000102580	*DNAJC3*	-0.796	0.00015	0.02437	-0.818	9.29E-05	0.014770
ENSG00000157227	*MMP14*	1.038	9.27E-05	0.01837	1.017	9.54E-05	0.015130
ENSG00000169914	*OTUD3*	0.819	0.00012	0.02171	0.829	9.57E-05	0.015130
ENSG00000135404	*CD63*	-0.797	0.00017	0.02740	-0.822	9.62E-05	0.015178
ENSG00000151729	*SLC25A4*	-0.847	0.00013	0.02209	-0.853	9.68E-05	0.015236
ENSG00000152413	*HOMER1*	-0.829	0.00010	0.01970	-0.826	0.00010	0.015935

ENSG00000143162	*CREG1*	-0.809	0.00019	0.02853	-0.837	0.00010	0.015980
ENSG00000188511	*C22orf34*	0.888	0.00022	0.03164	0.925	0.00010	0.016082
ENSG00000135363	*LMO2*	-2.568	0.00026	0.03641	-2.682	0.00010	0.016109
ENSG00000166016	*ABTB2*	0.867	0.00011	0.02005	0.859	0.00011	0.016347
ENSG00000085063	*CD59*	-0.852	8.50E-05	0.01738	-0.838	0.00011	0.016452
ENSG00000153071	*DAB2*	-0.938	0.00014	0.02366	-0.944	0.00011	0.016484
ENSG00000149050	*ZNF214*	1.836	0.00031	0.04005	1.932	0.00011	0.016678
ENSG00000214562	*NUTM2D*	0.679	0.00814	0.32767	0.861	0.00011	0.016696
ENSG00000108813	*DLX4*	1.163	6.43E-05	0.01399	1.107	0.00011	0.016744
ENSG00000246922	*UBAP1L*	0.858	0.00011	0.02045	0.857	0.00011	0.016744
ENSG00000187164	*SHTN1*	0.799	0.00013	0.02210	0.804	0.00011	0.016753
ENSG00000197635	*DPP4*	-1.128	0.00111	0.10055	-1.220	0.00011	0.016753
ENSG00000163053	*SLC16A14*	-0.838	0.00025	0.03560	-0.879	0.00011	0.017161
ENSG00000254206	*NPIPB11*	0.646	0.00571	0.26897	0.821	0.00011	0.017231
ENSG00000142449	*FBN3*	-1.107	0.00079	0.08089	-1.192	0.00012	0.017262
ENSG00000135905	*DOCK10*	1.709	0.00022	0.03164	1.729	0.00012	0.017479
ENSG00000120742	*SERP1*	-0.818	9.09E-05	0.01814	-0.804	0.00012	0.017596
ENSG00000065320	*NTN1*	-1.033	6.47E-05	0.01400	-0.991	0.00012	0.017917
ENSG00000154310	*TNIK*	-0.803	0.00027	0.03680	-0.834	0.00012	0.018127
ENSG00000163162	*RNF149*	-0.770	0.00029	0.03924	-0.815	0.00012	0.018127
ENSG00000060762	*MPC1*	-0.943	0.00012	0.02202	-0.922	0.00012	0.018367
ENSG00000188199	*NUTM2B*	0.844	0.00130	0.10868	0.859	0.00013	0.018763
ENSG00000185269	*NOTUM*	-0.889	0.00120	0.10514	-1.020	0.00013	0.018880
ENSG00000123892	*RAB38*	-1.106	0.00015	0.02505	-1.100	0.00013	0.018894
ENSG00000069869	*NEDD4*	-0.865	0.00010	0.01970	-0.839	0.00013	0.018894
ENSG00000166436	*TRIM66*	0.839	9.95E-05	0.01931	0.820	0.00013	0.019593
ENSG00000003989	*SLC7A2*	-0.801	0.00016	0.02623	-0.809	0.00014	0.020020
ENSG00000110092	*CCND1*	0.790	0.00013	0.02229	0.786	0.00014	0.020043
ENSG00000143061	*IGSF3*	-0.817	0.00016	0.02564	-0.815	0.00014	0.020173
ENSG00000144369	*FAM171B*	-0.868	9.24E-05	0.01837	-0.839	0.00014	0.020409
ENSG00000025772	*TOMM34*	-0.767	0.00030	0.03945	-0.803	0.00014	0.020414
ENSG00000259207	*ITGB3*	2.026	0.00014	0.02408	2.022	0.00014	0.020414
ENSG00000160678	*S100A1*	1.421	7.54E-05	0.01566	1.390	0.00014	0.020434
ENSG00000137962	*ARHGAP29*	-0.770	0.00019	0.02878	-0.783	0.00015	0.021042
ENSG00000104361	*NIPAL2*	-0.968	6.54E-05	0.01403	-0.906	0.00015	0.021042
ENSG00000266412	*NCOA4*	-0.778	0.00019	0.02873	-0.789	0.00015	0.021159
ENSG00000277254	*TAS2R13*	2.816	8.72E-06	0.00295	1.411	0.00015	0.021159
ENSG00000231683	*LOC101927136*	1.358	0.00031	0.04005	1.411	0.00015	0.021159
ENSG00000182796	*TMEM198B*	1.074	0.00018	0.02750	1.079	0.00015	0.021324
ENSG00000162745	*OLFML2B*	-1.681	0.00016	0.02574	-1.592	0.00016	0.021431
ENSG00000112874	*NUDT12*	-0.828	0.00028	0.03757	-0.849	0.00016	0.021558
ENSG00000179818	*PCBP1-AS1*	0.876	5.61E-05	0.01264	0.816	0.00016	0.021558
ENSG00000107738	*VSIR*	-0.938	0.00026	0.03641	-0.958	0.00016	0.021558
ENSG00000139344	*AMDHD1*	-0.907	0.00236	0.15843	-1.080	0.00016	0.021558
ENSG00000069535	*MAOB*	-1.916	0.00010	0.01970	-1.684	0.00016	0.021558
ENSG00000162849	*KIF26B*	-1.777	0.00128	0.10751	-1.721	0.00016	0.021558
ENSG00000116005	*PCYOX1*	-0.785	0.00019	0.02853	-0.790	0.00017	0.022494
ENSG00000062038	*CDH3*	-0.779	0.00024	0.03434	-0.796	0.00017	0.022494
ENSG00000241322	*CDRT1*	1.586	0.00054	0.06333	1.447	0.00017	0.022494
ENSG00000225614	*ZNF469*	-0.811	0.00018	0.02762	-0.812	0.00017	0.022814
ENSG00000144959	*NCEH1*	-0.826	0.00017	0.02720	-0.824	0.00017	0.022814
ENSG00000179163	*FUCA1*	-0.876	0.00023	0.03252	-0.881	0.00017	0.022866
ENSG00000139117	*CPNE8*	-0.828	0.00030	0.04005	-0.832	0.00018	0.023303
ENSG00000001036	*FUCA2*	-0.782	0.00022	0.03153	-0.788	0.00019	0.024566
ENSG00000143520	*FLG2*	6.734	0.00035	0.04482	6.838	0.00019	0.024593
ENSG00000181104	*F2R*	-0.826	0.00031	0.04050	-0.851	0.00019	0.024711

ENSG00000125534	*PPDPF*	-0.751	0.00039	0.04893	-0.787	0.00019	0.025138
ENSG00000108924	*HLF*	-0.901	9.49E-05	0.01876	-0.857	0.00019	0.025306
ENSG00000168646	*AXIN2*	-0.810	0.00035	0.04482	-0.843	0.00020	0.025538
ENSG00000051128	*HOMER3*	-0.875	0.00017	0.02638	-0.857	0.00020	0.025712
ENSG00000133121	*STARD13*	-0.966	0.00025	0.03529	-0.975	0.00020	0.025712
ENSG00000112276	*BVES*	-1.157	0.00026	0.03636	-1.149	0.00020	0.025712
ENSG00000198786	*ND5*	0.769	0.00018	0.02807	0.764	0.00020	0.025846
ENSG00000101911	*PRPS2*	-0.786	0.00020	0.02988	-0.784	0.00020	0.025874
ENSG00000186063	*AIDA*	-0.773	0.00039	0.04893	-0.788	0.00020	0.025986
ENSG00000074181	*NOTCH3*	-0.943	0.00026	0.03641	-0.935	0.00020	0.026033
ENSG00000010626	*LRRC23*	0.971	0.00012	0.02125	0.917	0.00021	0.026413
ENSG00000166313	*APBB1*	-0.843	0.00068	0.07294	-0.893	0.00021	0.026628
ENSG00000134569	*LRP4*	-0.870	0.00024	0.03421	-0.864	0.00021	0.026628
ENSG00000188643	*S100A16*	-0.795	0.00025	0.03536	-0.800	0.00021	0.026628
ENSG00000048540	*LMO3*	1.177	0.00015	0.02497	1.162	0.00021	0.026823
ENSG00000234155	*LINC02535*	-1.983	9.87E-05	0.01921	-1.850	0.00021	0.026834
ENSG00000008735	*MAPK8IP2*	-0.788	0.00055	0.06408	-0.836	0.00022	0.027067
ENSG00000092068	*SLC7A8*	-0.991	0.00018	0.02779	-0.952	0.00022	0.027067
ENSG00000169131	*ZNF354A*	-0.860	0.00024	0.03402	-0.856	0.00022	0.027321
ENSG00000114841	*DNAH1*	0.840	0.00028	0.03818	0.838	0.00022	0.027372
ENSG00000112561	*TFEB*	-1.099	0.00036	0.04529	-1.096	0.00022	0.027967
ENSG00000076716	*GPC4*	-0.874	9.60E-05	0.01889	-0.818	0.00023	0.028206
ENSG00000155660	*PDIA4*	-0.773	0.00019	0.02853	-0.761	0.00023	0.028428
ENSG00000128872	*TMOD2*	-0.856	0.00019	0.02895	-0.838	0.00023	0.028459
ENSG00000249476	*LOC285638*	1.162	0.00012	0.02143	1.101	0.00023	0.028790
ENSG00000197763	*TXNRD3*	-0.908	0.00029	0.03945	-0.892	0.00024	0.029049
ENSG00000070540	*WIPI1*	-0.938	0.00063	0.06939	-0.969	0.00024	0.029119
ENSG00000262156	*APOBEC3A*	1.491	0.00134	0.11060	1.267	0.00024	0.029229
ENSG00000007402	*CACNA2D2*	-0.903	0.00049	0.05886	-0.921	0.00024	0.029426
ENSG00000185686	*PRAME*	-0.695	0.00155	0.12143	-0.777	0.00025	0.030147
ENSG00000162738	*VANGL2*	1.228	0.00017	0.02668	1.172	0.00025	0.030167
ENSG00000113108	*APBB3*	0.869	0.00021	0.03054	0.851	0.00025	0.030451
ENSG00000137944	*KYAT3*	-0.836	0.00039	0.04859	-0.844	0.00026	0.030888
ENSG00000068394	*GPKOW*	0.848	0.00018	0.02799	0.811	0.00027	0.032157
ENSG00000130829	*DUSP9*	-0.820	0.00019	0.02878	-0.798	0.00027	0.032157
ENSG00000138685	*FGF2*	-0.826	0.00028	0.03830	-0.828	0.00027	0.032885
ENSG00000162378	*ZYG11B*	-0.764	0.00032	0.04170	-0.770	0.00028	0.033026
ENSG00000092871	*RFFL*	0.744	0.00063	0.06939	0.789	0.00028	0.033141
ENSG00000143036	*SLC44A3*	-0.830	0.00077	0.07986	-0.869	0.00028	0.033141
ENSG00000072062	*PRKACA*	0.774	0.00028	0.03818	0.771	0.00028	0.033141
ENSG00000079308	*TNS1*	-0.867	0.00023	0.03313	-0.847	0.00028	0.033141
ENSG00000175745	*NR2F1*	-0.878	0.00029	0.03942	-0.882	0.00028	0.033488
ENSG00000184903	*IMMP2L*	0.803	0.00061	0.06738	0.842	0.00028	0.033552
ENSG00000240288	*GHRLOS*	1.097	0.00017	0.02740	1.054	0.00028	0.033552
ENSG00000126709	*IFI6*	-1.062	0.00126	0.10751	-1.141	0.00028	0.033552
ENSG00000121073	*SLC35B1*	-0.733	0.00060	0.06643	-0.770	0.00029	0.033634
ENSG00000064652	*SNX24*	-0.947	0.00013	0.02247	-0.880	0.00029	0.033727
ENSG00000106066	*CPVL*	-0.836	0.00021	0.03085	-0.802	0.00029	0.034269
ENSG00000092096	*SLC22A17*	-0.911	0.00097	0.09241	-0.978	0.00029	0.034269
ENSG00000145692	*BHMT*	1.777	0.00016	0.02568	1.643	0.00029	0.034269
ENSG00000143845	*ETNK2*	-0.830	0.00082	0.08255	-0.882	0.00030	0.034515
ENSG00000075213	*SEMA3A*	-1.627	0.00255	0.16658	-1.713	0.00031	0.035151
ENSG00000170175	*CHRNB1*	-1.114	0.00017	0.02734	-0.900	0.00031	0.035157
ENSG00000249242	*TMEM150C*	-1.245	0.00058	0.06560	-1.300	0.00031	0.035157
ENSG00000132964	*CDK8*	-0.780	0.00035	0.04482	-0.780	0.00031	0.035317
ENSG00000151150	*ANK3*	0.945	0.00018	0.02807	0.877	0.00031	0.035317

ENSG00000181481	*RNF135*	-0.919	0.00030	0.03956	-0.906	0.00031	0.035486
ENSG00000150676	*CCDC83*	2.756	0.00010	0.01970	2.440	0.00031	0.035522
ENSG00000101210	*EEF1A2*	-0.741	0.00034	0.04411	-0.745	0.00031	0.035522
ENSG00000170385	*SLC30A1*	-0.751	0.00038	0.04812	-0.760	0.00032	0.036346
ENSG00000002586	*CD99*	-0.678	0.01110	0.38627	-0.819	0.00032	0.036464
ENSG00000133739	*LRRCC1*	-0.775	0.00036	0.04542	-0.774	0.00032	0.036482
ENSG00000116667	*C1orf21*	-0.830	0.00270	0.17191	-0.963	0.00032	0.036482
ENSG00000129993	*CBFA2T3*	-0.845	0.00034	0.04411	-0.838	0.00033	0.036763
ENSG00000145349	*CAMK2D*	-0.831	0.00021	0.03040	-0.793	0.00033	0.037059
ENSG00000100644	*HIF1A*	-0.711	0.00065	0.07123	-0.748	0.00034	0.037350
ENSG00000107263	*RAPGEF1*	-0.745	0.00042	0.05210	-0.754	0.00034	0.037350
ENSG00000169116	*PARM1*	-0.837	0.00048	0.05814	-0.860	0.00034	0.037539
ENSG00000171388	*APLN*	-2.785	0.00034	0.04403	-2.788	0.00034	0.037698
ENSG00000158683	*PKD1L1*	0.769	0.00180	0.13422	0.865	0.00034	0.037870
ENSG00000244165	*P2RY11*	0.872	0.00030	0.03945	0.857	0.00034	0.037981
ENSG00000018236	*CNTN1*	-0.988	0.00257	0.16734	-1.109	0.00035	0.038303
ENSG00000005059	*MCUB*	-0.739	0.00121	0.10581	-0.805	0.00035	0.038615
ENSG00000170396	*ZNF804A*	-6.456	0.00127	0.10751	-6.696	0.00035	0.038615
ENSG00000197142	*ACSL5*	-0.844	0.00194	0.13898	-0.957	0.00035	0.038720
ENSG00000198910	*L1CAM*	-0.768	0.00076	0.07937	-0.798	0.00036	0.038796
ENSG00000033867	*SLC4A7*	-0.716	0.00070	0.07449	-0.751	0.00036	0.039186
ENSG00000127528	*KLF2*	-0.803	0.00045	0.05455	-0.809	0.00036	0.039225
ENSG00000064300	*NGFR*	-0.921	0.00019	0.02853	-0.872	0.00036	0.039225
ENSG00000185015	*CA13*	-0.779	0.00046	0.05566	-0.788	0.00037	0.039743
ENSG00000172936	*MYD88*	-0.795	0.00043	0.05308	-0.802	0.00037	0.039743
ENSG00000167191	*GPRC5B*	-0.850	0.00043	0.05271	-0.855	0.00037	0.040095
ENSG00000180539	*C9orf139*	1.768	0.00012	0.02110	1.560	0.00038	0.040537
ENSG00000109063	*MYH3*	0.802	0.00090	0.08750	0.839	0.00038	0.040689
ENSG00000196505	*GDAP2*	-0.768	0.00033	0.04285	-0.757	0.00038	0.041019
ENSG00000108511	*HOXB6*	0.860	0.00063	0.06928	0.887	0.00038	0.041019
ENSG00000162434	*JAK1*	-0.746	0.00037	0.04712	-0.740	0.00040	0.042240
ENSG00000100605	*ITPK1*	-0.889	0.00016	0.02626	-0.774	0.00040	0.042437
ENSG00000116711	*PLA2G4A*	-0.938	0.00027	0.03713	-0.875	0.00041	0.042671
ENSG00000105246	*EBI3*	-3.973	0.00014	0.02408	-3.135	0.00041	0.043375
ENSG00000176490	*DIRAS1*	-0.785	0.00043	0.05271	-0.787	0.00042	0.043422
ENSG00000110422	*HIPK3*	-0.752	0.00035	0.04453	-0.740	0.00042	0.043422
ENSG00000131480	*AOC2*	0.756	0.00090	0.08750	0.799	0.00042	0.043422
ENSG00000130066	*SAT1*	0.780	0.00019	0.02853	0.735	0.00042	0.043422
ENSG00000148680	*HTR7*	0.895	0.00015	0.02525	0.819	0.00043	0.044201
ENSG00000146386	*ABRACL*	-0.817	0.00040	0.04931	-0.801	0.00043	0.044201
ENSG00000154127	*UBASH3B*	-0.772	0.00066	0.07169	-0.792	0.00044	0.044984
ENSG00000175395	*ZNF25*	-0.998	0.00025	0.03562	-0.949	0.00044	0.045489
ENSG00000106013	*ANKRD7*	6.734	0.00035	0.04482	3.875	0.00045	0.045958
ENSG00000110042	*DTX4*	-3.804	0.00045	0.05449	-3.807	0.00045	0.045958
ENSG00000236024	*PRRX2-AS1*	-3.804	0.00045	0.05449	-3.807	0.00045	0.045958
ENSG00000167508	*MVD*	0.771	0.00024	0.03421	0.734	0.00045	0.045985
ENSG00000140280	*LYSMD2*	-0.888	0.00055	0.06382	-0.899	0.00045	0.046025
ENSG00000163817	*SLC6A20*	1.178	0.00250	0.16464	1.307	0.00046	0.046940
ENSG00000188910	*GJB3*	0.781	0.00050	0.05977	0.786	0.00046	0.046997
ENSG00000131730	*CKMT2*	0.928	0.00039	0.04920	0.913	0.00046	0.047066
ENSG00000058668	*ATP2B4*	0.742	0.00057	0.06479	0.749	0.00047	0.047204
ENSG00000171552	*BCL2L1*	-0.735	0.00054	0.06277	-0.743	0.00047	0.047334
ENSG00000185947	*ZNF267*	-0.744	0.00056	0.06475	-0.754	0.00047	0.047685
ENSG00000104213	*PDGFRL*	-1.253	6.21E-05	0.01366	-1.047	0.00048	0.048325
ENSG00000150594	*ADRA2A*	2.332	0.00076	0.07937	2.385	0.00049	0.049065
ENSG00000115112	*TFCP2L1*	-0.753	0.00051	0.06028	-0.751	0.00049	0.049530

ENSG00000138639	*ARHGAP24*	-1.343	0.00028	0.03818	-1.227	0.00050	0.049624
ENSG00000128228	*SDF2L1*	-0.752	0.00064	0.06974	-0.761	0.00050	0.050033
ENSG00000160588	*MPZL3*	0.821	0.00045	0.05462	0.809	0.00050	0.050077
ENSG00000074370	*ATP2A3*	-0.867	0.00042	0.05240	-0.839	0.00051	0.050302
ENSG00000140682	*TGFB1I1*	-1.361	0.00140	0.11358	-1.374	0.00051	0.050775
ENSG00000157502	*PWWP3B*	-0.732	0.00059	0.06633	-0.738	0.00052	0.051154
ENSG00000139684	*ESD*	-0.728	0.00054	0.06333	-0.724	0.00052	0.051577
ENSG00000159176	*CSRP1*	-0.719	0.00068	0.07300	-0.732	0.00053	0.052113
ENSG00000111275	*ALDH2*	-0.840	0.00050	0.05928	-0.811	0.00055	0.054105
ENSG00000170608	*FOXA3*	-0.861	0.00135	0.11096	-0.919	0.00056	0.054909
ENSG00000163154	*TNFAIP8L2*	1.325	0.00072	0.07570	1.343	0.00056	0.055008
ENSG00000114270	*COL7A1*	1.018	3.12E-05	0.00794	0.790	0.00056	0.055051
ENSG00000109586	*GALNT7*	-0.736	0.00068	0.07282	-0.741	0.00057	0.055376
ENSG00000163545	*NUAK2*	0.879	0.00079	0.08089	0.891	0.00057	0.055556
ENSG00000145248	*SLC10A4*	-0.816	0.00084	0.08361	-0.834	0.00059	0.057299
ENSG00000187498	*COL4A1*	2.075	0.00189	0.13770	2.019	0.00060	0.057444
ENSG00000141756	*FKBP10*	-0.698	0.00090	0.08753	-0.721	0.00060	0.057770
ENSG00000165471	*MBL2*	1.729	0.00084	0.08426	1.762	0.00060	0.057860
ENSG00000142235	*LMTK3*	0.759	0.00103	0.09657	0.788	0.00060	0.057860
ENSG00000170542	*SERPINB9*	-0.729	0.00073	0.07704	-0.736	0.00062	0.058748
ENSG00000196776	*CD47*	-0.703	0.00109	0.09927	-0.732	0.00062	0.058748
ENSG00000138613	*APH1B*	-0.960	0.00095	0.09081	-0.972	0.00062	0.058748
ENSG00000189067	*LITAF*	-0.741	0.00069	0.07302	-0.746	0.00062	0.058748
ENSG00000038427	*VCAN*	-0.692	0.00077	0.07986	-0.704	0.00062	0.058835
ENSG00000144118	*RALB*	-0.761	0.00051	0.06028	-0.747	0.00062	0.058835
ENSG00000198467	*TPM2*	0.686	0.00100	0.09405	0.710	0.00062	0.058958
ENSG00000109861	*CTSC*	-0.703	0.00080	0.08142	-0.716	0.00062	0.058981
ENSG00000253741	*LNCOC1*	1.143	0.00051	0.06028	1.106	0.00063	0.059084
ENSG00000081087	*OSTM1*	-0.777	0.00058	0.06604	-0.765	0.00063	0.059084
ENSG00000100906	*NFKBIA*	0.724	0.00068	0.07294	0.725	0.00064	0.059696
ENSG00000172461	*FUT9*	1.150	0.00037	0.04666	1.089	0.00064	0.059696
ENSG00000101144	*BMP7*	-1.128	0.00284	0.17715	-1.217	0.00064	0.059696
ENSG00000087589	*CASS4*	0.980	0.00513	0.25083	1.178	0.00064	0.059696
ENSG00000165757	*JCAD*	-0.781	0.00086	0.08504	-0.800	0.00064	0.059749
ENSG00000134352	*IL6ST*	-0.736	0.00055	0.06382	-0.722	0.00065	0.060018
ENSG00000198556	*ZNF789*	0.756	0.00064	0.07063	0.751	0.00066	0.060957
ENSG00000126351	*THRA*	-0.750	0.00109	0.09927	-0.773	0.00066	0.060957
ENSG00000112183	*RBM24*	-1.315	0.00116	0.10313	-1.352	0.00066	0.060957
ENSG00000068078	*FGFR3*	-0.743	0.00084	0.08361	-0.750	0.00066	0.061245
ENSG00000152689	*RASGRP3*	-0.905	0.00473	0.23767	-1.042	0.00067	0.061447
ENSG00000167767	*KRT80*	0.740	0.00051	0.06042	0.722	0.00067	0.061638
ENSG00000069849	*ATP1B3*	-0.697	0.00088	0.08625	-0.710	0.00067	0.061638
ENSG00000123612	*ACVR1C*	-0.910	0.00190	0.13772	-0.981	0.00067	0.061638
ENSG00000242485	*MRPL20*	0.709	0.00098	0.09257	0.724	0.00068	0.062032
ENSG00000160401	*CFAP157*	0.941	0.00066	0.07169	0.933	0.00068	0.062032
ENSG00000252010	*SCARNA5*	-0.740	0.00130	0.10868	-0.742	0.00069	0.062442
ENSG00000198624	*CCDC69*	-0.933	0.00057	0.06479	-0.896	0.00069	0.062894
ENSG00000128594	*LRRC4*	0.880	0.00068	0.07294	0.873	0.00070	0.062979
ENSG00000131069	*ACSS2*	0.771	0.00030	0.04005	0.718	0.00071	0.063887
ENSG00000127951	*FGL2*	1.253	0.00042	0.05187	1.185	0.00071	0.064166
ENSG00000165929	*TC2N*	-0.771	0.00489	0.24380	-0.818	0.00073	0.065480
ENSG00000253276	*CCDC71L*	-0.739	0.00080	0.08119	-0.742	0.00074	0.066275
ENSG00000138698	*RAP1GDS1*	-0.719	0.00059	0.06643	-0.704	0.00074	0.066473
ENSG00000206258	*TNXB*	1.207	0.00039	0.04847	0.772	0.00075	0.066897
ENSG00000005513	*SOX8*	-0.922	0.00049	0.05894	-0.886	0.00076	0.067295
ENSG00000153904	*DDAH1*	-0.691	0.00102	0.09555	-0.708	0.00076	0.067530

ENSG00000159403	*C1R*	-0.974	0.00372	0.20877	-1.067	0.00076	0.067530
ENSG00000134285	*FKBP11*	0.756	0.00077	0.07986	0.755	0.00076	0.067530
ENSG00000174529	*TMEM81*	0.960	0.00100	0.09405	0.977	0.00077	0.067676
ENSG00000168528	*SERINC2*	-0.785	0.00093	0.08983	-0.786	0.00077	0.067705
ENSG00000162998	*FRZB*	1.104	0.00111	0.10055	1.112	0.00078	0.068096
ENSG00000169019	*COMMD8*	-0.791	0.00043	0.05254	-0.747	0.00078	0.068096
ENSG00000143869	*GDF7*	1.164	0.00078	0.08054	1.161	0.00078	0.068383
ENSG00000134007	*ADAM20*	1.358	0.00031	0.04005	1.194	0.00080	0.069498
ENSG00000179954	*SSC5D*	1.037	0.00428	0.22603	1.194	0.00080	0.069498
ENSG00000153933	*DGKE*	-0.802	0.00139	0.11318	-0.828	0.00081	0.069957
ENSG00000138347	*MYPN*	1.249	0.00212	0.14791	1.283	0.00081	0.069992
ENSG00000144306	*SCRN3*	-0.815	0.00058	0.06598	-0.781	0.00081	0.070042
ENSG00000139998	*RAB15*	-0.758	0.00110	0.09972	-0.774	0.00082	0.070597
ENSG00000049768	*FOXP3*	2.131	0.00125	0.10738	2.182	0.00082	0.070662
ENSG00000152229	*PSTPIP2*	-0.697	0.00134	0.11090	-0.717	0.00083	0.071599
ENSG00000177000	*MTHFR*	0.773	0.00080	0.08119	0.765	0.00084	0.072054
ENSG00000259158	*ADAM20P1*	1.314	0.00222	0.15314	1.353	0.00085	0.072549
ENSG00000147852	*VLDLR*	1.273	0.00108	0.09927	1.291	0.00086	0.073212
ENSG00000049860	*HEXB*	-0.673	0.00151	0.11901	-0.700	0.00086	0.073328
ENSG00000101977	*MCF2*	1.855	0.00128	0.10751	1.774	0.00086	0.073746
ENSG00000143127	*ITGA10*	1.237	0.00120	0.10499	1.167	0.00088	0.074677
ENSG00000164509	*IL31RA*	2.022	0.00060	0.06643	1.896	0.00088	0.074944
ENSG00000121743	*GJA3*	-0.793	0.00067	0.07273	-0.773	0.00090	0.075881
ENSG00000077274	*CAPN6*	-0.893	0.00133	0.11052	-0.903	0.00091	0.076506
ENSG00000160752	*FDPS*	0.746	0.00045	0.05455	0.696	0.00091	0.076506
ENSG00000128016	*ZFP36*	0.737	0.00077	0.07998	0.725	0.00092	0.076582
ENSG00000242265	*PEG10*	-0.854	0.00125	0.10751	-0.877	0.00092	0.076582
ENSG00000075826	*SEC31B*	0.702	0.00136	0.11158	0.719	0.00092	0.076582
ENSG00000112893	*MAN2A1*	-0.690	0.00099	0.09405	-0.693	0.00092	0.076956
ENSG00000167004	*PDIA3*	-0.651	0.00168	0.12859	-0.683	0.00093	0.077028
ENSG00000127946	*HIP1*	-0.680	0.00157	0.12265	-0.706	0.00093	0.077028
ENSG00000173402	*DAG1*	-0.678	0.00109	0.09927	-0.687	0.00093	0.077044
ENSG00000103067	*ESRP2*	-0.696	0.00112	0.10078	-0.703	0.00093	0.077123
ENSG00000120693	*SMAD9*	-0.789	0.00117	0.10366	-0.799	0.00093	0.077123
ENSG00000269896	*LOC100129534*	0.919	0.00088	0.08625	0.910	0.00094	0.077123
ENSG00000101000	*PROCR*	-0.772	0.00136	0.11158	-0.792	0.00094	0.077123
ENSG00000087085	*ACHE*	-1.057	0.00073	0.07690	-1.009	0.00094	0.077123
ENSG00000185551	*NR2F2*	-0.695	0.00095	0.09081	-0.695	0.00094	0.077123
ENSG00000151690	*MFSD6*	-0.748	0.00159	0.12308	-0.783	0.00094	0.077123
ENSG00000107954	*NEURL1*	-1.077	0.00106	0.09779	-1.056	0.00095	0.077123
ENSG00000274194	*MBOAT7*	0.869	0.00238	0.15961	0.706	0.00095	0.077123
ENSG00000112394	*SLC16A10*	-0.785	0.00076	0.07974	-0.769	0.00095	0.077246
ENSG00000049323	*LTBP1*	-0.729	0.00126	0.10751	-0.733	0.00097	0.078654
ENSG00000119326	*CTNNAL1*	-0.674	0.00120	0.10514	-0.684	0.00097	0.078654
ENSG00000083444	*PLOD1*	-0.633	0.00226	0.15451	-0.682	0.00098	0.079170
ENSG00000255150	*EID3*	0.766	0.00083	0.08322	0.756	0.00098	0.079350
ENSG00000106537	*TSPAN13*	-0.764	0.00094	0.09065	-0.753	0.00098	0.079350
ENSG00000108846	*ABCC3*	0.711	0.00078	0.08054	0.696	0.00098	0.079350
ENSG00000204304	*PBX2*	0.857	0.00094	0.09008	0.702	0.00098	0.079350
ENSG00000100934	*SEC23A*	0.657	0.00170	0.12902	0.687	0.00099	0.079350
ENSG00000240280	*TCAM1P*	-0.904	0.00088	0.08643	-0.856	0.00099	0.079350
ENSG00000167861	*HID1*	-0.800	0.00110	0.09972	-0.782	0.00100	0.079888
ENSG00000122591	*FAM126A*	-0.698	0.00108	0.09927	-0.701	0.00100	0.079888
ENSG00000185864	*NPIPB4*	0.685	0.00182	0.13469	0.685	0.00100	0.080011
ENSG00000164323	*CFAP97*	-0.684	0.00100	0.09432	-0.683	0.00102	0.081306
ENSG00000116141	*MARK1*	-0.756	0.00069	0.07302	-0.721	0.00102	0.081382

ENSG00000124493	*GRM4*	-0.860	0.00339	0.19758	-0.944	0.00102	0.081382
ENSG00000109501	*WFS1*	-0.674	0.00228	0.15497	-0.719	0.00102	0.081556
ENSG00000123901	*GPR83*	1.331	0.00018	0.02788	1.121	0.00104	0.082157
ENSG00000101440	*ASIP*	1.124	0.00104	0.09658	1.121	0.00104	0.082157
ENSG00000174871	*CNIH2*	-1.085	0.00224	0.15371	-1.156	0.00104	0.082157
ENSG00000262999	*LOC101927131*	1.119	0.00087	0.08569	1.080	0.00105	0.083448
ENSG00000157064	*NMNAT2*	1.053	0.00151	0.11889	1.080	0.00105	0.083448
ENSG00000166130	*IKBIP*	-0.760	0.00108	0.09927	-0.758	0.00106	0.083680
ENSG00000213906	*LTB4R2*	0.950	0.00114	0.10193	0.829	0.00107	0.084220
ENSG00000118785	*SPP1*	1.126	0.00835	0.33438	1.269	0.00108	0.084500
ENSG00000143870	*PDIA6*	-0.641	0.00227	0.15456	-0.682	0.00108	0.084500
ENSG00000138463	*SLC49A4*	-0.760	0.00095	0.09065	-0.743	0.00109	0.084513
ENSG00000163755	*HPS3*	-0.735	0.00134	0.11060	-0.736	0.00109	0.084513
ENSG00000141448	*GATA6*	-0.822	0.00139	0.11318	-0.824	0.00109	0.084602
ENSG00000135916	*ITM2C*	-0.728	0.00065	0.07123	-0.694	0.00109	0.084772
ENSG00000064115	*TM7SF3*	-0.737	0.00062	0.06901	-0.697	0.00110	0.085408
ENSG00000169246	*NPIPB3*	0.596	0.00820	0.32983	0.681	0.00111	0.085713
ENSG00000136997	*MYC*	-0.668	0.00120	0.10514	-0.672	0.00111	0.085725
ENSG00000180525	*PRR26*	0.797	0.00212	0.14791	0.841	0.00111	0.085725
ENSG00000146233	*CYP39A1*	-1.008	0.00327	0.19347	-1.082	0.00112	0.086254
ENSG00000146453	*PNLDC1*	1.211	0.00232	0.15652	1.228	0.00112	0.086254
ENSG00000143416	*SELENBP1*	-1.637	0.00058	0.06552	-1.411	0.00113	0.086644
ENSG00000160161	*CILP2*	-0.728	0.00103	0.09657	-0.719	0.00114	0.087071
ENSG00000149591	*TAGLN*	0.886	0.00156	0.12233	0.891	0.00114	0.087103
ENSG00000147883	*CDKN2B*	0.702	0.00086	0.08504	0.685	0.00115	0.087525
ENSG00000170965	*PLAC1*	-1.265	0.00192	0.13820	-1.250	0.00115	0.087604
ENSG00000101928	*MOSPD1*	-0.678	0.00140	0.11371	-0.688	0.00115	0.087624
ENSG00000070081	*NUCB2*	-0.719	0.00110	0.09972	-0.706	0.00116	0.087847
ENSG00000135709	*KIAA0513*	-0.706	0.00134	0.11060	-0.710	0.00116	0.087996
ENSG00000133138	*TBC1D8B*	-0.703	0.00128	0.10751	-0.704	0.00118	0.088904
ENSG00000106328	*FSCN3*	2.962	0.00201	0.14239	3.045	0.00119	0.088904
ENSG00000159200	*RCAN1*	-0.737	0.00194	0.13898	-0.763	0.00119	0.088904
ENSG00000231889	*TRAF3IP2-AS1*	0.902	0.00087	0.08569	0.882	0.00119	0.088904
ENSG00000156515	*HK1*	-0.692	0.00144	0.11559	-0.692	0.00120	0.088904
ENSG00000004660	*CAMKK1*	0.717	0.00318	0.19072	0.770	0.00120	0.088904
ENSG00000148700	*ADD3*	-0.696	0.00127	0.10751	-0.694	0.00120	0.089077
ENSG00000079819	*EPB41L2*	-0.675	0.00119	0.10447	-0.672	0.00122	0.090214
ENSG00000085741	*WNT11*	-1.535	0.00339	0.19758	-1.663	0.00122	0.090214
ENSG00000186642	*PDE2A*	-1.485	0.00718	0.30770	-1.663	0.00122	0.090214
ENSG00000140511	*HAPLN3*	-1.001	0.00192	0.13820	-1.013	0.00123	0.090409
ENSG00000141542	*RAB40B*	-0.842	0.00128	0.10751	-0.820	0.00123	0.090752
ENSG00000185513	*L3MBTL1*	0.724	0.00083	0.08348	0.697	0.00124	0.091023
ENSG00000105605	*CACNG7*	-2.255	0.00076	0.07937	-2.057	0.00125	0.091141
ENSG00000146038	*DCDC2*	2.017	0.00285	0.17715	2.128	0.00125	0.091141
ENSG00000153060	*TEKT5*	2.082	0.00433	0.22662	2.128	0.00125	0.091141
ENSG00000178695	*KCTD12*	-1.029	0.00147	0.11806	-1.046	0.00125	0.091189
ENSG00000078081	*LAMP3*	-1.077	0.00338	0.19758	-1.140	0.00126	0.091500
ENSG00000164251	*F2RL1*	-0.684	0.00125	0.10738	-0.682	0.00126	0.091777
ENSG00000255568	*BRWD1-AS2*	-6.321	0.00242	0.16037	-6.458	0.00127	0.091777
ENSG00000137558	*PI15*	1.855	0.00128	0.10751	1.852	0.00128	0.092469
ENSG00000164023	*SGMS2*	-0.672	0.00167	0.12797	-0.687	0.00129	0.092762
ENSG00000285533	*RELA-DT*	1.968	0.00191	0.13772	1.926	0.00129	0.092776
ENSG00000213424	*KRT222*	-1.083	0.00395	0.21680	-1.170	0.00131	0.094003
ENSG00000204128	*C2orf72*	-1.407	0.00113	0.10169	-1.348	0.00131	0.094040
ENSG00000147650	*LRP12*	-0.712	0.00196	0.14007	-0.732	0.00131	0.094040
ENSG00000144827	*ABHD10*	-0.676	0.00116	0.10324	-0.667	0.00132	0.094222

ENSG00000148803	*FUOM*	-0.910	0.00089	0.08704	-0.844	0.00132	0.094222
ENSG00000178764	*ZHX2*	-0.756	0.00139	0.11336	-0.759	0.00133	0.094367
ENSG00000175899	*A2M*	0.931	0.00140	0.11363	0.929	0.00133	0.094774
ENSG00000144935	*TRPC1*	-0.870	0.00171	0.12918	-0.884	0.00134	0.094774
ENSG00000008300	*CELSR3*	0.685	0.00143	0.11495	0.686	0.00134	0.094774
ENSG00000120215	*MLANA*	0.880	0.00230	0.15571	0.917	0.00135	0.095039
ENSG00000243970	*PPIEL*	0.722	0.00151	0.11901	0.724	0.00135	0.095049
ENSG00000115129	*TP53I3*	-0.773	0.00167	0.12797	-0.772	0.00135	0.095238
ENSG00000064666	*CNN2*	0.695	0.00086	0.08504	0.665	0.00136	0.095683
ENSG00000143320	*CRABP2*	-0.692	0.00088	0.08643	-0.665	0.00137	0.095976
ENSG00000112715	*VEGFA*	0.657	0.00182	0.13469	0.673	0.00137	0.096145
ENSG00000015475	*BID*	-0.702	0.00114	0.10220	-0.688	0.00137	0.096177
ENSG00000111328	*CDK2AP1*	-0.636	0.00284	0.17715	-0.676	0.00138	0.096378
ENSG00000161682	*FAM171A2*	-0.798	0.00092	0.08907	-0.764	0.00139	0.096712
ENSG00000197043	*ANXA6*	-0.677	0.00150	0.11877	-0.672	0.00139	0.096973
ENSG00000276068	*NAIP*	0.707	0.00475	0.23866	0.678	0.00140	0.097171
ENSG00000197329	*PELI1*	-0.690	0.00451	0.23192	-0.765	0.00141	0.097649
ENSG00000128039	*SRD5A3*	-0.679	0.00298	0.18330	-0.726	0.00141	0.097808
ENSG00000101347	*SAMHD1*	-0.661	0.00191	0.13816	-0.677	0.00143	0.098618
ENSG00000128590	*DNAJB9*	-0.710	0.00165	0.12688	-0.713	0.00143	0.098706
ENSG00000188677	*PARVB*	-0.751	0.00106	0.09830	-0.713	0.00144	0.099360
ENSG00000162627	*SNX7*	-0.679	0.00219	0.15144	-0.696	0.00145	0.099423
ENSG00000140948	*ZCCHC14*	-0.650	0.00190	0.13772	-0.666	0.00145	0.099457
ENSG00000260852	*FBXL19-AS1*	0.761	0.00118	0.10396	0.749	0.00146	0.100242
ENSG00000172086	*KRCC1*	-0.737	0.00223	0.15343	-0.760	0.00146	0.100242
ENSG00000243716	*NPIPB5*	0.605	0.00453	0.23236	0.660	0.00147	0.100317
ENSG00000137509	*PRCP*	-0.648	0.00269	0.17191	-0.682	0.00148	0.100806
ENSG00000151553	*FAM160B1*	-0.676	0.00158	0.12297	-0.677	0.00148	0.100942
ENSG00000164237	*CMBL*	-0.704	0.00105	0.09737	-0.678	0.00148	0.100942
ENSG00000183691	*NOG*	-2.575	0.00149	0.11820	-2.578	0.00149	0.101054
ENSG00000221963	*APOL6*	1.614	0.00229	0.15553	1.578	0.00149	0.101054
ENSG00000198598	*MMP17*	-0.799	0.00433	0.22662	-0.865	0.00149	0.101054
ENSG00000167384	*ZNF180*	-0.707	0.00166	0.12758	-0.711	0.00150	0.101332
ENSG00000129538	*RNASE1*	0.878	0.00101	0.09528	0.846	0.00152	0.102151
ENSG00000197580	*BCO2*	0.840	0.00123	0.10669	0.809	0.00152	0.102224
ENSG00000196083	*IL1RAP*	-0.785	0.00190	0.13772	-0.790	0.00153	0.102704
ENSG00000114646	*CSPG5*	-0.741	0.00342	0.19844	-0.790	0.00153	0.102704
ENSG00000184432	*COPB2*	-0.631	0.00236	0.15843	-0.654	0.00156	0.104319
ENSG00000164466	*SFXN1*	-0.647	0.00184	0.13559	-0.656	0.00157	0.104765
ENSG00000076641	*PAG1*	-0.754	0.00222	0.15327	-0.773	0.00158	0.105797
ENSG00000182287	*AP1S2*	-0.702	0.00158	0.12306	-0.693	0.00159	0.105838
ENSG00000102057	*KCND1*	0.752	0.00117	0.10366	0.729	0.00159	0.105838
ENSG00000255284	*LOC171391*	0.891	0.00269	0.17191	0.918	0.00159	0.105838
ENSG00000148154	*UGCG*	-0.679	0.00132	0.11004	-0.666	0.00159	0.105838
ENSG00000176658	*MYO1D*	-0.683	0.00169	0.12871	-0.682	0.00161	0.106596
ENSG00000163516	*ANKZF1*	0.715	0.00101	0.09528	0.680	0.00161	0.106667
ENSG00000142552	*RCN3*	-1.744	7.10E-06	0.00250	-1.099	0.00162	0.107173
ENSG00000119138	*KLF9*	-0.882	0.00171	0.12947	-0.886	0.00164	0.107871
ENSG00000171811	*CFAP46*	0.981	0.00114	0.10193	0.900	0.00164	0.107871
ENSG00000198064	*NPIPB13*	0.671	0.00273	0.17341	0.658	0.00164	0.107871
ENSG00000104783	*KCNN4*	-0.652	0.00309	0.18676	-0.690	0.00165	0.108585
ENSG00000118855	*MFSD1*	-0.647	0.00456	0.23309	-0.699	0.00166	0.108903
ENSG00000164161	*HHIP*	1.224	0.00270	0.17191	1.271	0.00167	0.109182
ENSG00000240370	*RPL13P5*	0.902	0.00146	0.11690	0.885	0.00167	0.109182
ENSG00000086730	*LAT2*	0.763	0.00172	0.12947	0.749	0.00167	0.109182
ENSG00000147166	*ITGB1BP2*	0.823	0.00182	0.13495	0.820	0.00167	0.109182

ENSG00000184489	*PTP4A3*	-1.001	0.00871	0.34279	-0.923	0.00167	0.109182
ENSG00000163527	*STT3B*	-0.625	0.00248	0.16329	-0.648	0.00168	0.109399
ENSG00000141179	*PCTP*	-0.676	0.00465	0.23504	-0.739	0.00168	0.109533
ENSG00000141497	*ZMYND15*	-1.604	0.00079	0.08113	-1.424	0.00169	0.109805
ENSG00000072657	*TRHDE*	-1.472	0.00192	0.13820	-1.424	0.00169	0.109805
ENSG00000108784	*NAGLU*	-0.817	0.00165	0.12688	-0.813	0.00169	0.109805
ENSG00000125354	*SEPTIN6*	-0.711	0.00294	0.18164	-0.737	0.00171	0.110048
ENSG00000184898	*RBM43*	-0.948	0.00202	0.14248	-0.952	0.00171	0.110048
ENSG00000183186	*C2CD4C*	-1.291	0.00222	0.15314	-1.321	0.00171	0.110048
ENSG00000107831	*FGF8*	1.327	0.00239	0.15984	1.339	0.00171	0.110048
ENSG00000187123	*LYPD6*	-0.856	0.00152	0.11976	-0.838	0.00171	0.110048
ENSG00000184897	*H1-10*	0.670	0.00169	0.12871	0.669	0.00171	0.110048
ENSG00000132563	*REEP2*	-1.714	0.00126	0.10751	-1.623	0.00172	0.110342
ENSG00000177565	*TBL1XR1*	-0.643	0.00188	0.13740	-0.648	0.00173	0.110611
ENSG00000180884	*ZNF792*	-1.026	0.00622	0.28294	-1.156	0.00174	0.111050
ENSG00000197380	*DACT3*	-1.159	0.00186	0.13689	-1.131	0.00174	0.111146
ENSG00000088367	*EPB41L1*	-0.669	0.00335	0.19700	-0.703	0.00174	0.111146
ENSG00000213853	*EMP2*	-0.668	0.00210	0.14692	-0.676	0.00176	0.111896
ENSG00000112977	*DAP*	-0.664	0.00174	0.13095	-0.662	0.00176	0.111896
ENSG00000235244	*DANT2*	0.770	0.00177	0.13253	0.761	0.00176	0.112148
ENSG00000240875	*LINC00886*	1.037	0.00180	0.13422	1.019	0.00177	0.112348
ENSG00000006062	*MAP3K14*	0.672	0.00190	0.13772	0.671	0.00177	0.112556
ENSG00000101974	*ATP11C*	-0.635	0.00267	0.17166	-0.657	0.00178	0.112882
ENSG00000175220	*ARHGAP1*	-0.627	0.00333	0.19615	-0.663	0.00179	0.113040
ENSG00000165731	*RET*	-1.466	0.00047	0.05625	-1.257	0.00179	0.113164
ENSG00000172594	*SMPDL3A*	-0.883	0.00344	0.19909	-0.898	0.00180	0.113862
ENSG00000170340	*B3GNT2*	-0.679	0.00190	0.13772	-0.680	0.00182	0.114522
ENSG00000154217	*PITPNC1*	-0.684	0.00242	0.16037	-0.687	0.00183	0.114657
ENSG00000162337	*LRP5*	-0.664	0.00201	0.14240	-0.665	0.00184	0.115117
ENSG00000232995	*RGS5*	0.831	0.00137	0.11197	0.801	0.00184	0.115117
ENSG00000148730	*EIF4EBP2*	-0.649	0.00181	0.13469	-0.648	0.00184	0.115117
ENSG00000163376	*KBTBD8*	-0.697	0.00160	0.12400	-0.688	0.00184	0.115296
ENSG00000197444	*OGDHL*	-0.885	0.00109	0.09927	-0.823	0.00186	0.115656
ENSG00000237105	*HCP5*	-1.020	0.00110	0.09972	-0.739	0.00187	0.116469
ENSG00000183688	*RFLNB*	-0.920	0.00083	0.08348	-0.753	0.00188	0.116882
ENSG00000230798	*FOXD3-AS1*	-1.836	0.00283	0.17677	-1.806	0.00189	0.117063
ENSG00000214954	*LRRC69*	0.768	0.00141	0.11374	0.736	0.00189	0.117140
ENSG00000166819	*PLIN1*	2.075	0.00189	0.13770	1.964	0.00191	0.117650
ENSG00000148488	*ST8SIA6*	-1.721	0.00616	0.28188	-1.893	0.00191	0.117650
ENSG00000260917	*LOC103344931*	0.718	0.00189	0.13770	0.717	0.00191	0.117656
ENSG00000101439	*CST3*	-0.706	0.00115	0.10286	-0.671	0.00191	0.117701
ENSG00000167536	*DHRS13*	-0.771	0.00402	0.21883	-0.822	0.00193	0.118507
ENSG00000252835	*SCARNA21*	-0.666	0.00303	0.18488	-0.693	0.00194	0.119017
ENSG00000119729	*RHOQ*	-0.626	0.00447	0.23032	-0.671	0.00194	0.119224
ENSG00000235437	*LINC01278*	0.784	0.00113	0.10155	0.740	0.00195	0.119229
ENSG00000175662	*TOM1L2*	0.649	0.00378	0.21135	0.687	0.00196	0.119763
ENSG00000173868	*PHOSPHO1*	-1.199	0.00749	0.31537	-1.354	0.00196	0.119763
ENSG00000068615	*REEP1*	-0.707	0.00234	0.15740	-0.713	0.00196	0.119763
ENSG00000164172	*MOCS2*	-0.642	0.00255	0.16678	-0.657	0.00196	0.119763
ENSG00000107537	*PHYH*	-0.752	0.00278	0.17545	-0.736	0.00198	0.120375
ENSG00000092964	*DPYSL2*	-0.636	0.00260	0.16904	-0.650	0.00200	0.121303
ENSG00000135324	*MRAP2*	-1.150	0.00270	0.17191	-1.169	0.00200	0.121356
ENSG00000177875	*CCDC184*	-2.886	0.00201	0.14239	-2.889	0.00201	0.121424
ENSG00000108342	*CSF3*	2.962	0.00201	0.14239	2.959	0.00201	0.121424
ENSG00000166183	*ASPG*	-1.431	0.00278	0.17545	-1.470	0.00204	0.122998
ENSG00000105173	*CCNE1*	-0.588	0.00632	0.28630	-0.657	0.00205	0.123175

ENSG00000260097	*SPDYE6*	0.766	0.00664	0.29219	0.690	0.00205	0.123375
ENSG00000170049	*KCNAB3*	0.803	0.00316	0.18961	0.821	0.00205	0.123464
ENSG00000218336	*TENM3*	-0.680	0.00158	0.12306	-0.660	0.00206	0.123468
ENSG00000138622	*HCN4*	-0.905	0.00279	0.17551	-0.921	0.00206	0.123690
ENSG00000243742	*RPLP0P2*	-0.767	0.00541	0.26042	-0.874	0.00212	0.126737
ENSG00000272610	*MAGI1-IT1*	1.094	0.00186	0.13689	1.071	0.00212	0.126737
ENSG00000037749	*MFAP3*	-0.632	0.00352	0.20097	-0.664	0.00213	0.126737
ENSG00000137364	*TPMT*	-0.641	0.00489	0.24380	-0.685	0.00213	0.126737
ENSG00000126821	*SGPP1*	-0.700	0.00233	0.15701	-0.668	0.00213	0.126737
ENSG00000228570	*NUTM2E*	0.870	0.00299	0.18333	0.709	0.00213	0.126763
ENSG00000116191	*RALGPS2*	-0.655	0.00272	0.17303	-0.667	0.00215	0.127515
ENSG00000257815	*CNOT2-DT*	1.111	0.00527	0.25533	1.202	0.00215	0.127651
ENSG00000166856	*GPR182*	1.169	0.00149	0.11820	1.166	0.00216	0.127816
ENSG00000013588	*GPRC5A*	-0.638	0.00213	0.14823	-0.636	0.00220	0.129801
ENSG00000198108	*CHSY3*	-0.959	0.00213	0.14823	-0.950	0.00220	0.129801
ENSG00000189350	*TOGARAM2*	-0.885	0.00149	0.11820	-0.817	0.00220	0.129899
ENSG00000171241	*SHCBP1*	-0.612	0.00350	0.20066	-0.639	0.00223	0.131348
ENSG00000105137	*SYDE1*	-0.896	0.00172	0.12947	-0.855	0.00224	0.131649
ENSG00000196878	*LAMB3*	0.647	0.00218	0.15070	0.643	0.00224	0.131649
ENSG00000187730	*GABRD*	-0.999	0.00161	0.12422	-0.918	0.00225	0.132052
ENSG00000248508	*SRP14-AS1*	0.853	0.00127	0.10751	0.792	0.00226	0.132582
ENSG00000131746	*TNS4*	-0.656	0.00180	0.13422	-0.640	0.00227	0.133109
ENSG00000165046	*LETM2*	-0.801	0.00218	0.15074	-0.779	0.00227	0.133109
ENSG00000203780	*FANK1*	0.973	0.00019	0.02870	0.773	0.00228	0.133125
ENSG00000205133	*TRIQK*	-0.666	0.00328	0.19406	-0.683	0.00230	0.133954
ENSG00000099139	*PCSK5*	-0.701	0.00208	0.14613	-0.683	0.00230	0.134155
ENSG00000136783	*NIPSNAP3A*	-0.744	0.00319	0.19125	-0.739	0.00231	0.134516
ENSG00000157087	*ATP2B2*	-1.256	0.00123	0.10656	-1.141	0.00232	0.134920
ENSG00000149418	*ST14*	-0.635	0.00236	0.15853	-0.634	0.00233	0.135054
ENSG00000183018	*SPNS2*	0.880	0.00060	0.06643	0.773	0.00233	0.135252
ENSG00000163110	*PDLIM5*	-0.613	0.00371	0.20847	-0.640	0.00234	0.135274
ENSG00000170545	*SMAGP*	-0.684	0.00205	0.14440	-0.672	0.00234	0.135274
ENSG00000122779	*TRIM24*	-0.584	0.00592	0.27499	-0.642	0.00236	0.135987
ENSG00000117899	*MESD*	-0.622	0.00310	0.18726	-0.639	0.00237	0.136092
ENSG00000155189	*AGPAT5*	-0.648	0.00226	0.15453	-0.633	0.00237	0.136092
ENSG00000203883	*SOX18*	-1.008	0.00128	0.10751	-0.918	0.00238	0.136655
ENSG00000163349	*HIPK1*	-0.626	0.00264	0.17118	-0.631	0.00239	0.136915
ENSG00000148848	*ADAM12*	0.975	0.00337	0.19758	1.003	0.00239	0.137030
ENSG00000105784	*RUNDC3B*	-0.741	0.00431	0.22662	-0.779	0.00242	0.137965
ENSG00000239264	*TXNDC5*	-0.812	0.00173	0.13032	-0.791	0.00242	0.137965
ENSG00000175967	*FLJ34503*	6.359	0.00242	0.16037	6.358	0.00242	0.137965
ENSG00000196196	*HRCT1*	6.359	0.00242	0.16037	6.358	0.00242	0.137965
ENSG00000152931	*PART1*	3.049	0.00119	0.10447	2.571	0.00243	0.137983
ENSG00000165996	*HACD1*	-0.768	0.00202	0.14269	-0.737	0.00244	0.138413
ENSG00000115839	*RAB3GAP1*	-0.647	0.00206	0.14467	-0.633	0.00245	0.138945
ENSG00000118707	*TGIF2*	-0.640	0.00299	0.18330	-0.652	0.00245	0.138945
ENSG00000163623	*NKX6-1*	-1.124	0.00163	0.12589	-1.045	0.00246	0.138945
ENSG00000113763	*UNC5A*	-1.092	0.00258	0.16774	-1.045	0.00246	0.138945
ENSG00000186854	*TRABD2A*	-0.959	0.00190	0.13772	-0.927	0.00246	0.139087
ENSG00000223839	*FAM95B1*	0.765	0.00888	0.34775	0.708	0.00248	0.139757
ENSG00000261924	*LOC400627*	0.961	0.00227	0.15456	0.946	0.00248	0.139757
ENSG00000112414	*ADGRG6*	-0.656	0.00210	0.14721	-0.641	0.00251	0.140524
ENSG00000198478	*SH3BGRL2*	-0.680	0.00227	0.15456	-0.668	0.00251	0.140524
ENSG00000187867	*PALM3*	-0.775	0.00207	0.14511	-0.752	0.00251	0.140524
ENSG00000165548	*TMEM63C*	-0.754	0.00342	0.19844	-0.754	0.00252	0.140820
ENSG00000147394	*ZNF185*	-0.685	0.00478	0.23950	-0.715	0.00254	0.141614

ENSG00000122694	*GLIPR2*	-0.717	0.00253	0.16582	-0.703	0.00255	0.141948
ENSG00000182040	*USH1G*	-0.793	0.00396	0.21729	-0.821	0.00255	0.141948
ENSG00000138678	*GPAT3*	-0.615	0.00416	0.22368	-0.643	0.00256	0.142079
ENSG00000140807	*NKD1*	-0.665	0.00293	0.18126	-0.671	0.00256	0.142079
ENSG00000163466	*ARPC2*	-0.601	0.00386	0.21375	-0.625	0.00256	0.142079
ENSG00000235823	*OLMALINC*	0.720	0.00156	0.12233	0.680	0.00260	0.143094
ENSG00000177707	*NECTIN3*	-0.612	0.00430	0.22662	-0.644	0.00260	0.143187
ENSG00000049759	*NEDD4L*	-0.619	0.00411	0.22214	-0.642	0.00261	0.143430
ENSG00000103241	*FOXF1*	-0.887	0.00400	0.21863	-0.927	0.00261	0.143556
ENSG00000010017	*RANBP9*	-0.611	0.00365	0.20604	-0.630	0.00262	0.143828
ENSG00000100150	*DEPDC5*	0.702	0.00135	0.11096	0.652	0.00262	0.143828
ENSG00000132589	*FLOT2*	-0.585	0.00601	0.27673	-0.637	0.00263	0.144364
ENSG00000165169	*DYNLT3*	-0.687	0.00178	0.13346	-0.656	0.00264	0.144656
ENSG00000159399	*HK2*	-0.632	0.00265	0.17149	-0.630	0.00264	0.144823
ENSG00000170035	*UBE2E3*	-0.618	0.00425	0.22539	-0.637	0.00266	0.145284
ENSG00000248592	*STIMATE-MUSTN1*	1.713	0.00382	0.21185	1.759	0.00266	0.145284
ENSG00000108443	*RPS6KB1*	-0.584	0.00507	0.24896	-0.624	0.00267	0.145284
ENSG00000134013	*LOXL2*	-0.764	0.00209	0.14663	-0.728	0.00268	0.145284
ENSG00000136449	*MYCBPAP*	2.406	0.00639	0.28745	2.322	0.00268	0.145284
ENSG00000114113	*RBP2*	2.174	0.00665	0.29219	2.322	0.00268	0.145284
ENSG00000198342	*ZNF442*	-2.589	0.00961	0.36180	-2.251	0.00268	0.145284
ENSG00000140718	*FTO*	0.685	0.00158	0.12306	0.648	0.00269	0.145477
ENSG00000099337	*KCNK6*	-0.699	0.00753	0.31645	-0.783	0.00273	0.147017
ENSG00000071537	*SEL1L*	-0.606	0.00378	0.21130	-0.625	0.00277	0.148583
ENSG00000150457	*LATS2*	-0.612	0.00437	0.22787	-0.643	0.00278	0.148946
ENSG00000164070	*HSPA4L*	-0.594	0.00443	0.22940	-0.622	0.00280	0.149680
ENSG00000109920	*FNBP4*	0.631	0.00252	0.16536	0.617	0.00282	0.150823
ENSG00000198590	*C3orf35*	0.766	0.00354	0.20172	0.772	0.00283	0.151174
ENSG00000127124	*HIVEP3*	-0.680	0.00238	0.15961	-0.666	0.00285	0.151473
ENSG00000274897	*PANO1*	-1.939	0.00285	0.17715	-1.942	0.00285	0.151473
ENSG00000141579	*ZNF750*	0.824	0.00402	0.21889	0.850	0.00286	0.151518
ENSG00000245680	*ZNF585B*	-0.707	0.00359	0.20368	-0.708	0.00287	0.152270
ENSG00000087448	*KLHL42*	-0.606	0.00407	0.22094	-0.628	0.00288	0.152270
ENSG00000153253	*SCN3A*	-0.974	0.00303	0.18487	-0.937	0.00288	0.152270
ENSG00000104325	*DECR1*	-0.646	0.00295	0.18187	-0.637	0.00288	0.152270
ENSG00000128274	*A4GALT*	-0.836	0.00266	0.17149	-0.821	0.00290	0.152993
ENSG00000112514	*CUTA*	0.630	0.00550	0.26271	0.635	0.00293	0.153685
ENSG00000065154	*OAT*	-0.592	0.00450	0.23155	-0.618	0.00293	0.153685
ENSG00000166343	*MSS51*	0.831	0.00304	0.18491	0.826	0.00293	0.153685
ENSG00000162344	*FGF19*	0.671	0.00265	0.17120	0.662	0.00293	0.153685
ENSG00000170881	*RNF139*	-0.623	0.00326	0.19347	-0.629	0.00293	0.153685
ENSG00000248008	*NRAV*	0.655	0.00422	0.22500	0.681	0.00294	0.153955
ENSG00000173083	*HPSE*	0.688	0.00323	0.19303	0.681	0.00295	0.154528
ENSG00000136295	*TTYH3*	-0.623	0.00411	0.22214	-0.644	0.00295	0.154550
ENSG00000158186	*MRAS*	-0.617	0.00576	0.26979	-0.657	0.00296	0.154714
ENSG00000063587	*ZNF275*	-0.640	0.00290	0.17974	-0.639	0.00298	0.155561
ENSG00000163482	*STK36*	0.600	0.00486	0.24249	0.630	0.00298	0.155640
ENSG00000006747	*SCIN*	1.447	0.00350	0.20066	1.401	0.00300	0.156176
ENSG00000112697	*TMEM30A*	-0.612	0.00324	0.19303	-0.616	0.00301	0.156766
ENSG00000161270	*NPHS1*	0.670	0.00532	0.25711	0.701	0.00302	0.156880
ENSG00000171227	*TMEM37*	-0.944	0.00418	0.22398	-0.977	0.00303	0.157172
ENSG00000127824	*TUBA4A*	-0.658	0.00231	0.15638	-0.639	0.00304	0.157786
ENSG00000282230	*ADAM9*	-0.689	0.00150	0.11827	-0.624	0.00304	0.157796
ENSG00000164209	*SLC25A46*	-0.623	0.00317	0.19024	-0.624	0.00306	0.158290
ENSG00000138316	*ADAMTS14*	-1.005	0.00837	0.33478	-1.090	0.00306	0.158290
ENSG00000111961	*SASH1*	-0.642	0.00390	0.21527	-0.652	0.00306	0.158290

ENSG00000145569	*OTULINL*	-0.741	0.00350	0.20066	-0.739	0.00306	0.158290
ENSG00000107984	*DKK1*	-0.609	0.00309	0.18676	-0.609	0.00307	0.158590
ENSG00000147526	*TACC1*	-0.609	0.00353	0.20123	-0.617	0.00308	0.158957
ENSG00000155893	*PXYLP1*	-0.666	0.00284	0.17715	-0.658	0.00309	0.159025
ENSG00000156256	*USP16*	-0.578	0.00606	0.27808	-0.620	0.00311	0.160116
ENSG00000160058	*BSDC1*	0.660	0.00187	0.13728	0.623	0.00311	0.160116
ENSG00000094631	*HDAC6*	0.631	0.00275	0.17464	0.620	0.00313	0.160504
ENSG00000138772	*ANXA3*	-0.605	0.00374	0.20995	-0.613	0.00313	0.160504
ENSG00000169255	*B3GALNT1*	-0.687	0.00186	0.13678	-0.648	0.00313	0.160504
ENSG00000206384	*COL6A6*	0.931	0.00482	0.24130	0.973	0.00314	0.160642
ENSG00000275342	*PRAG1*	-0.631	0.00340	0.19781	-0.621	0.00314	0.160642
ENSG00000090776	*EFNB1*	-0.666	0.00323	0.19303	-0.661	0.00314	0.160642
ENSG00000197956	*S100A6*	0.599	0.00366	0.20625	0.608	0.00316	0.161168
ENSG00000169946	*ZFPM2*	0.708	0.00238	0.15961	0.683	0.00321	0.163316
ENSG00000148925	*BTBD10*	-0.668	0.00394	0.21680	-0.670	0.00325	0.164562
ENSG00000076706	*MCAM*	-0.619	0.00568	0.26897	-0.650	0.00325	0.164562
ENSG00000117266	*CDK18*	-0.692	0.00584	0.27189	-0.722	0.00327	0.164562
ENSG00000151718	*WWC2*	-0.604	0.00409	0.22137	-0.615	0.00331	0.166246
ENSG00000055163	*CYFIP2*	-0.688	0.00428	0.22621	-0.689	0.00333	0.167362
ENSG00000152767	*FARP1*	-0.594	0.00507	0.24896	-0.620	0.00337	0.168981
ENSG00000150782	*IL18*	1.836	0.00031	0.04005	1.306	0.00338	0.168981
ENSG00000183161	*FANCF*	-1.302	0.00109	0.09927	-1.130	0.00338	0.168981
ENSG00000282556	*LOC101927825*	6.734	0.00035	0.04482	2.866	0.00339	0.168981
ENSG00000188886	*ASTL*	3.343	0.00788	0.32074	2.866	0.00339	0.168981
ENSG00000207780	*MIR648*	2.665	0.00961	0.36180	2.866	0.00339	0.168981
ENSG00000104219	*ZDHHC2*	-0.632	0.00394	0.21680	-0.634	0.00340	0.168981
ENSG00000170381	*SEMA3E*	-1.007	0.00304	0.18509	-0.948	0.00341	0.169478
ENSG00000110799	*VWF*	1.539	0.00298	0.18310	1.195	0.00342	0.169633
ENSG00000234840	*LINC01239*	1.227	0.00481	0.24098	1.195	0.00342	0.169633
ENSG00000038210	*PI4K2B*	-0.635	0.00376	0.21028	-0.640	0.00342	0.169633
ENSG00000153064	*BANK1*	-0.864	0.00689	0.30046	-0.875	0.00344	0.169934
ENSG00000055208	*TAB2*	0.612	0.00337	0.19758	0.610	0.00344	0.170140
ENSG00000142089	*IFITM3*	-0.642	0.00311	0.18771	-0.629	0.00345	0.170140
ENSG00000171992	*SYNPO*	-1.016	0.00513	0.25083	-1.056	0.00352	0.173318
ENSG00000169504	*CLIC4*	-0.635	0.00349	0.20066	-0.631	0.00353	0.173449
ENSG00000275246	*TLCD2*	-0.909	0.00363	0.20520	-0.776	0.00356	0.175153
ENSG00000078687	*TNRC6C*	-0.612	0.00375	0.21028	-0.613	0.00357	0.175374
ENSG00000240498	*CDKN2B-AS1*	0.702	0.00326	0.19347	0.689	0.00357	0.175374
ENSG00000196116	*TDRD7*	-0.679	0.00491	0.24434	-0.683	0.00359	0.176166
ENSG00000160285	*LSS*	0.600	0.00520	0.25302	0.610	0.00360	0.176657
ENSG00000152642	*GPD1L*	-0.632	0.00349	0.20066	-0.629	0.00362	0.177044
ENSG00000149260	*CAPN5*	-0.721	0.00570	0.26897	-0.740	0.00364	0.177544
ENSG00000164125	*GASK1B*	-0.786	0.00971	0.36368	-0.874	0.00364	0.177796
ENSG00000151208	*DLG5*	-0.570	0.00777	0.32074	-0.607	0.00365	0.177796
ENSG00000088340	*FER1L4*	0.769	0.00308	0.18660	0.739	0.00365	0.177796
ENSG00000120738	*EGR1*	-0.596	0.00422	0.22500	-0.605	0.00366	0.178112
ENSG00000123685	*BATF3*	-0.816	0.00496	0.24619	-0.813	0.00369	0.179327
ENSG00000149823	*VPS51*	-0.617	0.00378	0.21130	-0.615	0.00371	0.180085
ENSG00000104369	*JPH1*	-0.614	0.00433	0.22662	-0.622	0.00374	0.181036
ENSG00000137563	*GGH*	-0.608	0.00446	0.22991	-0.612	0.00374	0.181036
ENSG00000137860	*SLC28A2*	0.792	0.00267	0.17166	0.755	0.00375	0.181329
ENSG00000183840	*GPR39*	0.940	0.00255	0.16658	0.893	0.00376	0.181645
ENSG00000091409	*ITGA6*	-0.582	0.00502	0.24792	-0.599	0.00377	0.182137
ENSG00000232656	*IDI2-AS1*	1.500	0.00408	0.22120	1.468	0.00378	0.182144
ENSG00000163884	*KLF15*	-1.942	0.00128	0.10751	-1.639	0.00382	0.183444
ENSG00000228290	*TBX18-AS1*	1.713	0.00382	0.21185	1.710	0.00382	0.183444

ENSG00000126861	*OMG*	0.780	0.00263	0.17036	0.745	0.00383	0.183651
ENSG00000121101	*TEX14*	0.861	0.00085	0.08447	0.705	0.00385	0.184347
ENSG00000074211	*PPP2R2C*	-0.601	0.00646	0.28959	-0.634	0.00386	0.184745
ENSG00000222047	*C10orf55*	1.183	0.00270	0.17191	1.129	0.00389	0.185616
ENSG00000106688	*SLC1A1*	-0.749	0.00989	0.36766	-0.801	0.00394	0.187234
ENSG00000111700	*SLCO1B3*	-0.740	0.00339	0.19758	-0.730	0.00394	0.187234
ENSG00000167535	*CACNB3*	-0.653	0.00559	0.26642	-0.668	0.00394	0.187234
ENSG00000204334	*ERICH2*	-1.421	0.00169	0.12871	-1.044	0.00395	0.187234
ENSG00000137193	*PIM1*	0.643	0.00575	0.26979	0.665	0.00396	0.187604
ENSG00000280852	*LOC653653*	-0.741	0.00525	0.25516	-0.726	0.00396	0.187604
ENSG00000049130	*KITLG*	-0.602	0.00351	0.20070	-0.593	0.00399	0.188651
ENSG00000281026	*N4BP2L2-IT2*	0.620	0.00521	0.25319	0.640	0.00401	0.188923
ENSG00000127954	*STEAP4*	1.737	0.00904	0.34944	1.775	0.00402	0.188923
ENSG00000164465	*DCBLD1*	-0.634	0.00401	0.21883	-0.625	0.00407	0.190452
ENSG00000196967	*ZNF585A*	-0.704	0.00476	0.23892	-0.688	0.00408	0.190801
ENSG00000181826	*RELL1*	-0.699	0.00332	0.19587	-0.670	0.00409	0.191032
ENSG00000204335	*SP5*	-0.769	0.00495	0.24574	-0.771	0.00410	0.191346
ENSG00000174791	*RIN1*	-0.641	0.00404	0.21953	-0.636	0.00413	0.192461
ENSG00000137834	*SMAD6*	0.894	0.00164	0.12623	0.805	0.00414	0.192747
ENSG00000132155	*RAF1*	-0.560	0.00745	0.31498	-0.598	0.00416	0.193617
ENSG00000203896	*LIME1*	0.936	0.00191	0.13772	0.869	0.00416	0.193617
ENSG00000169860	*P2RY1*	-1.779	0.00418	0.22398	-1.782	0.00418	0.194201
ENSG00000147130	*ZMYM3*	-0.612	0.00389	0.21504	-0.604	0.00420	0.194613
ENSG00000263247	*PRH1*	0.920	0.00167	0.12813	0.681	0.00420	0.194751
ENSG00000100652	*SLC10A1*	0.724	0.00471	0.23719	0.730	0.00421	0.194840
ENSG00000075618	*FSCN1*	-0.574	0.00543	0.26079	-0.590	0.00422	0.194986
ENSG00000162430	*SELENON*	-0.602	0.00428	0.22603	-0.602	0.00425	0.195966
ENSG00000236756	*DNAJC9-AS1*	0.959	0.00672	0.29459	0.996	0.00425	0.195966
ENSG00000173531	*MST1*	0.829	0.00072	0.07609	0.661	0.00428	0.196724
ENSG00000275491	*LINC01730*	1.458	0.00180	0.13422	1.387	0.00432	0.197993
ENSG00000125841	*NRSN2*	2.012	0.00663	0.29219	2.079	0.00433	0.197993
ENSG00000163485	*ADORA1*	-0.901	0.00583	0.27144	-0.921	0.00434	0.198639
ENSG00000196208	*GREB1*	-0.647	0.00288	0.17849	-0.616	0.00437	0.199619
ENSG00000244161	*FLNB-AS1*	0.746	0.00499	0.24687	0.758	0.00438	0.199782
ENSG00000118997	*DNAH7*	1.523	0.00661	0.29219	1.529	0.00440	0.200777
ENSG00000100439	*ABHD4*	0.632	0.00543	0.26079	0.645	0.00441	0.200777
ENSG00000214694	*ARHGEF33*	1.197	0.00619	0.28240	1.216	0.00441	0.200777
ENSG00000075290	*WNT8B*	0.833	0.00577	0.27004	0.866	0.00442	0.200785
ENSG00000054598	*FOXC1*	-0.664	0.00515	0.25165	-0.677	0.00443	0.200785
ENSG00000134970	*TMED7*	-0.589	0.00492	0.24468	-0.596	0.00443	0.200785
ENSG00000175029	*CTBP2*	-0.560	0.00757	0.31707	-0.594	0.00443	0.200785
ENSG00000183876	*ARSI*	-3.393	0.00444	0.22940	-3.396	0.00444	0.200785
ENSG00000046653	*GPM6B*	0.715	0.00620	0.28240	0.739	0.00445	0.200957
ENSG00000266265	*KLF14*	-0.913	0.00620	0.28240	-0.947	0.00449	0.202524
ENSG00000122884	*P4HA1*	-0.592	0.00539	0.25960	-0.600	0.00449	0.202542
ENSG00000174938	*SEZ6L2*	-0.608	0.00572	0.26897	-0.618	0.00450	0.202915
ENSG00000225828	*FAM229A*	0.877	0.00223	0.15327	0.807	0.00453	0.203774
ENSG00000183283	*DAZAP2*	-0.609	0.00361	0.20415	-0.592	0.00456	0.204702
ENSG00000157510	*AFAP1L1*	-0.677	0.00821	0.33009	-0.711	0.00458	0.205191
ENSG00000171016	*PYGO1*	-0.771	0.00436	0.22764	-0.758	0.00458	0.205191
ENSG00000125462	*C1orf61*	0.607	0.00431	0.22662	0.603	0.00459	0.205191
ENSG00000158423	*RIBC1*	1.173	0.00433	0.22662	1.135	0.00460	0.205191
ENSG00000144647	*POMGNT2*	-0.611	0.00518	0.25252	-0.618	0.00460	0.205191
ENSG00000136944	*LMX1B*	-0.682	0.00244	0.16101	-0.633	0.00462	0.205191
ENSG00000184208	*C22orf46*	0.646	0.00327	0.19347	0.623	0.00463	0.205191
ENSG00000167702	*KIFC2*	0.718	0.00123	0.10669	0.623	0.00463	0.205191

ENSG00000162433	*AK4*	-0.572	0.00673	0.29477	-0.591	0.00463	0.205191
ENSG00000251143	*LOC100128494*	1.082	0.00505	0.24850	1.077	0.00464	0.205191
ENSG00000184502	*GAST*	6.209	0.00464	0.23500	6.208	0.00464	0.205191
ENSG00000198807	*PAX9*	-0.652	0.00731	0.31120	-0.684	0.00465	0.205249
ENSG00000124615	*MOCS1*	-0.737	0.00572	0.26897	-0.731	0.00469	0.206972
ENSG00000143515	*ATP8B2*	-0.603	0.00531	0.25687	-0.606	0.00470	0.207240
ENSG00000161011	*SQSTM1*	0.603	0.00384	0.21292	0.583	0.00470	0.207240
ENSG00000129355	*CDKN2D*	0.700	0.00660	0.29219	0.717	0.00473	0.207593
ENSG00000149948	*HMGA2*	-0.562	0.00657	0.29219	-0.584	0.00473	0.207593
ENSG00000229544	*NKX1-2*	-1.491	0.00474	0.23844	-1.494	0.00474	0.208112
ENSG00000040608	*RTN4R*	-0.697	0.00424	0.22510	-0.690	0.00477	0.209325
ENSG00000168118	*RAB4A*	-0.554	0.00965	0.36222	-0.598	0.00480	0.210151
ENSG00000146592	*CREB5*	1.425	0.00242	0.16037	1.320	0.00482	0.210740
ENSG00000153006	*SREK1IP1*	-0.636	0.00316	0.18961	-0.603	0.00487	0.212716
ENSG00000173065	*FAM222B*	-0.607	0.00543	0.26079	-0.613	0.00488	0.212716
ENSG00000122068	*FYTTD1*	-0.595	0.00416	0.22368	-0.584	0.00489	0.213081
ENSG00000004848	*ARX*	-0.987	0.00226	0.15451	-0.876	0.00491	0.213644
ENSG00000146197	*SCUBE3*	0.668	0.00359	0.20368	0.648	0.00493	0.213932
ENSG00000183873	*SCN5A*	-0.641	0.00575	0.26979	-0.644	0.00493	0.213932
ENSG00000112972	*HMGCS1*	0.584	0.00461	0.23500	0.579	0.00493	0.213932
ENSG00000105810	*CDK6*	-0.579	0.00506	0.24896	-0.580	0.00494	0.214423
ENSG00000170500	*LONRF2*	0.996	0.00541	0.26048	0.994	0.00499	0.216106
ENSG00000136908	*DPM2*	0.636	0.00351	0.20072	0.608	0.00499	0.216122
ENSG00000164402	*SEPTIN8*	-0.584	0.00637	0.28745	-0.597	0.00503	0.217324
ENSG00000121552	*CSTA*	-0.826	0.00312	0.18780	-0.746	0.00503	0.217324
ENSG00000240204	*SMKR1*	-1.202	0.00184	0.13559	-1.051	0.00505	0.217605
ENSG00000148200	*NR6A1*	0.652	0.00359	0.20368	0.624	0.00506	0.217881
ENSG00000189334	*S100A14*	0.593	0.00599	0.27617	0.602	0.00507	0.218219
ENSG00000077800	*FKBP6*	1.132	0.00389	0.21504	0.794	0.00508	0.218219
ENSG00000277150	*F8A3*	-1.094	0.00690	0.30055	-0.791	0.00508	0.218219
ENSG00000175643	*RMI2*	-0.664	0.00811	0.32687	-0.697	0.00508	0.218219
ENSG00000123933	*MXD4*	-0.606	0.00722	0.30863	-0.627	0.00510	0.218413
ENSG00000262185	*LINC02861*	-1.590	0.00841	0.33546	-1.537	0.00510	0.218413
ENSG00000163395	*IGFN1*	1.737	0.00904	0.34944	1.609	0.00510	0.218413
ENSG00000179361	*ARID3B*	0.648	0.00353	0.20104	0.610	0.00521	0.222643
ENSG00000125398	*SOX9*	-0.594	0.00542	0.26071	-0.596	0.00522	0.222796
ENSG00000140848	*CPNE2*	-0.589	0.00767	0.31924	-0.605	0.00522	0.222815
ENSG00000198794	*SCAMP5*	-0.733	0.00419	0.22398	-0.704	0.00526	0.224089
ENSG00000142347	*MYO1F*	1.024	0.00139	0.11326	0.865	0.00529	0.225027
ENSG00000103642	*LACTB*	-0.635	0.00741	0.31439	-0.652	0.00532	0.225859
ENSG00000160753	*RUSC1*	0.577	0.00725	0.30934	0.594	0.00539	0.228445
ENSG00000197632	*SERPINB2*	-1.533	0.00779	0.32074	-1.588	0.00546	0.230643
ENSG00000154479	*CCDC173*	0.936	0.00883	0.34694	0.935	0.00549	0.231184
ENSG00000280351	*LOC100129503*	0.877	0.00324	0.19303	0.817	0.00551	0.231779
ENSG00000184900	*SUMO3*	-0.591	0.00597	0.27608	-0.594	0.00554	0.232669
ENSG00000176473	*WDR25*	0.663	0.00897	0.34811	0.694	0.00560	0.234869
ENSG00000126091	*ST3GAL3*	0.692	0.00415	0.22368	0.666	0.00560	0.234869
ENSG00000161912	*ADCY10P1*	0.927	0.00398	0.21800	0.869	0.00560	0.234869
ENSG00000197385	*ZNF860*	-0.673	0.00746	0.31508	-0.693	0.00563	0.235709
ENSG00000168263	*KCNV2*	0.849	0.00696	0.30136	0.862	0.00565	0.236157
ENSG00000136111	*TBC1D4*	-0.590	0.00490	0.24380	-0.579	0.00565	0.236157
ENSG00000226180	*LOC100129215*	0.701	0.00634	0.28677	0.705	0.00566	0.236473
ENSG00000165801	*ARHGEF40*	-0.769	0.00456	0.23309	-0.734	0.00571	0.237568
ENSG00000277897	*GSTT2*	-1.293	0.00526	0.25533	-0.736	0.00572	0.237568
ENSG00000019549	*SNAI2*	0.648	0.00465	0.23504	0.627	0.00573	0.237568
ENSG00000125637	*PSD4*	-0.692	0.00451	0.23192	-0.662	0.00575	0.237828

ENSG00000232684	*ATP11A-AS1*	1.088	0.00719	0.30778	1.110	0.00575	0.237828
ENSG00000124225	*PMEPA1*	1.063	0.00987	0.36710	1.110	0.00575	0.237828
ENSG00000145703	*IQGAP2*	-1.355	0.00512	0.25059	-1.203	0.00576	0.237828
ENSG00000159588	*CCDC17*	0.864	0.00437	0.22793	0.842	0.00577	0.238065
ENSG00000136193	*SCRN1*	-0.571	0.00582	0.27144	-0.571	0.00583	0.239701
ENSG00000125351	*UPF3B*	0.585	0.00611	0.27996	0.584	0.00588	0.241575
ENSG00000130956	*HABP4*	0.675	0.00395	0.21680	0.630	0.00589	0.241657
ENSG00000197702	*PARVA*	-0.567	0.00744	0.31468	-0.580	0.00592	0.242255
ENSG00000131773	*KHDRBS3*	-0.596	0.00922	0.35316	-0.616	0.00593	0.242390
ENSG00000172139	*SLC9C1*	1.037	0.00291	0.17999	0.793	0.00594	0.242507
ENSG00000149212	*SESN3*	-0.917	0.00465	0.23504	-0.882	0.00595	0.242643
ENSG00000162976	*SLC66A3*	-0.726	0.00753	0.31645	-0.735	0.00596	0.243117
ENSG00000153814	*JAZF1*	-0.790	0.00380	0.21185	-0.743	0.00599	0.243893
ENSG00000050438	*SLC4A8*	-0.928	0.00568	0.26897	-0.871	0.00600	0.243893
ENSG00000115257	*PCSK4*	0.830	0.00992	0.36824	0.866	0.00600	0.243893
ENSG00000180638	*SLC47A2*	1.061	0.00743	0.31468	1.082	0.00600	0.243893
ENSG00000088538	*DOCK3*	0.590	0.00784	0.32074	0.599	0.00608	0.246737
ENSG00000104765	*BNIP3L*	-0.625	0.00494	0.24554	-0.606	0.00610	0.247359
ENSG00000162852	*CNST*	-0.624	0.00369	0.20771	-0.585	0.00610	0.247359
ENSG00000152402	*GUCY1A2*	1.737	0.00904	0.34944	1.795	0.00616	0.248866
ENSG00000186907	*RTN4RL2*	-0.665	0.00865	0.34115	-0.683	0.00621	0.250254
ENSG00000197147	*LRRC8B*	-0.574	0.00651	0.29054	-0.577	0.00623	0.250676
ENSG00000122707	*RECK*	-0.725	0.00805	0.32553	-0.729	0.00623	0.250676
ENSG00000169372	*CRADD*	-0.720	0.00439	0.22844	-0.686	0.00624	0.250689
ENSG00000260630	*SNAI3-AS1*	0.725	0.00758	0.31707	0.727	0.00624	0.250689
ENSG00000101577	*LPIN2*	-0.585	0.00712	0.30639	-0.592	0.00626	0.250871
ENSG00000139192	*TAPBPL*	0.797	0.00664	0.29219	0.797	0.00626	0.250871
ENSG00000100156	*SLC16A8*	0.778	0.00517	0.25191	0.751	0.00628	0.251187
ENSG00000134940	*ACRV1*	0.772	0.00789	0.32088	0.789	0.00628	0.251187
ENSG00000165072	*MAMDC2*	1.394	0.00692	0.30066	1.370	0.00631	0.251405
ENSG00000184933	*OR6A2*	1.335	0.00843	0.33584	1.370	0.00631	0.251405
ENSG00000185758	*CLDN24*	-1.731	0.00185	0.13613	-1.215	0.00632	0.251485
ENSG00000142765	*SYTL1*	0.647	0.00464	0.23500	0.615	0.00633	0.251566
ENSG00000148468	*FAM171A1*	-0.578	0.00615	0.28171	-0.574	0.00638	0.252441
ENSG00000130479	*MAP1S*	-0.596	0.00528	0.25582	-0.581	0.00639	0.252441
ENSG00000162621	*LRRC53*	2.406	0.00639	0.28745	2.403	0.00639	0.252441
ENSG00000263597	*MIR3936*	2.406	0.00639	0.28745	2.403	0.00639	0.252441
ENSG00000248485	*PCP4L1*	2.406	0.00639	0.28745	2.403	0.00639	0.252441
ENSG00000197774	*EME2*	0.604	0.00497	0.24639	0.586	0.00643	0.253546
ENSG00000095585	*BLNK*	1.825	0.00963	0.36180	1.889	0.00644	0.253546
ENSG00000214900	*LINC01588*	0.888	0.00333	0.19615	0.819	0.00661	0.257459
ENSG00000171488	*LRRC8C*	-0.575	0.00763	0.31825	-0.584	0.00662	0.257459
ENSG00000122861	*PLAU*	0.562	0.00667	0.29300	0.562	0.00663	0.257459
ENSG00000235849	*HS6ST2-AS1*	2.174	0.00665	0.29219	2.171	0.00665	0.257459
ENSG00000171867	*PRNP*	-0.572	0.00613	0.28069	-0.566	0.00667	0.257712
ENSG00000172380	*GNG12*	-0.553	0.00797	0.32284	-0.565	0.00668	0.258009
ENSG00000171130	*ATP6V0E2*	-0.588	0.00979	0.36509	-0.612	0.00668	0.258009
ENSG00000131620	*ANO1*	-0.644	0.00441	0.22866	-0.601	0.00670	0.258446
ENSG00000124787	*RPP40*	-0.665	0.00403	0.21928	-0.610	0.00673	0.259560
ENSG00000196739	*COL27A1*	-0.696	0.00244	0.16126	-0.607	0.00675	0.259926
ENSG00000118600	*RXYLT1*	-0.589	0.00730	0.31104	-0.589	0.00679	0.261152
ENSG00000130702	*LAMA5*	0.557	0.00716	0.30721	0.559	0.00682	0.261777
ENSG00000119514	*GALNT12*	-0.739	0.00432	0.22662	-0.676	0.00688	0.263889
ENSG00000100030	*MAPK1*	-0.549	0.00799	0.32317	-0.559	0.00691	0.264027
ENSG00000128591	*FLNC*	-1.578	0.00242	0.16037	-1.319	0.00692	0.264027
ENSG00000184261	*KCNK12*	1.394	0.00692	0.30066	1.391	0.00692	0.264027

ENSG00000108551	*RASD1*	1.394	0.00692	0.30066	1.391	0.00692	0.264027
ENSG00000184465	*WDR27*	0.564	0.00779	0.32074	0.571	0.00693	0.264209
ENSG00000181444	*ZNF467*	-1.276	0.00576	0.26979	-1.228	0.00694	0.264238
ENSG00000183255	*PTTG1IP*	-0.574	0.00596	0.27577	-0.563	0.00696	0.264280
ENSG00000103995	*CEP152*	0.601	0.00417	0.22398	0.564	0.00697	0.264280
ENSG00000016082	*ISL1*	-0.826	0.00359	0.20368	-0.751	0.00698	0.264280
ENSG00000155099	*PIP4P2*	-0.662	0.00634	0.28677	-0.646	0.00699	0.264280
ENSG00000113396	*SLC27A6*	-0.644	0.00594	0.27560	-0.622	0.00704	0.265464
ENSG00000145391	*SETD7*	-0.550	0.00787	0.32074	-0.557	0.00705	0.265569
ENSG00000102393	*GLA*	0.577	0.00760	0.31722	0.579	0.00706	0.265858
ENSG00000152078	*TLCD4*	-0.593	0.00716	0.30721	-0.592	0.00711	0.266580
ENSG00000250365	*LOC101927124*	0.970	0.00419	0.22398	0.903	0.00714	0.266580
ENSG00000089693	*MLF2*	-0.552	0.00795	0.32260	-0.558	0.00715	0.266580
ENSG00000151612	*ZNF827*	-0.552	0.00935	0.35691	-0.570	0.00715	0.266580
ENSG00000141458	*NPC1*	0.549	0.00900	0.34857	0.563	0.00715	0.266580
ENSG00000176845	*METRNL*	-0.677	0.00434	0.22662	-0.590	0.00716	0.266580
ENSG00000165197	*VEGFD*	1.913	0.00283	0.17677	1.560	0.00718	0.266580
ENSG00000091879	*ANGPT2*	0.652	0.00721	0.30850	0.649	0.00719	0.266580
ENSG00000166578	*IQCD*	0.764	0.00517	0.25191	0.736	0.00720	0.266580
ENSG00000274073	*TFPT*	2.082	0.00433	0.22662	0.694	0.00720	0.266580
ENSG00000260105	*AOC4P*	1.193	0.00316	0.18961	0.892	0.00721	0.266583
ENSG00000119630	*PGF*	-0.685	0.00645	0.28908	-0.664	0.00723	0.267230
ENSG00000205913	*SRRM2-AS1*	0.827	0.00307	0.18607	0.740	0.00724	0.267560
ENSG00000160094	*ZNF362*	-0.648	0.00649	0.28982	-0.632	0.00727	0.268507
ENSG00000130208	*APOC1*	-0.847	0.00217	0.15040	-0.712	0.00730	0.269365
ENSG00000114978	*MOB1A*	-0.563	0.00655	0.29177	-0.555	0.00731	0.269446
ENSG00000141429	*GALNT1*	-0.551	0.00814	0.32767	-0.557	0.00738	0.271148
ENSG00000145990	*GFOD1*	-0.634	0.00748	0.31537	-0.629	0.00739	0.271372
ENSG00000198886	*ND4*	0.554	0.00689	0.30046	0.549	0.00739	0.271414
ENSG00000205838	*TTC23L*	0.977	0.00604	0.27771	0.938	0.00744	0.272744
ENSG00000235657	*HLA-A*	-0.595	0.00589	0.27398	-0.556	0.00747	0.272923
ENSG00000108255	*CRYBA1*	0.832	0.00748	0.31537	0.829	0.00748	0.273050
ENSG00000168246	*UBTD2*	-0.583	0.00768	0.31939	-0.581	0.00748	0.273050
ENSG00000164850	*GPER1*	0.775	0.00899	0.34841	0.787	0.00749	0.273170
ENSG00000177679	*SRRM3*	-0.613	0.00786	0.32074	-0.614	0.00751	0.273484
ENSG00000134602	*STK26*	-0.594	0.00706	0.30415	-0.585	0.00751	0.273484
ENSG00000113924	*HGD*	1.612	0.00510	0.24994	1.414	0.00759	0.275242
ENSG00000169299	*PGM2*	-0.568	0.00740	0.31413	-0.561	0.00761	0.275715
ENSG00000157657	*ZNF618*	-0.568	0.00777	0.32074	-0.567	0.00766	0.276491
ENSG00000284967	*FDFT1*	0.546	0.00923	0.35316	0.552	0.00770	0.277380
ENSG00000180628	*PCGF5*	-0.565	0.00798	0.32299	-0.566	0.00774	0.278622
ENSG00000100116	*GCAT*	-0.585	0.00749	0.31537	-0.576	0.00776	0.278971
ENSG00000153936	*HS2ST1*	-0.568	0.00735	0.31268	-0.564	0.00776	0.278998
ENSG00000143110	*C1orf162*	1.830	0.00276	0.17464	1.607	0.00779	0.279532
ENSG00000117519	*CNN3*	-0.562	0.00713	0.30639	-0.554	0.00779	0.279545
ENSG00000180332	*KCTD4*	0.896	0.00721	0.30846	0.879	0.00784	0.279999
ENSG00000170092	*SPDYE5*	0.708	0.00417	0.22396	0.586	0.00788	0.279999
ENSG00000134709	*HOOK1*	-0.587	0.00710	0.30600	-0.575	0.00788	0.279999
ENSG00000268941	*LINC01711*	3.343	0.00788	0.32074	3.340	0.00788	0.279999
ENSG00000284154	*MIR3605*	-3.269	0.00788	0.32074	-3.272	0.00788	0.279999
ENSG00000089022	*MAPKAPK5*	0.554	0.00857	0.33874	0.557	0.00789	0.279999
ENSG00000177042	*TMEM80*	0.627	0.00711	0.30614	0.615	0.00793	0.281357
ENSG00000236778	*INTS6-AS1*	0.853	0.00856	0.33865	0.853	0.00795	0.281878
ENSG00000182240	*BACE2*	-0.610	0.00846	0.33634	-0.603	0.00797	0.281878
ENSG00000197442	*MAP3K5*	-0.696	0.00290	0.17968	-0.606	0.00797	0.281878
ENSG00000260034	*LCMT1-AS2*	0.692	0.00580	0.27131	0.666	0.00800	0.282328

ENSG00000065413	*ANKRD44*	-0.862	0.00182	0.13495	-0.708	0.00806	0.284064
ENSG00000145920	*CPLX2*	1.062	0.00896	0.34775	1.058	0.00815	0.287036
ENSG00000108582	*CPD*	-0.543	0.00867	0.34165	-0.546	0.00818	0.287554
ENSG00000117592	*PRDX6*	-0.561	0.00680	0.29737	-0.547	0.00821	0.287839
ENSG00000087111	*PIGS*	-0.601	0.00502	0.24811	-0.563	0.00824	0.288570
ENSG00000245573	*BDNF-AS*	0.610	0.00657	0.29219	0.591	0.00826	0.288873
ENSG00000113621	*TXNDC15*	-0.558	0.00995	0.36854	-0.569	0.00827	0.289081
ENSG00000128791	*TWSG1*	-0.589	0.00596	0.27577	-0.564	0.00828	0.289158
ENSG00000130309	*COLGALT1*	-0.557	0.00742	0.31465	-0.548	0.00828	0.289158
ENSG00000196586	*MYO6*	-0.541	0.00991	0.36824	-0.551	0.00833	0.290325
ENSG00000205334	*LINC01460*	1.523	0.00661	0.29219	1.442	0.00834	0.290325
ENSG00000131724	*IL13RA1*	-0.584	0.00658	0.29219	-0.564	0.00836	0.290553
ENSG00000104341	*LAPTM4B*	-0.551	0.00755	0.31707	-0.543	0.00839	0.291212
ENSG00000144847	*IGSF11*	-1.590	0.00841	0.33546	-1.593	0.00841	0.291254
ENSG00000188404	*SELL*	-1.032	0.00394	0.21680	-0.912	0.00843	0.291254
ENSG00000182782	*HCAR2*	0.959	0.00672	0.29459	0.886	0.00843	0.291254
ENSG00000180914	*OXTR*	0.717	0.00786	0.32074	0.703	0.00851	0.293116
ENSG00000145365	*TIFA*	-0.575	0.00936	0.35696	-0.584	0.00853	0.293398
ENSG00000204588	*LINC01123*	0.668	0.00346	0.20014	0.570	0.00864	0.295775
ENSG00000064687	*ABCA7*	0.555	0.00822	0.33019	0.549	0.00866	0.296301
ENSG00000162999	*DUSP19*	-0.745	0.00630	0.28585	-0.711	0.00874	0.297834
ENSG00000169599	*NFU1*	-0.646	0.00456	0.23309	-0.572	0.00879	0.298934
ENSG00000185519	*FAM131C*	-0.786	0.00419	0.22398	-0.661	0.00879	0.298934
ENSG00000202354	*RNY3*	-1.293	0.00526	0.25533	-0.758	0.00880	0.298995
ENSG00000141622	*RNF165*	-0.668	0.00768	0.31924	-0.652	0.00888	0.300069
ENSG00000162437	*RAVER2*	-0.553	0.00955	0.36180	-0.555	0.00889	0.300069
ENSG00000262943	*ALOX12P2*	0.571	0.00837	0.33478	0.563	0.00889	0.300069
ENSG00000237333	*MSH5*	0.856	0.00795	0.32260	0.592	0.00889	0.300069
ENSG00000173230	*GOLGB1*	0.564	0.00647	0.28959	0.542	0.00890	0.300069
ENSG00000132965	*ALOX5AP*	6.042	0.00894	0.34775	6.040	0.00894	0.300069
ENSG00000144230	*GPR17*	6.042	0.00894	0.34775	6.040	0.00894	0.300069
ENSG00000120279	*MYCT1*	-6.003	0.00894	0.34775	-6.005	0.00894	0.300069
ENSG00000169302	*STK32A*	6.042	0.00894	0.34775	6.040	0.00894	0.300069
ENSG00000151164	*RAD9B*	1.123	0.00758	0.31707	1.059	0.00896	0.300125
ENSG00000237986	*CELF2-AS2*	1.062	0.00896	0.34775	1.059	0.00896	0.300125
ENSG00000104320	*NBN*	-0.545	0.00913	0.35120	-0.545	0.00897	0.300381
ENSG00000119608	*PROX2*	0.698	0.00987	0.36710	0.702	0.00902	0.301310
ENSG00000112208	*BAG2*	-0.542	0.00964	0.36222	-0.546	0.00905	0.301310
ENSG00000206493	*HLA-E*	-0.711	0.00568	0.26897	-0.555	0.00906	0.301310
ENSG00000151468	*CCDC3*	-0.847	0.00650	0.29025	-0.806	0.00915	0.302964
ENSG00000067082	*KLF6*	-0.540	0.00916	0.35165	-0.540	0.00915	0.302964
ENSG00000151117	*TMEM86A*	-1.398	0.00917	0.35165	-1.401	0.00917	0.302964
ENSG00000137825	*ITPKA*	-0.712	0.00591	0.27485	-0.660	0.00917	0.302964
ENSG00000127366	*TAS2R5*	0.852	0.00743	0.31468	0.825	0.00921	0.303095
ENSG00000117122	*MFAP2*	-0.575	0.00946	0.36054	-0.559	0.00937	0.306681
ENSG00000007944	*MYLIP*	-0.650	0.00909	0.35080	-0.643	0.00942	0.307253
ENSG00000231177	*LINC00852*	0.751	0.00599	0.27617	0.715	0.00959	0.310963
ENSG00000235214	*FAM83C-AS1*	-2.589	0.00961	0.36180	-2.592	0.00961	0.310963
ENSG00000271626	*H3P42*	-2.589	0.00961	0.36180	-2.592	0.00961	0.310963
ENSG00000137674	*MMP20*	2.665	0.00961	0.36180	2.662	0.00961	0.310963
ENSG00000162881	*OXER1*	2.665	0.00961	0.36180	2.662	0.00961	0.310963
ENSG00000120949	*TNFRSF8*	-2.174	0.00423	0.22500	-1.751	0.00963	0.310963
ENSG00000100079	*LGALS2*	-1.747	0.00963	0.36180	-1.751	0.00963	0.310963
ENSG00000260423	*LINC02367*	0.828	0.00241	0.16037	0.660	0.00964	0.311043
ENSG00000177363	*LRRN4CL*	0.972	0.00782	0.32074	0.930	0.00965	0.311043
ENSG00000229589	*ACVR2B-AS1*	0.942	0.00744	0.31473	0.902	0.00982	0.315185

ENSG00000136868	*SLC31A1*	-0.567	0.00788	0.32074	-0.549	0.00983	0.315253
ENSG00000167216	*KATNAL2*	0.744	0.00413	0.22315	0.663	0.00995	0.317511
ENSG00000117152	*RGS4*	-1.747	0.00963	0.36180	-1.532	0.01917	0.458965

b) si*EHMT2*

ENSEMBL ID	Gene Symbol	Bowtie2			STAR		
		logFC	P-Value	FDR	logFC	P-Value	FDR
ENSG00000197915	*HRNR*	4,457	3,91E-32	2,54E-27	4,235	5,27E-46	3,41E-41
ENSG00000144063	*MALL*	-1,946	4,35E-19	7,05E-15	-2,093	2,27E-22	7,35E-18
ENSG00000143631	*FLG*	3,806	2,86E-13	1,54E-09	4,417	7,49E-20	1,05E-15
ENSG00000171903	*CYP4F11*	2,189	7,24E-20	2,35E-15	2,145	1,10E-19	1,05E-15
ENSG00000110492	*MDK*	-1,936	2,19E-19	4,73E-15	-1,948	1,14E-19	1,05E-15
ENSG00000156510	*HKDC1*	2,285	1,20E-17	1,11E-13	2,288	7,15E-19	3,56E-15
ENSG00000204371	*EHMT2*	1,826	5,41E-11	1,30E-07	1,940	1,81E-18	8,38E-15
ENSG00000186480	*INSIG1*	1,884	3,11E-18	4,03E-14	1,868	4,79E-18	2,07E-14
ENSG00000163435	*ELF3*	2,248	4,82E-16	3,91E-12	2,319	9,05E-18	3,54E-14
ENSG00000169174	*PCSK9*	1,882	1,03E-17	1,11E-13	1,876	9,28E-18	3,54E-14
ENSG00000182158	*CREB3L2*	-1,766	4,22E-15	2,49E-11	-1,782	1,94E-15	6,98E-12
ENSG00000168993	*CPLX1*	-1,724	3,75E-12	1,28E-08	-1,780	5,20E-13	1,78E-09
ENSG00000073150	*PANX2*	-1,702	1,49E-12	6,05E-09	-1,718	5,92E-13	1,84E-09
ENSG00000099250	*NRP1*	-1,691	5,78E-13	2,88E-09	-1,670	5,95E-13	1,84E-09
ENSG00000057468	*MSH4*	1,819	8,84E-13	4,09E-09	1,777	7,37E-13	2,17E-09
ENSG00000079459	*FDFT1*	1,480	5,65E-12	1,59E-08	1,495	1,43E-12	4,04E-09
ENSG00000112972	*HMGCS1*	1,461	3,60E-12	1,28E-08	1,459	3,63E-12	9,41E-09
ENSG00000167767	*KRT80*	1,523	4,96E-12	1,53E-08	1,490	1,07E-11	2,66E-08
ENSG00000131069	*ACSS2*	1,477	2,14E-11	5,78E-08	1,484	1,14E-11	2,75E-08
ENSG00000143183	*TMCO1*	-1,414	7,54E-11	1,63E-07	-1,457	1,40E-11	3,25E-08
ENSG00000181458	*TMEM45A*	-1,793	9,28E-11	1,94E-07	-1,792	3,10E-11	6,94E-08
ENSG00000080573	*COL5A3*	-1,937	4,87E-08	5,74E-05	-2,030	3,40E-11	7,35E-08
ENSG00000185262	*UBALD2*	1,403	1,62E-10	3,08E-07	1,447	3,74E-11	7,81E-08
ENSG00000183087	*GAS6*	-1,383	6,24E-10	1,07E-06	-1,470	4,00E-11	8,11E-08
ENSG00000175793	*SFN*	1,397	3,52E-11	8,92E-08	1,387	4,76E-11	9,34E-08
ENSG00000176485	*PLAAT3*	-1,911	6,30E-11	1,46E-07	-1,832	5,09E-11	9,71E-08
ENSG00000159055	*MIS18A*	1,439	1,25E-10	2,45E-07	1,458	5,70E-11	1,06E-07
ENSG00000164484	*TMEM200A*	-1,669	6,99E-11	1,56E-07	-1,664	6,74E-11	1,21E-07
ENSG00000245532	*NEAT1*	1,344	1,12E-10	2,28E-07	1,339	1,31E-10	2,30E-07
ENSG00000186529	*CYP4F3*	1,658	1,49E-09	2,35E-06	1,669	3,69E-10	6,30E-07
ENSG00000100292	*HMOX1*	1,392	6,15E-10	1,07E-06	1,390	3,96E-10	6,59E-07
ENSG00000272398	*CD24*	-1,638	3,62E-07	0,000355	-1,796	9,40E-10	1,49E-06
ENSG00000135318	*NT5E*	-1,364	5,19E-10	9,34E-07	-1,331	1,11E-09	1,71E-06
ENSG00000146648	*EGFR*	-1,273	3,10E-09	4,79E-06	-1,277	2,25E-09	3,35E-06
ENSG00000120708	*TGFBI*	7,981	3,99E-09	5,89E-06	8,024	2,27E-09	3,35E-06
ENSG00000135074	*ADAM19*	-1,756	4,42E-09	6,37E-06	-1,721	2,70E-09	3,89E-06
ENSG00000160285	*LSS*	1,282	6,77E-09	9,34E-06	1,261	3,37E-09	4,61E-06
ENSG00000167549	*CORO6*	1,421	1,12E-09	1,86E-06	1,366	3,42E-09	4,61E-06
ENSG00000052802	*MSMO1*	1,239	5,69E-09	8,03E-06	1,228	7,26E-09	9,22E-06
ENSG00000237686	*LOC101929705*	1,626	3,83E-08	4,60E-05	1,668	1,49E-08	1,83E-05
ENSG00000117407	*ARTN*	1,224	2,13E-08	2,66E-05	1,220	2,23E-08	2,67E-05
ENSG00000234794	*HLA-DRA*	-2,683	0,00243	0,256608	-2,303	2,40E-08	2,68E-05
ENSG00000078018	*MAP2*	1,243	1,63E-08	2,16E-05	1,214	2,74E-08	3,01E-05
ENSG00000174938	*SEZ6L2*	-1,176	8,91E-08	0,000101	-1,205	3,25E-08	3,51E-05
ENSG00000137440	*FGFBP1*	1,284	2,13E-08	2,66E-05	1,253	3,73E-08	3,96E-05
ENSG00000113161	*HMGCR*	1,125	7,15E-08	8,28E-05	1,137	4,98E-08	5,21E-05
ENSG00000179750	*APOBEC3B*	1,375	3,82E-08	4,60E-05	1,275	7,13E-08	7,22E-05

ENSG00000153253	*SCN3A*	-1,661	9,62E-08	0,000106	-1,623	8,31E-08	8,29E-05
ENSG00000120437	*ACAT2*	1,139	2,55E-07	0,000272	1,164	8,62E-08	8,46E-05
ENSG00000162496	*DHRS3*	1,305	2,63E-06	0,001874	1,464	1,02E-07	9,86E-05
ENSG00000188015	*S100A3*	1,978	9,53E-08	0,000106	1,881	1,09E-07	0,00010
ENSG00000187867	*PALM3*	-1,256	4,99E-07	0,000455	-1,279	2,13E-07	0,00020
ENSG00000128564	*VGF*	-1,193	3,13E-07	0,000326	-1,203	2,39E-07	0,00022
ENSG00000111799	*COL12A1*	-1,135	2,17E-07	0,000234	-1,113	2,58E-07	0,00024
ENSG00000184602	*SNN*	-1,195	4,04E-07	0,000385	-1,209	2,67E-07	0,00024
ENSG00000110917	*MLEC*	-1,068	3,16E-07	0,000326	-1,066	3,27E-07	0,00029
ENSG00000165802	*NSMF*	-1,021	1,01E-06	0,000850	-1,064	3,31E-07	0,00029
ENSG00000023445	*BIRC3*	1,273	3,50E-07	0,000355	1,273	3,41E-07	0,00029
ENSG00000125462	*C1orf61*	1,095	4,40E-07	0,000413	1,100	3,81E-07	0,00033
ENSG00000145284	*SCD5*	-1,223	3,69E-07	0,000357	-1,203	4,70E-07	0,00040
ENSG00000212724	*KRTAP2-3*	-1,215	0,00039	0,080164	-1,372	4,89E-07	0,00040
ENSG00000177542	*SLC25A22*	-1,068	1,09E-06	0,000871	-1,089	5,61E-07	0,00046
ENSG00000242265	*PEG10*	-1,304	6,25E-07	0,000563	-1,299	6,31E-07	0,00050
ENSG00000084234	*APLP2*	-1,034	6,84E-07	0,000607	-1,035	6,56E-07	0,00052
ENSG00000112715	*VEGFA*	1,044	9,68E-07	0,000826	1,050	7,96E-07	0,00062
ENSG00000164483	*SAMD3*	-1,964	1,02E-06	0,000850	-1,828	8,16E-07	0,00063
ENSG00000157087	*ATP2B2*	-1,886	3,56E-07	0,000355	-1,778	8,29E-07	0,00063
ENSG00000229261	*LOC101928994*	1,336	1,98E-06	0,001478	1,360	9,03E-07	0,00068
ENSG00000075426	*FOSL2*	1,027	1,32E-06	0,001038	1,041	9,19E-07	0,00069
ENSG00000121073	*SLC35B1*	-0,989	3,92E-06	0,002594	-1,041	9,85E-07	0,00073
ENSG00000082146	*STRADB*	-1,069	5,37E-05	0,020374	-1,224	1,00E-06	0,00073
ENSG00000155629	*PIK3AP1*	-1,424	4,47E-07	0,000414	-1,303	1,12E-06	0,00081
ENSG00000162745	*OLFML2B*	-1,980	5,78E-06	0,003500	-1,997	1,13E-06	0,00081
ENSG00000150961	*SEC24D*	-0,995	3,71E-06	0,002480	-1,037	1,16E-06	0,00082
ENSG00000102098	*SCML2*	1,016	5,62E-06	0,003435	1,070	1,37E-06	0,00095
ENSG00000116983	*HPCAL4*	-1,216	2,63E-06	0,001874	-1,246	1,38E-06	0,00095
ENSG00000104442	*ARMC1*	1,036	1,33E-06	0,001038	1,019	1,81E-06	0,00121
ENSG00000148248	*SURF4*	-0,957	6,78E-06	0,003893	-0,997	1,90E-06	0,00123
ENSG00000111319	*SCNN1A*	1,134	7,70E-07	0,000675	1,080	1,94E-06	0,00125
ENSG00000111145	*ELK3*	-1,039	1,67E-06	0,001257	-1,029	1,99E-06	0,00126
ENSG00000050405	*LIMA1*	1,056	1,08E-06	0,000871	1,019	2,14E-06	0,00135
ENSG00000138759	*FRAS1*	-0,993	2,89E-06	0,001990	-0,999	2,24E-06	0,00140
ENSG00000187475	*H1-6*	-2,117	2,28E-06	0,001660	-2,112	2,28E-06	0,00141
ENSG00000213937	*CLDN9*	7,289	4,54E-06	0,002902	7,358	2,48E-06	0,00151
ENSG00000225855	*RUSC1-AS1*	1,109	2,66E-06	0,001874	1,114	2,50E-06	0,00151
ENSG00000074416	*MGLL*	1,538	9,40E-06	0,005208	1,617	2,54E-06	0,00153
ENSG00000139318	*DUSP6*	-1,013	3,69E-06	0,002480	-1,025	2,78E-06	0,00165
ENSG00000158104	*HPD*	1,334	0,00015	0,040846	1,580	3,29E-06	0,00190
ENSG00000160014	*CALM3*	0,982	2,80E-06	0,001953	0,972	3,33E-06	0,00191
ENSG00000119335	*SET*	0,861	3,24E-05	0,013726	0,959	3,57E-06	0,00203
ENSG00000117322	*CR2*	-1,354	2,04E-05	0,009400	-1,406	3,88E-06	0,00213
ENSG00000168243	*GNG4*	-1,023	0,00116	0,166078	-1,205	4,05E-06	0,00221
ENSG00000101935	*AMMECR1*	1,001	5,61E-06	0,003435	1,008	4,57E-06	0,00245
ENSG00000157570	*TSPAN18*	-1,050	0,00011	0,032629	-1,204	4,67E-06	0,00248
ENSG00000116133	*DHCR24*	0,954	4,61E-06	0,002905	0,949	5,04E-06	0,00266
ENSG00000006016	*CRLF1*	-1,079	1,89E-05	0,008898	-1,128	5,34E-06	0,00279
ENSG00000179593	*ALOX15B*	-3,191	8,97E-07	0,000776	-2,705	5,57E-06	0,00289
ENSG00000073849	*ST6GAL1*	-0,981	1,63E-05	0,007851	-1,025	5,92E-06	0,00304
ENSG00000008735	*MAPK8IP2*	-0,998	1,21E-05	0,006217	-1,024	6,14E-06	0,00307
ENSG00000174460	*ZCCHC12*	-2,145	4,28E-05	0,017114	-2,319	6,22E-06	0,00307
ENSG00000124334	*IL9R*	3,941	8,08E-05	0,026882	2,622	6,24E-06	0,00307
ENSG00000135480	*KRT7*	1,420	6,52E-06	0,003776	1,353	6,45E-06	0,00314
ENSG00000235269	*LINC02331*	-1,100	6,22E-06	0,003700	-1,074	6,93E-06	0,00335

ENSG00000101188	*NTSR1*	-0,969	6,33E-06	0,003700	-0,960	7,14E-06	0,00343
ENSG00000156042	*CFAP70*	0,980	1,02E-05	0,005528	0,988	7,64E-06	0,00362
ENSG00000188897	*LOC400499*	0,981	1,03E-05	0,005534	0,985	7,73E-06	0,00363
ENSG00000162426	*SLC45A1*	-1,233	1,43E-05	0,007029	-1,229	8,68E-06	0,00402
ENSG00000184613	*NELL2*	-1,777	0,00011	0,033272	-1,915	8,76E-06	0,00403
ENSG00000145246	*ATP10D*	-1,019	1,98E-05	0,009256	-1,043	8,95E-06	0,00409
ENSG00000134531	*EMP1*	1,127	6,32E-06	0,003700	1,101	9,63E-06	0,00428
ENSG00000198911	*SREBF2*	0,914	1,22E-05	0,006217	0,923	9,64E-06	0,00428
ENSG00000180758	*GPR157*	-1,109	4,21E-06	0,002760	-1,058	9,81E-06	0,00433
ENSG00000164236	*ANKRD33B*	1,059	7,05E-06	0,004008	1,036	1,02E-05	0,00448
ENSG00000160752	*FDPS*	0,924	1,71E-05	0,008107	0,931	1,05E-05	0,00456
ENSG00000147883	*CDKN2B*	0,977	4,35E-06	0,002821	0,934	1,09E-05	0,00470
ENSG00000165424	*ZCCHC24*	-1,191	1,01E-05	0,005509	-1,180	1,12E-05	0,00477
ENSG00000147124	*ZNF41*	-0,980	1,30E-05	0,006487	-0,983	1,15E-05	0,00489
ENSG00000230667	*SETSIP*	1,070	1,42E-06	0,001095	0,939	1,18E-05	0,00496
ENSG00000137414	*FAM8A1*	-0,978	1,10E-05	0,005814	-0,971	1,19E-05	0,00496
ENSG00000163820	*FYCO1*	-0,926	1,65E-05	0,007876	-0,935	1,23E-05	0,00510
ENSG00000189143	*CLDN4*	0,926	1,25E-05	0,006280	0,921	1,35E-05	0,00554
ENSG00000136490	*LIMD2*	-1,083	5,85E-05	0,021300	-1,147	1,39E-05	0,00566
ENSG00000238741	*SCARNA7*	-0,946	1,35E-05	0,006697	-0,943	1,41E-05	0,00566
ENSG00000137491	*SLCO2B1*	1,627	5,53E-06	0,003435	1,491	1,45E-05	0,00580
ENSG00000185515	*BRCC3*	0,935	1,63E-05	0,007851	0,930	1,51E-05	0,00597
ENSG00000133687	*TMTC1*	-1,271	9,99E-06	0,005489	-1,220	1,58E-05	0,00615
ENSG00000198794	*SCAMP5*	-1,119	1,19E-05	0,006167	-1,080	1,63E-05	0,00630
ENSG00000150201	*FXYD4*	-1,450	0,00134	0,176284	-1,620	1,70E-05	0,00650
ENSG00000186063	*AIDA*	-0,853	9,92E-05	0,031531	-0,906	2,03E-05	0,00769
ENSG00000128228	*SDF2L1*	-0,894	5,34E-05	0,020374	-0,932	2,26E-05	0,00853
ENSG00000167508	*MVD*	0,928	1,11E-05	0,005814	0,888	2,44E-05	0,00915
ENSG00000166128	*RAB8B*	-0,950	2,84E-05	0,012183	-0,948	2,49E-05	0,00928
ENSG00000179388	*EGR3*	-1,290	2,55E-05	0,011002	-1,285	2,50E-05	0,00928
ENSG00000026751	*SLAMF7*	-1,734	4,69E-05	0,018640	-1,759	2,62E-05	0,00960
ENSG00000137834	*SMAD6*	1,314	1,24E-05	0,006280	1,275	2,66E-05	0,00968
ENSG00000143845	*ETNK2*	-0,990	7,01E-05	0,024703	-1,023	2,78E-05	0,00994
ENSG00000067064	*IDI1*	0,889	2,53E-05	0,011002	0,882	2,79E-05	0,00994
ENSG00000120742	*SERP1*	-0,905	1,56E-05	0,007594	-0,875	2,89E-05	0,01010
ENSG00000126709	*IFI6*	-1,292	0,00010	0,031531	-1,320	2,90E-05	0,01010
ENSG00000144597	*EAF1*	0,920	2,24E-05	0,010028	0,902	3,03E-05	0,01050
ENSG00000158716	*DUSP23*	-2,259	7,46E-05	0,025583	-2,340	3,27E-05	0,01128
ENSG00000215114	*UBXN2B*	0,870	5,83E-05	0,021300	0,893	3,32E-05	0,01139
ENSG00000151224	*MAT1A*	-1,258	5,63E-05	0,020970	-1,239	3,43E-05	0,01171
ENSG00000197457	*STMN3*	-0,908	5,06E-05	0,019757	-0,914	3,49E-05	0,01185
ENSG00000134013	*LOXL2*	-1,057	2,22E-05	0,010021	-0,998	3,61E-05	0,01218
ENSG00000188536	*HBA2*	-1,756	0,00185	0,220208	-1,625	3,77E-05	0,01268
ENSG00000106853	*PTGR1*	0,938	2,34E-05	0,010308	0,894	3,83E-05	0,01279
ENSG00000079101	*CLUL1*	-2,209	0,00049	0,093909	-2,631	4,05E-05	0,01332
ENSG00000100906	*NFKBIA*	0,895	3,32E-05	0,013871	0,877	4,18E-05	0,01369
ENSG00000150625	*GPM6A*	-2,384	2,16E-05	0,009877	-2,141	4,28E-05	0,01393
ENSG00000132837	*DMGDH*	-1,104	0,00012	0,034410	-1,128	4,35E-05	0,01411
ENSG00000206450	*HLA-B*	-0,854	0,00051	0,096566	-0,880	4,43E-05	0,01428
ENSG00000162998	*FRZB*	1,364	0,00018	0,048044	1,478	4,57E-05	0,01468
ENSG00000245954	*LINC02273*	-1,784	3,34E-05	0,013871	-1,730	4,69E-05	0,01497
ENSG00000171421	*MRPL36*	-0,912	6,60E-05	0,023521	-0,917	4,88E-05	0,01543
ENSG00000230798	*FOXD3-AS1*	-2,160	0,00035	0,075198	-2,298	4,94E-05	0,01548
ENSG00000206172	*HBA1*	-1,543	0,00779	0,505555	-1,547	4,94E-05	0,01548
ENSG00000143341	*HMCN1*	-1,071	0,00094	0,145823	-1,252	5,11E-05	0,01588
ENSG00000114115	*RBP1*	-0,850	6,93E-05	0,024549	-0,861	5,12E-05	0,01588

ENSG00000163297	*ANTXR2*	-0,914	7,28E-05	0,025375	-0,922	5,26E-05	0,01620
ENSG00000140280	*LYSMD2*	-1,044	5,77E-05	0,021265	-1,045	5,35E-05	0,01635
ENSG00000272636	*DOC2B*	-1,106	4,02E-05	0,016385	-0,952	5,56E-05	0,01693
ENSG00000135437	*RDH5*	1,843	7,98E-05	0,026824	1,876	5,69E-05	0,01717
ENSG00000169783	*LINGO1*	-0,955	5,19E-05	0,020142	-0,948	5,72E-05	0,01717
ENSG00000137193	*PIM1*	0,969	5,61E-05	0,020970	0,953	5,78E-05	0,01726
ENSG00000109501	*WFS1*	-0,847	0,00013	0,037316	-0,884	5,98E-05	0,01771
ENSG00000075420	*FNDC3B*	-0,882	3,31E-05	0,013871	-0,846	6,24E-05	0,01838
ENSG00000174010	*KLHL15*	0,877	5,74E-05	0,021265	0,873	6,32E-05	0,01853
ENSG00000130701	*RBBP8NL*	2,433	0,00118	0,166302	2,550	6,40E-05	0,01870
ENSG00000132603	*NIP7*	0,842	6,55E-05	0,023474	0,840	6,52E-05	0,01897
ENSG00000158286	*RNF207*	0,866	6,34E-05	0,022952	0,861	6,67E-05	0,01925
ENSG00000123989	*CHPF*	-0,826	8,32E-05	0,027375	-0,836	6,68E-05	0,01925
ENSG00000018236	*CNTN1*	-1,010	0,00304	0,289724	-1,241	6,72E-05	0,01927
ENSG00000164749	*HNF4G*	-1,071	7,85E-05	0,026800	-1,050	6,82E-05	0,01948
ENSG00000119328	*ABITRAM*	-0,812	0,00024	0,057958	-0,875	6,97E-05	0,01979
ENSG00000120215	*MLANA*	1,183	0,00010	0,031531	1,193	6,99E-05	0,01979
ENSG00000124733	*MEA1*	-0,829	0,00014	0,040312	-0,857	7,28E-05	0,02036
ENSG00000103855	*CD276*	-0,826	0,00011	0,032629	-0,839	7,46E-05	0,02072
ENSG00000170417	*TMEM182*	-1,035	5,35E-05	0,020374	-0,996	7,61E-05	0,02099
ENSG00000168785	*TSPAN5*	-0,842	7,91E-05	0,026824	-0,841	7,64E-05	0,02099
ENSG00000005893	*LAMP2*	-0,878	5,35E-05	0,020374	-0,850	7,88E-05	0,02157
ENSG00000105329	*TGFB1*	-0,850	0,00021	0,053776	-0,891	8,06E-05	0,02195
ENSG00000142192	*APP*	-0,795	0,00013	0,037556	-0,816	8,36E-05	0,02267
ENSG00000143061	*IGSF3*	-0,838	0,00011	0,033122	-0,837	9,63E-05	0,02590
ENSG00000224715	*LOC339685*	1,079	0,00018	0,047246	1,114	9,78E-05	0,02603
ENSG00000163132	*MSX1*	0,774	0,00022	0,054934	0,814	9,83E-05	0,02603
ENSG00000130402	*ACTN4*	0,811	9,64E-05	0,030994	0,804	9,84E-05	0,02603
ENSG00000187730	*GABRD*	-1,284	4,11E-05	0,016544	-1,161	0,000101	0,02640
ENSG00000125744	*RTN2*	-0,914	0,00019	0,051343	-0,941	0,000102	0,02640
ENSG00000126231	*PROZ*	1,348	0,00010	0,031531	1,352	0,000102	0,02640
ENSG00000101955	*SRPX*	-1,289	7,32E-06	0,004125	-1,056	0,000103	0,02651
ENSG00000151090	*THRB*	-1,286	0,00020	0,051908	-1,334	0,000105	0,02685
ENSG00000109171	*SLAIN2*	-0,787	0,00023	0,057399	-0,825	0,000109	0,02793
ENSG00000262074	*SNORD3B-2*	-0,801	0,00010	0,031541	-0,795	0,000110	0,02795
ENSG00000265185	*SNORD3B-1*	-0,800	0,00010	0,031531	-0,795	0,000111	0,02795
ENSG00000100344	*PNPLA3*	0,907	0,00036	0,076344	0,957	0,000111	0,02795
ENSG00000189320	*FAM180A*	-1,618	0,00042	0,084312	-1,745	0,000114	0,02861
ENSG00000100167	*SEPTIN3*	-0,934	0,00045	0,087558	-1,016	0,000116	0,02892
ENSG00000134042	*MRO*	-1,380	0,00020	0,051908	-1,387	0,000117	0,02892
ENSG00000129910	*CDH15*	-1,383	0,00011	0,033272	-1,294	0,000118	0,02906
ENSG00000153904	*DDAH1*	0,850	8,08E-05	0,026882	0,827	0,000121	0,02947
ENSG00000165704	*HPRT1*	-0,791	0,00032	0,070037	-0,827	0,000121	0,02947
ENSG00000197580	*BCO2*	1,071	8,19E-05	0,027076	1,024	0,000122	0,02973
ENSG00000169247	*SH3TC2*	-0,865	0,00015	0,041711	-0,868	0,000128	0,03099
ENSG00000173530	*TNFRSF10D*	0,842	9,66E-05	0,030994	0,820	0,000129	0,03099
ENSG00000130522	*JUND*	0,784	0,00019	0,050228	0,800	0,000136	0,03258
ENSG00000166961	*MS4A15*	-1,258	7,08E-05	0,024825	-1,179	0,000140	0,03359
ENSG00000174099	*MSRB3*	-0,860	0,00014	0,039953	-0,848	0,000149	0,03521
ENSG00000130303	*BST2*	-1,898	0,00061	0,109779	-1,876	0,000153	0,03604
ENSG00000118596	*SLC16A7*	-0,812	0,00025	0,060025	-0,836	0,000155	0,03624
ENSG00000213906	*LTB4R2*	1,251	5,00E-05	0,019632	0,985	0,000160	0,03698
ENSG00000197635	*DPP4*	-1,099	0,00153	0,195030	-1,199	0,000162	0,03722
ENSG00000206435	*HLA-C*	-0,875	0,00020	0,051818	-0,803	0,000169	0,03835
ENSG00000089472	*HEPH*	-1,356	0,00024	0,058927	-1,260	0,000172	0,03907
ENSG00000132429	*POPDC3*	-0,947	0,00015	0,041554	-0,916	0,000175	0,03937

ENSG00000129990	*SYT5*	-1,576	0,00015	0,042127	-1,418	0,000177	0,03981
ENSG00000126016	*AMOT*	-1,001	0,00024	0,058927	-1,010	0,000183	0,04089
ENSG00000132964	*CDK8*	-0,841	0,00013	0,037383	-0,810	0,000184	0,04089
ENSG00000120306	*CYSTM1*	-0,829	0,00028	0,065526	-0,845	0,000186	0,04127
ENSG00000105605	*CACNG7*	-2,581	6,48E-05	0,023330	-2,320	0,000201	0,04429
ENSG00000164171	*ITGA2*	-0,777	0,00023	0,057248	-0,781	0,000202	0,04449
ENSG00000151468	*CCDC3*	-1,183	0,00014	0,039953	-1,146	0,000206	0,04509
ENSG00000253276	*CCDC71L*	-0,822	0,00020	0,051908	-0,819	0,000212	0,04602
ENSG00000126581	*BECN1*	0,714	0,00099	0,150443	0,795	0,000213	0,04613
ENSG00000242498	*ARPIN*	-0,848	0,00023	0,057074	-0,850	0,000221	0,04751
ENSG00000135002	*RFK*	-0,807	0,00022	0,056367	-0,805	0,000222	0,04764
ENSG00000111412	*C12orf49*	0,805	0,00016	0,043916	0,785	0,000225	0,04816
ENSG00000237975	*FLG-AS1*	2,225	0,00125	0,169289	2,421	0,000231	0,04908
ENSG00000163536	*SERPINI1*	-0,986	0,00046	0,089554	-1,001	0,000232	0,04908
ENSG00000123104	*ITPR2*	-0,827	0,00044	0,086556	-0,844	0,000232	0,04908
ENSG00000172986	*GXYLT2*	-1,075	0,00041	0,082975	-1,097	0,000233	0,04914
ENSG00000197093	*GAL3ST4*	1,313	0,00046	0,089384	1,369	0,000233	0,04914
ENSG00000118680	*MYL12B*	0,774	0,00021	0,054934	0,763	0,000249	0,05227
ENSG00000160460	*SPTBN4*	-0,946	0,00028	0,065526	-0,928	0,000251	0,05256
ENSG00000106025	*TSPAN12*	-0,835	0,00061	0,109779	-0,879	0,000253	0,05256
ENSG00000182253	*SYNM*	1,423	5,55E-05	0,020921	1,282	0,000258	0,05316
ENSG00000250673	*REELD1*	1,255	0,00038	0,079125	1,282	0,000258	0,05316
ENSG00000176641	*RNF152*	-1,425	0,00056	0,104891	-1,508	0,000260	0,05335
ENSG00000100097	*LGALS1*	-0,744	0,00037	0,077129	-0,759	0,000261	0,05335
ENSG00000128872	*TMOD2*	-0,847	0,00025	0,060633	-0,842	0,000264	0,05369
ENSG00000167972	*ABCA3*	-0,898	0,00080	0,130387	-0,947	0,000266	0,05396
ENSG00000100146	*SOX10*	1,521	0,00035	0,075216	1,548	0,000268	0,05411
ENSG00000107984	*DKK1*	-0,749	0,00028	0,065526	-0,750	0,000272	0,05479
ENSG00000198431	*TXNRD1*	0,746	0,00029	0,067565	0,749	0,000274	0,05501
ENSG00000109107	*ALDOC*	0,803	0,00093	0,144999	0,844	0,000276	0,05532
ENSG00000198886	*ND4*	0,748	0,00027	0,064093	0,747	0,000279	0,05566
ENSG00000065183	*WDR3*	0,748	0,00033	0,073642	0,755	0,000281	0,05594
ENSG00000104967	*NOVA2*	-1,377	0,00029	0,067565	-1,368	0,000284	0,05598
ENSG00000120738	*EGR1*	-0,748	0,00034	0,073642	-0,757	0,000284	0,05598
ENSG00000161921	*CXCL16*	-0,877	0,00015	0,041486	-0,826	0,000286	0,05598
ENSG00000151348	*EXT2*	-0,741	0,00046	0,089279	-0,764	0,000289	0,05629
ENSG00000197859	*ADAMTSL2*	-1,640	0,00043	0,085353	-1,590	0,000289	0,05629
ENSG00000077274	*CAPN6*	-1,015	0,00031	0,069848	-1,006	0,000291	0,05658
ENSG00000187123	*LYPD6*	-1,018	0,00019	0,050803	-0,982	0,000304	0,05882
ENSG00000164406	*LEAP2*	0,932	0,00055	0,102985	0,971	0,000305	0,05882
ENSG00000066117	*SMARCD1*	-0,747	0,00039	0,080763	-0,756	0,000317	0,06096
ENSG00000055163	*CYFIP2*	-0,921	0,00013	0,037383	-0,844	0,000325	0,06208
ENSG00000005486	*RHBDD2*	-0,773	0,00034	0,074105	-0,772	0,000330	0,06302
ENSG00000185650	*ZFP36L1*	-0,743	0,00033	0,072994	-0,742	0,000336	0,06384
ENSG00000198208	*RPS6KL1*	0,864	0,00022	0,056161	0,836	0,000338	0,06416
ENSG00000160161	*CILP2*	-0,797	0,00034	0,074105	-0,792	0,000344	0,06466
ENSG00000178297	*TMPRSS9*	0,965	0,00055	0,102985	0,999	0,000344	0,06466
ENSG00000100564	*PIGH*	-0,872	0,00034	0,073642	-0,855	0,000345	0,06466
ENSG00000165804	*ZNF219*	-0,838	0,00043	0,085484	-0,850	0,000346	0,06468
ENSG00000170396	*ZNF804A*	-6,310	0,00242	0,256608	-6,718	0,000352	0,06512
ENSG00000156587	*UBE2L6*	-0,907	0,00141	0,183883	-0,993	0,000353	0,06512
ENSG00000001084	*GCLC*	0,752	0,00030	0,068318	0,741	0,000358	0,06598
ENSG00000157851	*DPYSL5*	-0,864	0,00028	0,065526	-0,832	0,000361	0,06634
ENSG00000196358	*NTNG2*	-1,303	0,00059	0,108634	-1,345	0,000363	0,06649
ENSG00000198909	*MAP3K3*	-0,793	0,00031	0,069192	-0,775	0,000372	0,06772
ENSG00000109787	*KLF3*	0,761	0,00044	0,086713	0,769	0,000376	0,06825

ENSG00000002745	*WNT16*	-0,755	0,00037	0,077692	-0,753	0,000379	0,06858
ENSG00000254911	*SCARNA9*	-0,955	0,00062	0,110318	-0,946	0,000385	0,06955
ENSG00000172819	*RARG*	0,793	0,00041	0,082975	0,788	0,000388	0,06983
ENSG00000237452	*BHMG1*	-0,839	0,00020	0,051818	-0,788	0,000402	0,07222
ENSG00000163884	*KLF15*	-2,312	9,83E-05	0,031388	-1,998	0,000404	0,07239
ENSG00000176723	*ZNF843*	-1,214	0,00040	0,082975	-1,180	0,000407	0,07262
ENSG00000002586	*CD99*	-0,864	0,00127	0,171908	-0,815	0,000408	0,07266
ENSG00000161677	*JOSD2*	-0,841	0,00037	0,078114	-0,813	0,000421	0,07443
ENSG00000186104	*CYP2R1*	-0,759	0,00078	0,129726	-0,789	0,000429	0,07465
ENSG00000112175	*BMP5*	1,618	0,00165	0,206277	1,760	0,000429	0,07465
ENSG00000227110	*LMCD1-AS1*	-1,364	0,00932	0,549077	-1,767	0,000429	0,07465
ENSG00000122642	*FKBP9*	-0,758	0,00041	0,083771	-0,746	0,000433	0,07478
ENSG00000134762	*DSC3*	-0,830	0,00195	0,226021	-0,917	0,000434	0,07490
ENSG00000139112	*GABARAPL1*	-0,743	0,00097	0,148826	-0,785	0,000441	0,07537
ENSG00000038427	*VCAN*	-0,719	0,00048	0,092164	-0,723	0,000442	0,07537
ENSG00000006025	*OSBPL7*	0,879	0,00022	0,055468	0,822	0,000442	0,07537
ENSG00000069020	*MAST4*	-1,151	0,00342	0,310474	-1,319	0,000443	0,07537
ENSG00000214402	*LCNL1*	3,708	0,00045	0,087750	3,712	0,000448	0,07564
ENSG00000172478	*MAB21L4*	3,621	0,00079	0,130387	3,712	0,000448	0,07564
ENSG00000196611	*MMP1*	-3,526	0,00444	0,367039	-3,965	0,000448	0,07564
ENSG00000074047	*GLI2*	-0,976	0,00014	0,039953	-0,874	0,000460	0,07748
ENSG00000189334	*S100A14*	0,750	0,00061	0,109779	0,759	0,000462	0,07754
ENSG00000146070	*PLA2G7*	-1,296	0,00338	0,308675	-1,322	0,000464	0,07754
ENSG00000110921	*MVK*	0,794	0,00027	0,064093	0,757	0,000465	0,07754
ENSG00000197774	*EME2*	0,786	0,00029	0,067095	0,759	0,000465	0,07754
ENSG00000163624	*CDS1*	0,792	0,00058	0,107104	0,796	0,000469	0,07772
ENSG00000145244	*CORIN*	0,782	0,00085	0,135366	0,803	0,000480	0,07939
ENSG00000204022	*LIPJ*	-2,148	0,00076	0,128487	-2,205	0,000488	0,08032
ENSG00000163344	*PMVK*	-0,862	0,00029	0,066378	-0,806	0,000499	0,08171
ENSG00000263934	*SNORD3A*	-0,660	0,00131	0,174181	-0,715	0,000501	0,08175
ENSG00000264940	*SNORD3C*	-0,666	0,00119	0,166302	-0,715	0,000502	0,08179
ENSG00000109929	*SC5D*	0,731	0,00062	0,111600	0,741	0,000521	0,08448
ENSG00000256980	*KHDC1L*	1,286	0,00031	0,069094	1,203	0,000521	0,08448
ENSG00000183255	*PTTG1IP*	-0,720	0,00058	0,107104	-0,725	0,000525	0,08494
ENSG00000153234	*NR4A2*	-0,915	0,00023	0,056383	-0,847	0,000532	0,08587
ENSG00000156298	*TSPAN7*	-1,301	0,00056	0,104958	-1,294	0,000535	0,08612
ENSG00000125534	*PPDPF*	-0,735	0,00054	0,102172	-0,731	0,000543	0,08689
ENSG00000141458	*NPC1*	0,722	0,00063	0,112899	0,728	0,000543	0,08689
ENSG00000198673	*TAFA2*	-0,917	0,00112	0,162212	-0,969	0,000546	0,08710
ENSG00000185669	*SNAI3*	1,178	0,00083	0,134022	1,215	0,000549	0,08724
ENSG00000136888	*ATP6V1G1*	0,733	0,00055	0,103635	0,729	0,000565	0,08940
ENSG00000196754	*S100A2*	0,844	0,00094	0,145823	0,870	0,000566	0,08940
ENSG00000221676	*RNU6ATAC*	-1,405	0,00016	0,043998	-1,042	0,000567	0,08940
ENSG00000059728	*MXD1*	0,853	0,00035	0,075198	0,814	0,000568	0,08940
ENSG00000092096	*SLC22A17*	-0,855	0,00264	0,268756	-0,957	0,000569	0,08940
ENSG00000177383	*MAGEF1*	0,748	0,00064	0,114096	0,754	0,000585	0,09134
ENSG00000139445	*FOXN4*	-1,665	0,00082	0,133354	-1,600	0,000591	0,09216
ENSG00000163513	*TGFBR2*	-0,753	0,00066	0,117380	-0,754	0,000595	0,09253
ENSG00000162909	*CAPN2*	-0,692	0,00085	0,135366	-0,710	0,000602	0,09315
ENSG00000198763	*ND2*	0,726	0,00041	0,083771	0,704	0,000604	0,09315
ENSG00000089327	*FXYD5*	-0,830	0,00042	0,084164	-0,787	0,000605	0,09315
ENSG00000020181	*ADGRA2*	-0,824	0,00091	0,143633	-0,833	0,000606	0,09315
ENSG00000100116	*GCAT*	-0,763	0,00050	0,095040	-0,743	0,000608	0,09322
ENSG00000155660	*PDIA4*	-0,731	0,00041	0,082975	-0,707	0,000619	0,09446
ENSG00000221890	*NPTXR*	-0,829	0,00050	0,095722	-0,811	0,000631	0,09607
ENSG00000183250	*LINC01547*	-1,004	0,00077	0,129102	-0,995	0,000641	0,09726

ENSG00000197444	*OGDHL*	-0,935	0,00081	0,131620	-0,904	0,000643	0,09737
ENSG00000170464	*DNAJC18*	-0,921	0,00059	0,107893	-0,890	0,000650	0,09829
ENSG00000139508	*SLC46A3*	-0,935	0,00821	0,523212	-1,160	0,000662	0,09986
ENSG00000280997	*CCAT2*	-6,596	0,00067	0,117489	-6,594	0,000667	0,10031
ENSG00000138685	*FGF2*	-0,778	0,00069	0,121106	-0,776	0,000684	0,10240
ENSG00000183696	*UPP1*	0,712	0,00071	0,121539	0,710	0,000700	0,10457
ENSG00000109472	*CPE*	-0,664	0,00252	0,262271	-0,736	0,000705	0,10507
ENSG00000158406	*H4C8*	-0,702	0,00087	0,137564	-0,713	0,000715	0,10630
ENSG00000198695	*ND6*	0,716	0,00051	0,096310	0,696	0,000717	0,10635
ENSG00000184076	*UQCR10*	-0,731	0,00094	0,145823	-0,738	0,000737	0,10843
ENSG00000099194	*SCD*	0,697	0,00070	0,121493	0,692	0,000763	0,11160
ENSG00000135048	*CEMIP2*	-0,702	0,00211	0,236599	-0,759	0,000781	0,11373
ENSG00000177494	*ZBED2*	-0,746	0,00067	0,117489	-0,737	0,000790	0,11449
ENSG00000067715	*SYT1*	-0,742	0,00156	0,196920	-0,781	0,000792	0,11449
ENSG00000171658	*NMRAL2P*	3,621	0,00079	0,130387	3,625	0,000795	0,11449
ENSG00000165801	*ARHGEF40*	-0,905	0,00093	0,144999	-0,889	0,000803	0,11504
ENSG00000139636	*LMBR1L*	-0,706	0,00218	0,242382	-0,766	0,000804	0,11504
ENSG00000162551	*ALPL*	-2,084	0,00182	0,218057	-2,045	0,000820	0,11685
ENSG00000049130	*KITLG*	-0,705	0,00064	0,113509	-0,690	0,000820	0,11685
ENSG00000158711	*ELK4*	0,700	0,00079	0,130387	0,697	0,000826	0,11734
ENSG00000116852	*KIF21B*	-0,716	0,00348	0,313575	-0,800	0,000829	0,11734
ENSG00000142552	*RCN3*	-1,699	3,67E-05	0,015172	-1,153	0,000829	0,11734
ENSG00000175197	*DDIT3*	-0,782	0,00072	0,121998	-0,771	0,000832	0,11753
ENSG00000122958	*VPS26A*	0,695	0,00117	0,166302	0,710	0,000835	0,11770
ENSG00000127946	*HIP1*	-0,696	0,00124	0,169037	-0,712	0,000855	0,12027
ENSG00000172893	*DHCR7*	0,691	0,00100	0,151774	0,697	0,000870	0,12212
ENSG00000164509	*IL31RA*	3,133	2,53E-05	0,011002	1,990	0,000878	0,12252
ENSG00000135108	*FBXO21*	-0,687	0,00122	0,168412	-0,704	0,000879	0,12252
ENSG00000245680	*ZNF585B*	-0,738	0,00256	0,264255	-0,794	0,000883	0,12279
ENSG00000268223	*ARL14EPL*	-0,763	0,00216	0,240710	-0,817	0,000889	0,12309
ENSG00000101000	*PROCR*	-0,777	0,00147	0,191022	-0,799	0,000892	0,12309
ENSG00000196632	*WNK3*	-1,216	0,00029	0,066932	-1,050	0,000894	0,12309
ENSG00000088340	*FER1L4*	0,973	0,00034	0,073642	0,876	0,000896	0,12309
ENSG00000104738	*MCM4*	0,664	0,00139	0,181436	0,689	0,000896	0,12309
ENSG00000163623	*NKX6-1*	-1,211	0,00076	0,128150	-1,139	0,000902	0,12370
ENSG00000154007	*ASB17*	2,458	0,00149	0,191813	2,532	0,000911	0,12418
ENSG00000090530	*P3H2*	-2,283	0,00243	0,256608	-2,439	0,000911	0,12418
ENSG00000203760	*CENPW*	-0,773	0,00095	0,147059	-0,764	0,000912	0,12418
ENSG00000115963	*RND3*	0,761	0,00059	0,107921	0,728	0,000931	0,12652
ENSG00000176919	*C8G*	-1,230	0,00292	0,284135	-1,275	0,000964	0,13000
ENSG00000177606	*JUN*	0,691	0,00095	0,147059	0,690	0,000971	0,13065
ENSG00000180901	*KCTD2*	-0,688	0,00124	0,169037	-0,701	0,000975	0,13089
ENSG00000067798	*NAV3*	0,689	0,00117	0,166302	0,696	0,000990	0,13262
ENSG00000176826	*FKBP9P1*	-0,639	0,00942	0,553283	-0,736	0,000992	0,13262
ENSG00000156299	*TIAM1*	-0,711	0,00150	0,193060	-0,729	0,000997	0,13262
ENSG00000225791	*TRAM2-AS1*	-1,022	0,00124	0,169037	-1,036	0,000998	0,13262
ENSG00000099822	*HCN2*	-0,872	0,00093	0,144999	-0,846	0,001006	0,13313
ENSG00000168646	*AXIN2*	-0,715	0,00186	0,221297	-0,749	0,001014	0,13336
ENSG00000177628	*GBA*	-0,717	0,00199	0,228792	-0,703	0,001015	0,13336
ENSG00000233276	*GPX1*	-0,673	0,00130	0,174109	-0,686	0,001016	0,13336
ENSG00000185090	*MANEAL*	-0,742	0,00110	0,161087	-0,738	0,001020	0,13359
ENSG00000179674	*ARL14*	1,304	0,00102	0,153684	1,308	0,001024	0,13385
ENSG00000130066	*SAT1*	0,698	0,00082	0,133631	0,684	0,001028	0,13395
ENSG00000154957	*ZNF18*	-0,799	0,00097	0,148826	-0,779	0,001050	0,13571
ENSG00000171227	*TMEM37*	-1,060	0,00151	0,193134	-1,089	0,001054	0,13571
ENSG00000115540	*MOB4*	-0,755	0,00082	0,132960	-0,737	0,001055	0,13571

ENSG00000139344	*AMDHD1*	-0,858	0,00466	0,376404	-0,973	0,001057	0,13571
ENSG00000257596	*SCAT2*	1,073	0,00106	0,156827	1,047	0,001057	0,13571
ENSG00000181481	*RNF135*	-0,838	0,00115	0,165378	-0,830	0,001064	0,13638
ENSG00000154511	*DIPK1A*	-0,837	0,00101	0,152510	-0,829	0,001072	0,13705
ENSG00000212907	*ND4L*	0,677	0,00100	0,151645	0,672	0,001088	0,13866
ENSG00000153029	*MR1*	-0,751	0,00166	0,207352	-0,771	0,001096	0,13938
ENSG00000139597	*N4BP2L1*	1,222	0,00058	0,107104	1,130	0,001100	0,13957
ENSG00000154645	*CHODL*	-1,086	0,00079	0,130387	-1,025	0,001108	0,14027
ENSG00000182771	*GRID1*	-0,991	0,00224	0,247113	-1,028	0,001114	0,14055
ENSG00000079150	*FKBP7*	-0,872	0,00196	0,227191	-0,891	0,001119	0,14089
ENSG00000251791	*SCARNA6*	-0,725	0,00454	0,372911	-0,754	0,001130	0,14142
ENSG00000156463	*SH3RF2*	0,747	0,00068	0,119741	0,710	0,001138	0,14190
ENSG00000136267	*DGKB*	-2,196	0,00394	0,339218	-3,146	0,001188	0,14666
ENSG00000166794	*PPIB*	-0,692	0,00109	0,159863	-0,683	0,001192	0,14693
ENSG00000113356	*POLR3G*	0,648	0,00209	0,235392	0,680	0,001198	0,14737
ENSG00000252010	*SCARNA5*	-0,637	0,00607	0,442616	-0,710	0,001205	0,14767
ENSG00000129538	*RNASE1*	0,849	0,00205	0,231989	0,890	0,001208	0,14774
ENSG00000158615	*PPP1R15B*	0,670	0,00124	0,169279	0,671	0,001213	0,14778
ENSG00000251562	*MALAT1*	0,665	0,00118	0,166302	0,664	0,001215	0,14778
ENSG00000089169	*RPH3A*	-2,212	0,00023	0,057074	-1,721	0,001222	0,14778
ENSG00000164220	*F2RL2*	1,718	0,00122	0,168412	1,722	0,001222	0,14778
ENSG00000127324	*TSPAN8*	-2,204	0,00170	0,210080	-1,987	0,001245	0,15037
ENSG00000166147	*FBN1*	0,968	0,00155	0,196790	0,951	0,001252	0,15073
ENSG00000117533	*VAMP4*	-0,802	0,00069	0,120029	-0,754	0,001255	0,15073
ENSG00000143515	*ATP8B2*	-0,654	0,00266	0,269022	-0,695	0,001255	0,15073
ENSG00000157315	*TMED6*	1,086	0,00134	0,176284	1,083	0,001261	0,15089
ENSG00000173868	*PHOSPHO1*	-1,372	0,00225	0,248636	-1,435	0,001266	0,15089
ENSG00000174776	*WDR49*	1,306	0,00932	0,549077	1,500	0,001266	0,15089
ENSG00000147041	*SYTL5*	6,365	0,00242	0,256608	6,491	0,001268	0,15089
ENSG00000227409	*ZMYM4-AS1*	-1,917	0,00128	0,172554	-1,913	0,001280	0,15146
ENSG00000172671	*ZFAND4*	-0,732	0,00117	0,166302	-0,719	0,001293	0,15269
ENSG00000083844	*ZNF264*	0,694	0,00112	0,162212	0,683	0,001303	0,15362
ENSG00000111962	*UST*	-0,881	0,00194	0,225542	-0,893	0,001306	0,15364
ENSG00000246705	*H2AJ*	1,177	0,00316	0,297099	1,265	0,001308	0,15364
ENSG00000187134	*AKR1C1*	1,343	0,00692	0,472689	1,375	0,001312	0,15387
ENSG00000115657	*ABCB6*	0,831	0,00112	0,162212	0,820	0,001330	0,15510
ENSG00000119922	*IFIT2*	-1,497	0,00104	0,155576	-1,445	0,001339	0,15588
ENSG00000183340	*JRKL*	0,759	0,00133	0,175633	0,758	0,001350	0,15682
ENSG00000187984	*ANKRD19P*	0,692	0,00156	0,197618	0,692	0,001354	0,15682
ENSG00000239264	*TXNDC5*	-0,822	0,00180	0,218057	-0,839	0,001373	0,15849
ENSG00000081189	*MEF2C*	0,690	0,00174	0,212332	0,702	0,001375	0,15849
ENSG00000132256	*TRIM5*	-0,691	0,00337	0,308675	-0,752	0,001378	0,15849
ENSG00000197106	*SLC6A17*	-0,724	0,00106	0,157007	-0,704	0,001381	0,15849
ENSG00000137767	*SQOR*	-1,075	0,00041	0,082975	-0,951	0,001384	0,15849
ENSG00000069812	*HES2*	-0,774	0,00189	0,221984	-0,792	0,001386	0,15849
ENSG00000115884	*SDC1*	-0,755	0,00076	0,128760	-0,715	0,001386	0,15849
ENSG00000066629	*EML1*	-0,852	0,00070	0,121338	-0,769	0,001399	0,15966
ENSG00000110042	*DTX4*	-3,764	0,00141	0,183883	-3,760	0,001410	0,16015
ENSG00000118473	*SGIP1*	-1,017	0,00294	0,284228	-1,060	0,001417	0,16062
ENSG00000152332	*UHMK1*	0,669	0,00122	0,168412	0,660	0,001433	0,16216
ENSG00000163491	*NEK10*	0,947	0,00035	0,074206	0,809	0,001454	0,16429
ENSG00000171889	*MIR31HG*	-1,028	0,00149	0,191882	-1,021	0,001473	0,16515
ENSG00000196505	*GDAP2*	-0,685	0,00144	0,187405	-0,680	0,001483	0,16515
ENSG00000160862	*AZGP1*	-2,959	0,00339	0,308675	-2,761	0,001488	0,16515
ENSG00000184005	*ST6GALNAC3*	-1,396	0,00278	0,274733	-1,470	0,001492	0,16515
ENSG00000186115	*CYP4F2*	1,805	0,00904	0,545404	1,521	0,001492	0,16515

ENSG00000167005	*NUDT21*	-0,673	0,00126	0,171212	-0,662	0,001504	0,16607
ENSG00000133121	*STARD13*	-0,831	0,00205	0,232248	-0,845	0,001516	0,16712
ENSG00000197763	*TXNRD3*	-0,819	0,00130	0,173646	-0,786	0,001536	0,16883
ENSG00000138175	*ARL3*	-0,648	0,00484	0,388170	-0,710	0,001554	0,17017
ENSG00000244187	*TMEM141*	-0,715	0,00251	0,261176	-0,749	0,001562	0,17076
ENSG00000132589	*FLOT2*	-0,621	0,00359	0,320056	-0,671	0,001575	0,17196
ENSG00000166123	*GPT2*	-0,713	0,00121	0,168412	-0,689	0,001589	0,17214
ENSG00000066654	*THUMPD1*	0,667	0,00153	0,195561	0,663	0,001589	0,17214
ENSG00000131652	*THOC6*	-0,681	0,00218	0,242448	-0,685	0,001593	0,17214
ENSG00000179163	*FUCA1*	-0,677	0,00550	0,419266	-0,752	0,001596	0,17214
ENSG00000215256	*DHRS4-AS1*	-0,755	0,00738	0,489937	-0,774	0,001608	0,17266
ENSG00000176102	*CSTF3*	0,663	0,00173	0,211846	0,662	0,001612	0,17273
ENSG00000075891	*PAX2*	-1,098	0,00331	0,306180	-1,164	0,001621	0,17317
ENSG00000246922	*UBAP1L*	0,715	0,00129	0,173001	0,700	0,001629	0,17357
ENSG00000078401	*EDN1*	-1,028	0,00541	0,417198	-1,149	0,001630	0,17357
ENSG00000119121	*TRPM6*	-0,864	0,00155	0,196920	-0,825	0,001648	0,17514
ENSG00000184164	*CRELD2*	-0,700	0,00189	0,221984	-0,692	0,001654	0,17536
ENSG00000121064	*SCPEP1*	-0,693	0,00243	0,256687	-0,703	0,001655	0,17536
ENSG00000111052	*LIN7A*	-0,953	0,00324	0,301965	-1,012	0,001663	0,17585
ENSG00000130164	*LDLR*	0,657	0,00155	0,196790	0,651	0,001674	0,17650
ENSG00000140948	*ZCCHC14*	-0,646	0,00204	0,231739	-0,657	0,001681	0,17696
ENSG00000140941	*MAP1LC3B*	-0,717	0,00071	0,121539	-0,661	0,001684	0,17701
ENSG00000144591	*GMPPA*	-0,619	0,00456	0,372977	-0,674	0,001730	0,17902
ENSG00000118922	*KLF12*	-0,778	0,00182	0,218057	-0,779	0,001732	0,17902
ENSG00000159753	*CARMIL2*	-0,709	0,00287	0,280962	-0,724	0,001733	0,17902
ENSG00000170175	*CHRNB1*	-0,832	0,00696	0,472704	-0,796	0,001742	0,17902
ENSG00000166349	*RAG1*	-1,190	0,00123	0,168412	-1,147	0,001745	0,17902
ENSG00000025423	*HSD17B6*	-0,939	0,00364	0,322153	-0,984	0,001770	0,18068
ENSG00000008513	*ST3GAL1*	-0,750	0,00171	0,211303	-0,739	0,001777	0,18114
ENSG00000188107	*EYS*	0,790	0,00132	0,174687	0,770	0,001780	0,18116
ENSG00000065717	*TLE2*	-0,646	0,00433	0,361448	-0,689	0,001806	0,18251
ENSG00000226710	*SNHG32*	-0,691	0,00187	0,221520	-0,649	0,001807	0,18251
ENSG00000136295	*TTYH3*	-0,653	0,00273	0,272588	-0,677	0,001842	0,18406
ENSG00000196277	*GRM7*	-1,182	0,00232	0,252112	-1,160	0,001863	0,18539
ENSG00000110324	*IL10RA*	-1,151	0,00174	0,212332	-1,132	0,001864	0,18539
ENSG00000169239	*CA5B*	-0,758	0,00262	0,268087	-0,752	0,001884	0,18666
ENSG00000117899	*MESD*	-0,632	0,00274	0,272588	-0,654	0,001885	0,18666
ENSG00000012171	*SEMA3B*	0,819	0,00181	0,218057	0,789	0,001900	0,18755
ENSG00000207008	*SNORA54*	-0,988	0,00407	0,347000	-0,844	0,001907	0,18760
ENSG00000198910	*L1CAM*	-0,666	0,00404	0,345342	-0,698	0,001915	0,18804
ENSG00000173218	*VANGL1*	-0,695	0,00119	0,166596	-0,663	0,001917	0,18804
ENSG00000081041	*CXCL2*	-1,137	0,00921	0,546800	-1,282	0,001921	0,18805
ENSG00000128266	*GNAZ*	-0,895	0,00084	0,135270	-0,829	0,001923	0,18805
ENSG00000092871	*RFFL*	0,645	0,00319	0,298668	0,675	0,001955	0,19089
ENSG00000163734	*CXCL3*	-1,299	0,00366	0,323163	-1,355	0,001961	0,19120
ENSG00000248508	*SRP14-AS1*	0,883	0,00132	0,174687	0,829	0,002001	0,19419
ENSG00000111981	*ULBP1*	-0,817	0,00062	0,110318	-0,722	0,002016	0,19541
ENSG00000105976	*MET*	-0,630	0,00247	0,260117	-0,642	0,002022	0,19563
ENSG00000168026	*TTC21A*	-0,786	0,00419	0,354973	-0,814	0,002028	0,19592
ENSG00000277449	*CEBPB-AS1*	1,484	0,00204	0,231739	1,488	0,002039	0,19647
ENSG00000140511	*HAPLN3*	-0,917	0,00721	0,483267	-0,991	0,002046	0,19651
ENSG00000140465	*CYP1A1*	0,867	0,00505	0,398029	0,916	0,002064	0,19768
ENSG00000139998	*RAB15*	-0,684	0,00368	0,324297	-0,721	0,002070	0,19794
ENSG00000064652	*SNX24*	-0,862	0,00071	0,121539	-0,754	0,002097	0,20000
ENSG00000165655	*ZNF503*	-0,676	0,00214	0,238693	-0,677	0,002098	0,20000
ENSG00000130724	*CHMP2A*	-0,690	0,00133	0,176220	-0,657	0,002130	0,20275

ENSG00000217801	*LOC100288175*	0,832	0,00441	0,365118	0,874	0,002159	0,20512
ENSG00000135924	*DNAJB2*	-0,684	0,00173	0,211846	-0,664	0,002161	0,20512
ENSG00000164211	*STARD4*	0,633	0,00273	0,272588	0,647	0,002164	0,20512
ENSG00000108387	*SEPTIN4*	-0,876	0,00187	0,221520	-0,840	0,002173	0,20565
ENSG00000073792	*IGF2BP2*	-0,634	0,00248	0,260530	-0,639	0,002199	0,20782
ENSG00000255150	*EID3*	0,703	0,00248	0,260665	0,707	0,002239	0,21129
ENSG00000167535	*CACNB3*	-0,699	0,00312	0,294543	-0,712	0,002247	0,21177
ENSG00000140931	*CMTM3*	-0,645	0,00332	0,306208	-0,669	0,002256	0,21199
ENSG00000132196	*HSD17B7*	0,679	0,00388	0,336538	0,687	0,002262	0,21212
ENSG00000110756	*HPS5*	0,632	0,00295	0,284475	0,646	0,002264	0,21212
ENSG00000139372	*TDG*	0,675	0,00170	0,210218	0,646	0,002291	0,21302
ENSG00000154553	*PDLIM3*	-0,655	0,00447	0,368468	-0,702	0,002293	0,21302
ENSG00000196159	*FAT4*	-1,611	0,00229	0,250743	-1,607	0,002293	0,21302
ENSG00000141179	*PCTP*	-0,661	0,00669	0,463358	-0,727	0,002321	0,21532
ENSG00000171617	*ENC1*	0,898	0,00160	0,200695	0,848	0,002343	0,21668
ENSG00000179361	*ARID3B*	0,733	0,00114	0,164734	0,670	0,002347	0,21681
ENSG00000112320	*SOBP*	-0,730	0,00179	0,216765	-0,701	0,002354	0,21686
ENSG00000134352	*IL6ST*	-0,635	0,00295	0,284475	-0,646	0,002355	0,21686
ENSG00000102057	*KCND1*	0,691	0,00318	0,298646	0,709	0,002363	0,21705
ENSG00000254206	*NPIPB11*	0,620	0,00904	0,545404	0,647	0,002380	0,21822
ENSG00000178695	*KCTD12*	-1,015	0,00239	0,256608	-1,010	0,002390	0,21890
ENSG00000087245	*MMP2*	-6,310	0,00242	0,256608	-6,308	0,002421	0,21898
ENSG00000240057	*LOC107986114*	3,790	0,00025	0,060633	2,389	0,002426	0,21898
ENSG00000128610	*FEZF1*	-2,283	0,00243	0,256608	-2,279	0,002426	0,21898
ENSG00000197147	*LRRC8B*	-0,629	0,00297	0,285132	-0,641	0,002428	0,21898
ENSG00000074211	*PPP2R2C*	-0,660	0,00281	0,276782	-0,668	0,002439	0,21961
ENSG00000174871	*CNIH2*	-1,006	0,00657	0,460021	-1,108	0,002446	0,21995
ENSG00000176239	*OR51B6*	0,734	0,00322	0,300519	0,748	0,002468	0,22073
ENSG00000213901	*SLC23A3*	1,114	0,00306	0,290952	1,138	0,002481	0,22073
ENSG00000095596	*CYP26A1*	3,210	0,00788	0,507885	3,434	0,002504	0,22147
ENSG00000125741	*OPA3*	0,686	0,00175	0,212332	0,660	0,002525	0,22305
ENSG00000101115	*SALL4*	0,919	0,00568	0,429149	0,986	0,002569	0,22570
ENSG00000196352	*CD55*	0,628	0,00310	0,293084	0,636	0,002591	0,22730
ENSG00000119541	*VPS4B*	0,657	0,00199	0,229152	0,637	0,002602	0,22775
ENSG00000131969	*ABHD12B*	0,760	0,00289	0,281845	0,765	0,002603	0,22775
ENSG00000086619	*ERO1B*	-0,801	0,00626	0,451791	-0,843	0,002623	0,22885
ENSG00000134901	*POGLUT2*	-0,743	0,00253	0,262892	-0,718	0,002632	0,22940
ENSG00000158292	*GPR153*	-0,920	0,00167	0,207600	-0,876	0,002640	0,22974
ENSG00000187498	*COL4A1*	2,377	0,00182	0,218057	1,756	0,002661	0,22993
ENSG00000074370	*ATP2A3*	-0,682	0,00621	0,449675	-0,732	0,002663	0,22993
ENSG00000250748	*LOC105369187*	-0,879	0,00376	0,328910	-0,907	0,002672	0,22994
ENSG00000005059	*MCUB*	-0,644	0,00535	0,414084	-0,684	0,002674	0,22994
ENSG00000234028	*EIF2AK3-DT*	-1,138	0,00250	0,261176	-1,117	0,002674	0,22994
ENSG00000134107	*BHLHE40*	0,636	0,00259	0,266198	0,633	0,002688	0,23053
ENSG00000185269	*NOTUM*	-0,766	0,00703	0,476479	-0,823	0,002702	0,23143
ENSG00000135622	*SEMA4F*	-0,681	0,00298	0,285277	-0,677	0,002730	0,23351
ENSG00000197405	*C5AR1*	0,902	0,00221	0,244414	0,846	0,002754	0,23528
ENSG00000180113	*TDRD6*	-0,909	0,00191	0,223145	-0,871	0,002787	0,23773
ENSG00000162430	*SELENON*	-0,610	0,00389	0,336578	-0,630	0,002796	0,23819
ENSG00000236756	*DNAJC9-AS1*	1,125	0,00331	0,306180	1,108	0,002837	0,24044
ENSG00000106799	*TGFBR1*	-0,649	0,00250	0,261176	-0,639	0,002853	0,24121
ENSG00000147383	*NSDHL*	0,640	0,00361	0,320056	0,651	0,002864	0,24176
ENSG00000186907	*RTN4RL2*	-0,715	0,00547	0,419173	-0,759	0,002922	0,24597
ENSG00000053747	*LAMA3*	0,721	0,00204	0,231739	0,678	0,002925	0,24597
ENSG00000196923	*PDLIM7*	0,634	0,00292	0,284135	0,629	0,002946	0,24738
ENSG00000198576	*ARC*	-0,732	0,00261	0,267402	-0,714	0,002956	0,24790

ENSG00000285280	*LOC105371664*	0,695	0,00329	0,306020	0,696	0,003001	0,25077
ENSG00000272886	*DCP1A*	0,624	0,00309	0,293084	0,624	0,003019	0,25189
ENSG00000011021	*CLCN6*	0,633	0,00361	0,320056	0,641	0,003059	0,25486
ENSG00000198074	*AKR1B10*	0,994	0,00098	0,149278	0,824	0,003068	0,25502
ENSG00000166016	*ABTB2*	0,634	0,00454	0,372911	0,654	0,003122	0,25888
ENSG00000198786	*ND5*	0,609	0,00300	0,286708	0,606	0,003150	0,25916
ENSG00000272533	*SNORA28*	-1,230	0,00316	0,297099	-1,225	0,003162	0,25916
ENSG00000117335	*CD46*	0,572	0,00613	0,444932	0,614	0,003222	0,26219
ENSG00000147606	*SLC26A7*	0,736	0,00689	0,472689	0,789	0,003223	0,26219
ENSG00000231679	*HLA-DRB3*	-1,396	0,00278	0,274733	-0,949	0,003237	0,26266
ENSG00000153790	*C7orf31*	-0,939	0,00603	0,442503	-0,949	0,003237	0,26266
ENSG00000111817	*DSE*	-0,598	0,00554	0,421580	-0,634	0,003251	0,26352
ENSG00000106537	*TSPAN13*	-0,676	0,00388	0,336538	-0,679	0,003265	0,26386
ENSG00000108784	*NAGLU*	-0,789	0,00269	0,270476	-0,771	0,003267	0,26386
ENSG00000198888	*ND1*	0,595	0,00374	0,328558	0,603	0,003272	0,26386
ENSG00000155729	*KCTD18*	-0,733	0,00306	0,290952	-0,712	0,003272	0,26386
ENSG00000167524	*RSKR*	0,642	0,00491	0,392341	0,670	0,003286	0,26470
ENSG00000207205	*RNVU1-15*	-1,098	0,00331	0,306180	-1,094	0,003310	0,26629
ENSG00000131845	*ZNF304*	0,772	0,00383	0,333496	0,782	0,003381	0,27060
ENSG00000282773	*GOLGA8Q*	-1,729	0,00963	0,553283	-0,940	0,003409	0,27217
ENSG00000196878	*LAMB3*	0,633	0,00281	0,276782	0,617	0,003439	0,27425
ENSG00000144647	*POMGNT2*	-0,639	0,00361	0,320056	-0,642	0,003461	0,27517
ENSG00000180891	*CUEDC1*	0,681	0,00193	0,224873	0,638	0,003466	0,27517
ENSG00000169962	*TAS1R3*	0,963	0,00347	0,313111	0,967	0,003467	0,27517
ENSG00000170381	*SEMA3E*	-0,922	0,00902	0,545125	-0,971	0,003467	0,27517
ENSG00000147166	*ITGB1BP2*	0,830	0,00227	0,249115	0,790	0,003481	0,27532
ENSG00000163755	*HPS3*	-0,650	0,00495	0,393445	-0,664	0,003483	0,27532
ENSG00000177692	*DNAJC28*	-1,269	0,00631	0,452129	-1,079	0,003486	0,27532
ENSG00000177042	*TMEM80*	0,721	0,00227	0,249115	0,691	0,003511	0,27659
ENSG00000138756	*BMP2K*	-0,648	0,00432	0,361448	-0,654	0,003520	0,27694
ENSG00000180537	*RNF182*	-0,757	0,00297	0,285132	-0,744	0,003525	0,27699
ENSG00000147394	*ZNF185*	-0,659	0,00803	0,515711	-0,701	0,003547	0,27842
ENSG00000277897	*GSTT2*	-1,497	0,00104	0,155576	-0,782	0,003565	0,27950
ENSG00000153814	*JAZF1*	-0,889	0,00160	0,200695	-0,805	0,003572	0,27952
ENSG00000095209	*TMEM38B*	-0,648	0,00426	0,358521	-0,649	0,003578	0,27952
ENSG00000189007	*ADAT2*	0,623	0,00393	0,339218	0,628	0,003649	0,28469
ENSG00000212232	*SNORD17*	-0,618	0,00349	0,313575	-0,615	0,003692	0,28775
ENSG00000167291	*TBC1D16*	-0,620	0,00417	0,353497	-0,628	0,003703	0,28825
ENSG00000252835	*SCARNA21*	-0,633	0,00513	0,401947	-0,654	0,003717	0,28861
ENSG00000280852	*LOC653653*	-0,730	0,00686	0,471847	-0,739	0,003728	0,28878
ENSG00000149948	*HMGA2*	-0,599	0,00382	0,332734	-0,599	0,003751	0,29018
ENSG00000049283	*EPN3*	0,679	0,00611	0,444609	0,709	0,003760	0,29054
ENSG00000090013	*BLVRB*	-0,592	0,00850	0,532706	-0,646	0,003765	0,29061
ENSG00000168564	*CDKN2AIP*	-0,661	0,00437	0,363012	-0,669	0,003771	0,29069
ENSG00000105520	*PLPPR2*	-0,592	0,00880	0,543516	-0,647	0,003792	0,29167
ENSG00000091409	*ITGA6*	-0,569	0,00610	0,444609	-0,599	0,003817	0,29323
ENSG00000172379	*ARNT2*	-0,750	0,00229	0,250743	-0,696	0,003826	0,29358
ENSG00000205133	*TRIQK*	-0,605	0,00812	0,520696	-0,656	0,003840	0,29371
ENSG00000007314	*SCN4A*	-0,900	0,00083	0,134594	-0,762	0,003851	0,29371
ENSG00000159200	*RCAN1*	-0,715	0,00302	0,288798	-0,691	0,003858	0,29394
ENSG00000125484	*GTF3C4*	0,595	0,00411	0,349852	0,598	0,003872	0,29469
ENSG00000162616	*DNAJB4*	0,620	0,00350	0,313575	0,611	0,003906	0,29659
ENSG00000088826	*SMOX*	-0,680	0,00877	0,543516	-0,734	0,003929	0,29659
ENSG00000115520	*COQ10B*	0,640	0,00498	0,394261	0,649	0,003943	0,29659
ENSG00000264585	*MIR4449*	-2,196	0,00394	0,339218	-2,192	0,003945	0,29659
ENSG00000232442	*MHENCR*	1,153	0,00201	0,229784	1,061	0,003948	0,29659

ENSG00000071537	*SEL1L*	-0,582	0,00549	0,419266	-0,603	0,003952	0,29659
ENSG00000135519	*KCNH3*	-0,651	0,00340	0,308983	-0,636	0,003983	0,29785
ENSG00000163577	*EIF5A2*	0,621	0,00659	0,460736	0,652	0,003987	0,29785
ENSG00000143147	*GPR161*	-0,683	0,00295	0,284475	-0,655	0,004010	0,29922
ENSG00000130812	*ANGPTL6*	-1,573	0,00841	0,529056	-1,689	0,004015	0,29924
ENSG00000123908	*AGO2*	0,601	0,00351	0,313897	0,591	0,004076	0,30306
ENSG00000185338	*SOCS1*	1,085	0,00505	0,398029	1,109	0,004091	0,30385
ENSG00000161970	*RPL26*	0,599	0,00378	0,330190	0,594	0,004115	0,30530
ENSG00000197043	*ANXA6*	-0,600	0,00501	0,395734	-0,603	0,004204	0,30957
ENSG00000119411	*BSPRY*	0,666	0,00320	0,299360	0,643	0,004214	0,30957
ENSG00000159899	*NPR2*	0,664	0,00653	0,458740	0,682	0,004219	0,30957
ENSG00000167004	*PDIA3*	-0,582	0,00495	0,393445	-0,590	0,004229	0,30957
ENSG00000006606	*CCL26*	-2,765	0,00149	0,191813	-2,049	0,004230	0,30957
ENSG00000092929	*UNC13D*	0,728	0,00101	0,152361	0,624	0,004236	0,30964
ENSG00000148926	*ADM*	0,680	0,00537	0,414746	0,691	0,004273	0,31129
ENSG00000205111	*CDKL4*	2,307	0,00394	0,339218	2,067	0,004296	0,31260
ENSG00000116704	*SLC35D1*	-0,648	0,00333	0,306878	-0,628	0,004313	0,31330
ENSG00000222009	*BTBD19*	0,837	0,00653	0,458740	0,877	0,004319	0,31330
ENSG00000183091	*NEB*	0,596	0,00694	0,472704	0,622	0,004322	0,31330
ENSG00000126467	*TSKS*	2,193	0,00433	0,361448	2,197	0,004327	0,31330
ENSG00000077063	*CTTNBP2*	-0,861	0,00108	0,159074	-0,740	0,004330	0,31330
ENSG00000148468	*FAM171A1*	-0,602	0,00440	0,365041	-0,602	0,004354	0,31439
ENSG00000073910	*FRY*	-1,512	0,00109	0,159822	-1,258	0,004416	0,31634
ENSG00000074842	*MYDGF*	-0,623	0,00424	0,357121	-0,608	0,004456	0,31747
ENSG00000104936	*DMPK*	0,622	0,00579	0,431875	0,637	0,004479	0,31843
ENSG00000105298	*CACTIN*	0,603	0,00582	0,433281	0,617	0,004499	0,31947
ENSG00000159733	*ZFYVE28*	-0,673	0,00626	0,451684	-0,681	0,004519	0,32054
ENSG00000171824	*EXOSC10*	0,584	0,00536	0,414084	0,593	0,004529	0,32090
ENSG00000140009	*ESR2*	0,927	0,00491	0,392341	0,914	0,004574	0,32341
ENSG00000136630	*HLX*	-1,127	0,00231	0,251600	-0,999	0,004602	0,32433
ENSG00000135404	*CD63*	-0,586	0,00592	0,436538	-0,598	0,004626	0,32498
ENSG00000227533	*SLC2A1-AS1*	-1,020	0,00837	0,528936	-1,052	0,004643	0,32498
ENSG00000175967	*FLJ34503*	6,229	0,00464	0,376147	6,231	0,004643	0,32498
ENSG00000103742	*IGDCC4*	-0,981	0,00337	0,308675	-0,909	0,004653	0,32498
ENSG00000168528	*SERINC2*	-0,704	0,00350	0,313575	-0,668	0,004659	0,32498
ENSG00000248323	*LUCAT1*	0,647	0,00274	0,272638	0,609	0,004666	0,32498
ENSG00000120137	*PANK3*	0,591	0,00434	0,361708	0,586	0,004679	0,32547
ENSG00000116017	*ARID3A*	0,634	0,00385	0,334490	0,617	0,004696	0,32635
ENSG00000181381	*DDX60L*	-0,595	0,00722	0,483481	-0,618	0,004722	0,32696
ENSG00000148803	*FUOM*	-0,766	0,00568	0,429149	-0,760	0,004775	0,33006
ENSG00000278695	*GSTT2B*	-1,229	0,00576	0,431074	-0,778	0,004894	0,33794
ENSG00000178607	*ERN1*	-0,617	0,00595	0,437028	-0,626	0,004938	0,34024
ENSG00000198715	*GLMP*	-0,640	0,00657	0,460021	-0,649	0,004956	0,34105
ENSG00000120149	*MSX2*	0,617	0,00534	0,414004	0,618	0,004960	0,34105
ENSG00000101986	*ABCD1*	0,773	0,00491	0,392341	0,756	0,004982	0,34211
ENSG00000101439	*CST3*	-0,627	0,00408	0,348007	-0,610	0,005013	0,34321
ENSG00000177565	*TBL1XR1*	-0,567	0,00617	0,446615	-0,580	0,005019	0,34325
ENSG00000137198	*GMPR*	-0,725	0,00897	0,543831	-0,737	0,005040	0,34360
ENSG00000151012	*SLC7A11*	0,631	0,00304	0,289389	0,595	0,005060	0,34398
ENSG00000203780	*FANK1*	0,809	0,00211	0,236565	0,721	0,005072	0,34435
ENSG00000246145	*RRS1-AS1*	1,584	0,00510	0,400335	1,588	0,005100	0,34590
ENSG00000110660	*SLC35F2*	-0,577	0,00593	0,436781	-0,586	0,005138	0,34812
ENSG00000081052	*COL4A4*	-0,598	0,00927	0,548190	-0,632	0,005146	0,34822
ENSG00000102580	*DNAJC3*	-0,595	0,00457	0,372977	-0,585	0,005150	0,34822
ENSG00000177707	*NECTIN3*	-0,574	0,00758	0,497704	-0,601	0,005170	0,34842
ENSG00000196890	*H2BU1*	-0,767	0,00046	0,089279	-0,589	0,005191	0,34913

ENSG00000104635	*SLC39A14*	0,583	0,00522	0,407110	0,582	0,005268	0,35212
ENSG00000162490	*DRAXIN*	-0,917	0,00721	0,483267	-0,900	0,005277	0,35212
ENSG00000094631	*HDAC6*	0,591	0,00514	0,402553	0,586	0,005279	0,35212
ENSG00000143870	*PDIA6*	-0,605	0,00404	0,345342	-0,582	0,005286	0,35221
ENSG00000170421	*KRT8*	0,540	0,00870	0,543066	0,573	0,005336	0,35486
ENSG00000116667	*C1orf21*	-0,803	0,00475	0,382537	-0,768	0,005406	0,35735
ENSG00000143156	*NME7*	0,632	0,00909	0,546266	0,638	0,005409	0,35735
ENSG00000099251	*HSD17B7P2*	0,792	0,00506	0,398029	0,692	0,005418	0,35735
ENSG00000260231	*KDM7A-DT*	-1,749	0,00276	0,273450	-1,595	0,005464	0,35735
ENSG00000187391	*MAGI2*	-1,543	0,00779	0,505555	-1,595	0,005464	0,35735
ENSG00000124493	*GRM4*	-0,837	0,00580	0,432209	-0,820	0,005484	0,35735
ENSG00000110092	*CCND1*	0,571	0,00545	0,418404	0,571	0,005511	0,35860
ENSG00000126091	*ST3GAL3*	0,635	0,00956	0,553283	0,676	0,005514	0,35860
ENSG00000112379	*ARFGEF3*	-0,620	0,00480	0,385999	-0,605	0,005564	0,36085
ENSG00000170899	*GSTA4*	-1,022	0,00588	0,436294	-1,014	0,005565	0,36085
ENSG00000131473	*ACLY*	0,573	0,00549	0,419266	0,571	0,005599	0,36235
ENSG00000174448	*STARD6*	-1,328	0,00258	0,265204	-1,195	0,005649	0,36516
ENSG00000127364	*TAS2R4*	0,777	0,00275	0,272973	0,715	0,005678	0,36633
ENSG00000070081	*NUCB2*	-0,633	0,00436	0,362477	-0,605	0,005688	0,36658
ENSG00000022567	*SLC45A4*	-0,622	0,00420	0,355129	-0,598	0,005758	0,37000
ENSG00000143797	*MBOAT2*	-0,600	0,00576	0,431074	-0,596	0,005768	0,37030
ENSG00000144893	*MED12L*	-0,627	0,00783	0,506975	-0,635	0,005800	0,37197
ENSG00000185245	*GP1BA*	0,719	0,00526	0,408718	0,708	0,005856	0,37484
ENSG00000164061	*BSN*	-0,649	0,00465	0,376147	-0,629	0,005876	0,37572
ENSG00000136870	*ZNF189*	-0,621	0,00590	0,436294	-0,616	0,005923	0,37835
ENSG00000063180	*CA11*	-0,761	0,00287	0,280903	-0,680	0,005929	0,37835
ENSG00000181609	*OR52D1*	0,597	0,00638	0,453888	0,600	0,005973	0,37996
ENSG00000103021	*CCDC113*	-0,586	0,00715	0,482430	-0,597	0,005975	0,37996
ENSG00000189045	*ANKDD1B*	1,124	0,00835	0,528936	1,139	0,005993	0,37996
ENSG00000103269	*RHBDL1*	0,887	0,00163	0,204679	0,754	0,005994	0,37996
ENSG00000137841	*PLCB2*	0,706	0,00589	0,436294	0,690	0,005995	0,37996
ENSG00000001460	*STPG1*	-0,611	0,00917	0,546800	-0,635	0,006008	0,38006
ENSG00000072952	*MRVI1*	-0,606	0,00630	0,452129	-0,603	0,006021	0,38051
ENSG00000048540	*LMO3*	0,904	0,00420	0,355129	0,870	0,006041	0,38103
ENSG00000117394	*SLC2A1*	-0,566	0,00653	0,458740	-0,570	0,006070	0,38217
ENSG00000023608	*SNAPC1*	0,606	0,00882	0,543516	0,625	0,006178	0,38775
ENSG00000006638	*TBXA2R*	-0,935	0,00207	0,233746	-0,826	0,006198	0,38826
ENSG00000139644	*TMBIM6*	-0,585	0,00459	0,374068	-0,564	0,006228	0,38979
ENSG00000106780	*MEGF9*	-0,604	0,00735	0,489186	-0,615	0,006252	0,39092
ENSG00000172638	*EFEMP2*	0,785	0,00840	0,529056	0,788	0,006291	0,39255
ENSG00000154146	*NRGN*	-0,734	0,00246	0,259453	-0,658	0,006300	0,39255
ENSG00000113328	*CCNG1*	-0,577	0,00695	0,472704	-0,581	0,006314	0,39278
ENSG00000110148	*CCKBR*	-3,870	0,00079	0,130387	-2,499	0,006393	0,39590
ENSG00000248596	*LOC643201*	2,866	0,00119	0,166302	2,230	0,006393	0,39590
ENSG00000174705	*SH3PXD2B*	-0,608	0,00495	0,393445	-0,590	0,006442	0,39719
ENSG00000149428	*HYOU1*	-0,605	0,00379	0,330624	-0,562	0,006458	0,39763
ENSG00000181577	*C6orf223*	0,723	0,00471	0,379560	0,698	0,006507	0,40023
ENSG00000173114	*LRRN3*	0,752	0,00501	0,395734	0,730	0,006512	0,40023
ENSG00000198727	*CYTB*	0,560	0,00632	0,452129	0,558	0,006520	0,40029
ENSG00000141519	*CCDC40*	-0,636	0,00734	0,489056	-0,632	0,006552	0,40092
ENSG00000104848	*KCNA7*	1,478	0,00661	0,460736	1,482	0,006607	0,40092
ENSG00000100439	*ABHD4*	0,674	0,00346	0,312888	0,617	0,006607	0,40092
ENSG00000156194	*PPEF2*	2,784	0,00201	0,229784	2,131	0,006630	0,40092
ENSG00000120949	*TNFRSF8*	-2,283	0,00243	0,256608	-1,869	0,006630	0,40092
ENSG00000177679	*SRRM3*	-0,628	0,00754	0,496006	-0,627	0,006642	0,40092
ENSG00000185947	*ZNF267*	-0,592	0,00640	0,453888	-0,587	0,006661	0,40111

ENSG00000102781	*KATNAL1*	-0,606	0,00637	0,453888	-0,599	0,006736	0,40371
ENSG00000137941	*TTLL7*	-0,657	0,00237	0,256608	-0,579	0,006770	0,40455
ENSG00000272695	*GAS6-DT*	-1,229	0,00632	0,452129	-1,192	0,006804	0,40573
ENSG00000106261	*ZKSCAN1*	-0,557	0,00728	0,486777	-0,561	0,006814	0,40573
ENSG00000155304	*HSPA13*	-0,588	0,00606	0,442616	-0,578	0,006829	0,40573
ENSG00000233101	*HOXB-AS3*	0,758	0,00497	0,394125	0,728	0,006855	0,40629
ENSG00000128602	*SMO*	-0,659	0,00456	0,372977	-0,619	0,006882	0,40751
ENSG00000183161	*FANCF*	-1,288	0,00206	0,233136	-1,063	0,006902	0,40804
ENSG00000124766	*SOX4*	-0,552	0,00770	0,501580	-0,559	0,006945	0,40897
ENSG00000243660	*ZNF487*	-0,825	0,00745	0,492701	-0,828	0,006955	0,40922
ENSG00000153093	*ACOXL*	-0,792	0,00909	0,546266	-0,744	0,006983	0,40984
ENSG00000134709	*HOOK1*	-0,601	0,00607	0,442616	-0,586	0,006985	0,40984
ENSG00000140398	*NEIL1*	0,684	0,00401	0,343814	0,631	0,007003	0,41017
ENSG00000185924	*RTN4RL1*	-0,751	0,00828	0,525655	-0,763	0,007025	0,41070
ENSG00000119138	*KLF9*	-0,761	0,00943	0,553283	-0,786	0,007049	0,41154
ENSG00000213903	*LTB4R*	0,692	0,00376	0,328910	0,603	0,007074	0,41184
ENSG00000149084	*HSD17B12*	0,575	0,00750	0,493665	0,577	0,007101	0,41291
ENSG00000170759	*KIF5B*	0,564	0,00623	0,450118	0,553	0,007182	0,41613
ENSG00000167315	*ACAA2*	-0,717	0,00365	0,322153	-0,651	0,007259	0,41871
ENSG00000006062	*MAP3K14*	0,573	0,00821	0,523212	0,578	0,007304	0,42067
ENSG00000244165	*P2RY11*	0,640	0,00838	0,528936	0,646	0,007306	0,42067
ENSG00000151320	*AKAP6*	-0,639	0,00991	0,562715	-0,664	0,007322	0,42082
ENSG00000250420	*AACSP1*	0,674	0,00879	0,543516	0,679	0,007328	0,42082
ENSG00000165181	*SHOC1*	0,678	0,00909	0,546266	0,693	0,007368	0,42276
ENSG00000128989	*ARPP19*	0,535	0,00979	0,557372	0,554	0,007447	0,42515
ENSG00000196154	*S100A4*	0,676	0,00675	0,466781	0,651	0,007449	0,42515
ENSG00000073756	*PTGS2*	-1,196	0,00680	0,468843	-1,110	0,007583	0,43101
ENSG00000101977	*MCF2*	1,584	0,00510	0,400335	1,386	0,007595	0,43101
ENSG00000040608	*RTN4R*	-0,660	0,00765	0,499236	-0,657	0,007616	0,43109
ENSG00000159176	*CSRP1*	-0,553	0,00913	0,546800	-0,564	0,007741	0,43584
ENSG00000198840	*ND3*	0,552	0,00727	0,486201	0,547	0,007813	0,43777
ENSG00000088298	*EDEM2*	-0,675	0,00423	0,356690	-0,612	0,007840	0,43777
ENSG00000133739	*LRRCC1*	-0,620	0,00451	0,371628	-0,574	0,007842	0,43777
ENSG00000100505	*TRIM9*	-0,630	0,00922	0,546800	-0,633	0,007860	0,43777
ENSG00000169427	*KCNK9*	-3,390	0,00788	0,507885	-3,386	0,007880	0,43777
ENSG00000110723	*EXPH5*	0,569	0,00640	0,453888	0,554	0,007886	0,43777
ENSG00000186591	*UBE2H*	0,551	0,00901	0,545060	0,558	0,007972	0,44114
ENSG00000104213	*PDGFRL*	-0,897	0,00568	0,429149	-0,814	0,008000	0,44114
ENSG00000189221	*MAOA*	-0,640	0,00974	0,556839	-0,640	0,008008	0,44114
ENSG00000235823	*OLMALINC*	0,623	0,00662	0,460736	0,603	0,008030	0,44196
ENSG00000167491	*GATAD2A*	0,538	0,00950	0,553283	0,547	0,008267	0,45186
ENSG00000150457	*LATS2*	-0,572	0,00817	0,522397	-0,570	0,008331	0,45186
ENSG00000260804	*LINC01963*	-0,664	0,00976	0,556927	-0,675	0,008413	0,45250
ENSG00000160172	*FAM86C2P*	-0,656	0,00821	0,523212	-0,609	0,008424	0,45250
ENSG00000233493	*TMEM238*	-0,906	0,00837	0,528936	-0,899	0,008431	0,45250
ENSG00000106066	*CPVL*	-0,630	0,00574	0,430266	-0,590	0,008457	0,45318
ENSG00000204934	*ATP6V0E2-AS1*	-0,832	0,00696	0,472704	-0,807	0,008565	0,45625
ENSG00000162738	*VANGL2*	0,864	0,00923	0,546800	0,850	0,008578	0,45625
ENSG00000184731	*FAM110C*	-0,651	0,00816	0,522045	-0,643	0,008609	0,45715
ENSG00000117984	*CTSD*	-0,570	0,00978	0,557372	-0,577	0,008711	0,46143
ENSG00000177570	*SAMD12*	-0,776	0,00629	0,452129	-0,742	0,008718	0,46145
ENSG00000141579	*ZNF750*	0,765	0,00884	0,543516	0,760	0,008799	0,46416
ENSG00000140853	*NLRC5*	0,666	0,00593	0,436781	0,615	0,008857	0,46416
ENSG00000132965	*ALOX5AP*	5,912	0,00894	0,543516	5,914	0,008941	0,46416
ENSG00000270419	*CAHM*	5,912	0,00894	0,543516	5,914	0,008941	0,46416
ENSG00000184502	*GAST*	6,079	0,00894	0,543516	6,081	0,008941	0,46416

ENSG00000258602	*LINC01629*	6,079	0,00894	0,543516	6,081	0,008941	0,46416
ENSG00000169258	*GPRIN1*	-0,563	0,00885	0,543516	-0,560	0,008979	0,46501
ENSG00000111057	*KRT18*	0,554	0,00729	0,486812	0,538	0,009080	0,46903
ENSG00000188064	*WNT7B*	-0,642	0,00701	0,475131	-0,620	0,009086	0,46903
ENSG00000101782	*RIOK3*	-0,584	0,00690	0,472689	-0,558	0,009138	0,47016
ENSG00000178809	*TRIM73*	0,748	0,00396	0,340301	0,600	0,009250	0,47298
ENSG00000163754	*GYG1*	-0,564	0,00961	0,553283	-0,561	0,009549	0,48380
ENSG00000165169	*DYNLT3*	-0,586	0,00847	0,531356	-0,570	0,009589	0,48380
ENSG00000177098	*SCN4B*	-2,738	0,00961	0,553283	-2,734	0,009611	0,48380
ENSG00000083817	*ZNF416*	2,507	0,00961	0,553283	2,511	0,009611	0,48380
ENSG00000117152	*RGS4*	-1,874	0,00663	0,460736	-1,725	0,009626	0,48380
ENSG00000114757	*PEX5L*	0,733	0,00768	0,501041	0,707	0,009717	0,48648
ENSG00000144369	*FAM171B*	-0,598	0,00744	0,492701	-0,573	0,009839	0,49051
ENSG00000143494	*VASH2*	0,905	0,00992	0,562715	0,909	0,009920	0,49248
ENSG00000151917	*BEND6*	-0,918	0,00764	0,499236	-0,878	0,009959	0,49267
ENSG00000101003	*GINS1*	0,523	0,01411	0,671773	0,545	0,010261	0,49782
ENSG00000280549	*PADI3*	-1,090	0,00868	0,542211	-0,758	0,013567	0,58917

Supplementary Table S5. Genes altered in common by silencing *ZNF518B*, *EHMT2* and *EZH2* in HCT116 cells, using a p-value < 0.01

Intersection	Number of genes	Gene Symbol
si*EZH2* & si*EHMT2*	326	*ABCA3, ABHD12B, ABHD4, ABTB2, ACSS2, AIDA, AKR1B10, ALOX5AP, AMDHD1, ANXA6, APLP2, APOBEC3B, APP, ARHGEF40, ARID3B, ARL14, ARL3, ATP10D, ATP2A3, ATP2B2, ATP8B2, BCO2, BIRC3, BMP5, C1orf21, C1orf61, CACNB3, CACNG7, CAPN2, CCDC3, CCDC71L, CCND1, CCNG1, CD24, CD276, CD63, CD99, CDK8, CDKN2B, CEMIP2, CENPW, CFAP70, CHPF, CHRNB1, CILP2, CNIH2, CNTN1, COL12A1, COL4A1, CORIN, CORO6, CPE, CPLX1, CPVL, CR2, CREB3L2, CRLF1, CSRP1, CST3, CXCL16, CYFIP2, CYP4F11, CYP4F2, CYP4F3, CYSTM1, DIPK1A, DNAJC3, DNAJC9-AS1, DOC2B, DPP4, DPYSL5, DTX4, DUSP6, DYNLT3, EGFR, EGR1, EGR3, EID3, ELF3, EME2, EML1, ERO1B, ETNK2, EXT2, FAM171A1, FAM171B, FAM8A1, FANCF, FANK1, FBN1, FDFT1, FDPS, FER1L4, FGF2, FGFBP1, FLG, FLG-AS1, FLJ34503, FLOT2, FNDC3B, FOXD3-AS1, FRAS1, FRZB, FUCA1, FUOM, FXYD5, FYCO1, GABRD, GAL3ST4, GAS6, GAST, GCAT, GCLC, GDAP2, GLI2, GMPR, GNG4, GPR157, GRM4, GSTT2, GXYLT2, GYG1, HAPLN3, HDAC6, HEPH, HIP1, HKDC1, HLA-B, HLA-C, HMGA2, HMGCS1, HNF4G, HOOK1, HPCAL4, HPD, HPRT1, HPS3, HRNR, IFI6, IGF2BP2, IGSF3, IL31RA, IL6ST, INSIG1, ITGA2, ITGA6, ITGB1BP2, JAZF1, JUN, JUND, KCND1, KCTD12, KIF21B, KITLG, KLF15, KLF9, KRT80, L1CAM, LAMA3, LAMB3, LAMP2, LATS2, LEAP2, LIMD2, LINGO1, LMO3, LOC101928994, LOC101929705, LOC105371664, LOC339685, LOC400499, LOC653653, LOXL2, LRRCC1, LRRN3, LSS, LTB4R2, LUCAT1, LYPD6, LYSMD2, MALAT1, MALL, MAP1LC3B, MAP2, MAP3K14, MAPK8IP2, MCF2, MCUB, MDK, MEA1, MESD, MET, MLANA, MLEC, MOB4, MRPL36, MVD, NAGLU, ND4, ND5, ND6, NEAT1, NEB, NECTIN3, NFKBIA, NKX6-1, NOTUM, NPC1, NPIPB11, NPR2, NPTXR, NUCB2, OGDHL, OLFML2B, OLMALINC, OR51B6, OR52D1, P2RY11, PALM3, PANX2, PDGFRL, PDIA3, PDIA4, PDIA6, PEG10, PHOSPHO1, PIK3AP1, PIM1, PLAAT3, POMGNT2, PPDPF, PPP2R2C, PROCR, PROZ, PTTG1IP, RAB15, RBP1, RCAN1, RCN3, RFFL, RFK, RNASE1, RNF135, RNF182, RNF207, RSKR, RTN4R, RTN4RL2, RUSC1-AS1, S100A2, S100A3, SAT1, SCAMP5, SCARNA21, SCARNA5, SCARNA7, SCARNA9, SCN3A, SCNN1A, SDC1, SDF2L1, SEC24D, SEL1L, SELENON, SEMA3E, SEPTIN3, SERINC2, SERP1, SEZ6L2, SFN, SLAIN2, SLC25A22, SLC26A7, SLC2A1, SLC35B1, SLC35D1, SLCO2B1, SMARCD1, SNN, SNX24, SQOR, SRP14-AS1, SRRM3, ST3GAL3, ST6GAL1, STARD13, STRADB, SURF4, TAS2R4, TBL1XR1, TGFBI, TGFBR1, TMCO1, TMEM37,*

		TMEM38B, TMEM45A, TMEM80, TMOD2, TMPRSS9, TNFRSF8, TRIM73, TRIQK, TSPAN13, TSPAN18, TSPAN5, TTYH3, TXNDC5, TXNRD3, UBAP1L, UPP1, UQCR10, UST, VANGL1, VANGL2, VEGFA, WFS1, ZBED2, ZCCHC14, ZCCHC24, ZFP36L1, ZNF185, ZNF267, ZNF41, ZNF585B, ZNF750, ZNF804A
si*EHMT2* & si*ZNF518B*	28	*ADM, AGO2, C5AR1, DHRS3, EIF5A2, EXPH5, GINS1, GNAZ, KCTD2, KRTAP2-3, LGALS1, MEF2C, MSRB3, NAV3, NIP7, NR4A2, PADI3, PAX2, PLA2G7, PNPLA3, RAB8B, SETSIP, SLC39A14, SLC6A17, SOBP, STMN3, TMTC1, ZNF503*
si*EZH2* & si*ZNF518B*	61	*ABCC3, ABHD10, ACVR1C, AGPAT5, AOC2, ARHGAP29, ATL3, ATP11C, BCL2L1, C22orf46, CA13, CAMK2D, CCNE1, CDK6, CHAC1, CRABP2, CREB5, CRIM1, CTSC, CYBRD1, DCLK1, F2RL1, GDA, HOXB6, IL1RAP, KLF2, LETM2, MAMDC2, MBNL3, MOB1A, NEDD4, NETO2, NIPAL2, NR6A1, PAPSS2, PARM1, PCGF5, RAB37, RALGPS2, RAPGEF1, RGS5, SESN3, SHTN1, SLC27A6, SLC7A8, SNAI2, SNX7, ST8SIA6, SUMO3, SYTL2, TACC1, TGM2, TMOD1, TNIK, TNS4, TOM1L2, TOMM34, TRIM14, TWSG1, UBASH3B, ZDHHC2*
si*EZH2* & si*EHMT2* & si*ZNF518B*	29	*ADAM19, ANTXR2, AXIN2, BMP2K, CAPN6, CLDN4, DDAH1, DKK1, ELK3, FBXO21, HLA-DRA, HOXB-AS3, LRRC8B, NRP1, NT5E, PCTP, RGS4, S100A14, S100A4, SCD5, SLC16A7, SLC22A17, SMAD6, SRPX, TGFB1, TMEM200A, TSPAN7, UBE2L6, VCAN*

Supplementary Table S6. Primers used for RT-qPCR

Gene	Primers	
	Forward	Reverse
ZNF518B (Whole)	GGGCCTGAGGTTGTGAAACT	AAAACCGTGGCAAGTCCCAT
ZNF518B (isof. 1)	GCTACAGGCAGGAATGTTACC	CGCAGTAGGTGCATGATCCC
ZNF518B (isof. 2)	CTGCCGGTGTTACCTGGAAT	GCGCAGCTACTTCTTGGGT
ACTB	GTGCTATCCCTGTACGCCTC	GAGGGCATACCCCTCGTAGA
DDAH1	CAAAAGGACAAATCAACGAGGTG	TGTGCAGATTCACTAGACCCAA
SNAI1	ACCACTATGCCGCGCTCTT	GGTCGTAGGGCTGCTGGAA
CDH1	GTCAGTTCAGACTCCAGCCC	AAATTCACTCTGCCCAGGACG
RBL2	GGAGGAAATTGGGACTCTCTCA	AGACGACTCAAGCTATGCGTA
CREB5	TGGGACACATGATGGAGATGA	GGTGTGCATGAAGGTGGGAA
SRPX	ATCAAGGTGAAGTATGGGGATGT	GTTTGACTGGCAGATCAGTAGG
THBS1	GCCATCCGCACTAACTACATT	TCCGTTGTGATAGCATAGGGG
NRP1	ACGTGGAAGTCTTCGATGGAG	CACCATGTGTTTCGTAGTCAGA
BCAM	GAGGTGCGCTTGTCTGTACC	GCATATAATGGTCGTGGGTTCC
TOMM34	TGCATCAAAGATTGCACTTCAGC	GCAGCACAGTCTTATAGTCAACA
PTBP2	TGCAGTTGGCGTGAAGAGAG	ATGCTGCTCATATTAGAGTTCGG
S100A14	GAGACGCTGACCCCTTCTG	CTTGGCCGCTTCTCCAATCA
ZDHHC2	AGCCAAGGATCTTCCCATCT	TTCACCCATGGACAATGATG
PADI3	TTTGGAGATCATCGTGGTCA	CGCAATCCAGAGAGATGTCA
EFNA5	AGGACTCCGTCCCAGAAGAT	CCCATCTCTTGAACCCTTTG
RGS4	CTTTGATGAGGCCCAGAAGA	TGCTGAACTCTTGGCTCCTT
HLTF	GTGCAATTTGCCTGGATTCT	TAGCATGTGGCTGCTCATTC
KAT2B	GACTGCGATCTCCCAATGAT	CAAACACCTGTGTGGTTTCG

Supplementary Table S7. Primers used for quantitative PCR in ChIP analyses

Gene	Primers	
	**Forward**	**Reverse**
ZDHHC2 p	GGATTCACAGCCAAAAATGC	GCAAGGAGCCTCCCTTTACT
ZDHHC2 c	AGGTGAAGACTGCTGCACTG	GGTCGAAACACTGGGACAAT
PADI3 p	TACCTGTGGGGACTTGCTTC	AGCTGAGAGCTCAGGATTGG
EFNA5 p	CTGGCAAGAAGTGGAAAGGG	CGCAAGTCATTAACGAGGGG
EFNA5 ex	CAGAAACACCAGCGTCAACA	TCTCTCTGTCCCTTTGCCTG
EFNA5 c	TACTTTTGCTCCCACACCTG	CTCTGAAGCGCCTGGAAATC
EFNA5 c (3')	GGTGTTCCAAGACCCTGATG	GCCTTTTGGCAATCCTACTG
RGS4 c1	CGGTCGTAGCTGGGCTATAA	TTCGGCTTTGAGCGTACTTT
RGS4 c2	CTGCTCTTCCCTTTCCTCCT	CCCACCACCTATGTGACCTT
RGS4 c3	AGAAAGAAGCCCCAAGAAGC	AGCAGTTCCTGCTGTGAGGT
HLTF p	TGGGAGCAGATTCCTTTGAC	CAAGGCTTGTGAAGTGGATG
HLTF c	AGCGAGACTCCGTCTCAAAA	GCTCAGCTTTAAAGTCAAACTAACG
KAT2B p	CTGTAGGAGCTCGCTGGAGT	ACACAGGAAGGGATCGATTG
KAT2B c	CTACCATGCCCACTCTCCAA	TTCAGTGTGCTCTTTCTGTGC

p: promoter; c: control regions; ex: exon.
